# A comparison of mental health of student and not student emerging adults living in Ecuador

**DOI:** 10.1038/s41598-023-27695-0

**Published:** 2023-01-27

**Authors:** Clara Paz, Chris Evans

**Affiliations:** 1 Escuela de Psicología y Educación, Universidad de las Américas, Quito, Ecuador; 2 Department of Psychology, University of Roehampton, London, UK

**Keywords:** Psychology, Epidemiology

## Abstract

Most of the studies about mental health and quality of life of emerging adults have been conducted in developed countries and non-students’ population has been neglected, limiting the generalisation of the results to other socioeconomic realities. This paper reports the results of an observational study on differences between the two cohorts (students vs non-students) both on mental health and quality of life measures but also on demographic, lifestyle and mental health variables in emerging adults living in a middle-income country. Associations between variables and interactions in the prediction of both outcomes scores were explored to understand how much other variables may contribute to differences between the two groups. We found poorer mental health and worse health-related quality of life in the students than the non-students, although effect sizes were small. Differences between the groups on some sociodemographic predictor variables were statistically significant, showing fairly strong effects, for social status, sleeping hours and parenting, however, none of the predictor variables showed confounding with group effects on both outcomes. Developing countries are growing and work forces are changing, creating a huge global need to understand these changes and the effects on the mental health and quality of life of this evolving population.

**Registration:** ClinicalTrials.gov (NCT04596345).

## Introduction

Emerging adulthood is a distinct life stage^1^ defined roughly by the demographic variable of age (18–29 years)^2^ but crucially by being a stage of identity-exploration, instability, self-focus, feeling in-between and possibilities^3^. In this stage of the life, individuals delineate their professional future and their potential route of success by either pursuing education, getting employment or by combining both^2^. In 2000 when the theory of emerging adulthood was proposed, Jeffrey Jensen Arnett^1^ tried to explain a new life course in industrialized societies with specific developmental characteristics, his theory was based on data coming mainly from the United States of America but as work on this development period has emerged from other countries, the importance of this life stage has been underlined in other countries and cultures^2,4^ though it is clear that cultural, political and socio-economic factors can shape the stage differently.

During emerging adulthood, mental health struggles often appear for the first time^5^. Furthermore, symptoms of many formal mental disorders first appear in emerging adults^6,7^ although help seeking and professional diagnoses come later in life^8^. Studies conducted in wealthy countries including the United States^9^, the Netherlands^10^ and New Zealand^11^ indicate that the 12-month prevalence for depression ranges from 8.3 to 12.4%, anxiety disorders from 19.4 to 22.3% and alcohol abuse from 7.1 to 18.4% among people between 18 and 33 years. There is a danger in focusing this period purely in a frame of symptomatology and diagnoses or even in a wider frame of well-being, that the social situations of this life phase are ignored. This study aims to locate mental health (MH) problems and quality of life (QoL) in emerging adults within the changes that individuals face over one year. It also aims to start correcting the neglect of non-student emerging adults and of less wealthy (“low and middle income”, LMIC) countries.

Studies comparing emerging adults’ students and non-students show varied findings. On one side, the results of some studies indicate that students have better MH than non-students, these studies have been conducted in Australia^12^, Canada^13^ and the United Kingdom^14^. These studies used large national survey data to compare the outcomes of both populations, the age of the participants ranged between 15 to 25 years. The authors claim that the better MH of the students might be the result of increased MH literacy in the students' population contributing to better identification of MH issues^13^ and also because they have better access to MH services^14^. On the other side, some studies indicate that student status is associated with poorer MH^15–19^, but just three studies^16–18^ compared both groups directly, two of them^16,17^ were conducted during the COVID-19 pandemic lockdown in France^16,17^ and one study was conducted in the USA with Latinx population^18^. In both studies conducted in France, the students' populations were younger than non-students confounding the comparison. Other studies indicating that students have worse MH than non-students also argued this by comparing their student survey results with results from general adult populations but again not having the same age range^15,19^. There is also one study conducted by Blanco et al.^20^ in the USA that found no significant differences in the overall rate of the presence of psychiatric disorders between students and non-students aged 19 to 25 years.

The lack of consistency of these findings underlines the need for studies directly comparing MH between students and non-students of the same age. Moreover, all existing studies were conducted in high-income countries, limiting the generalisation to other socioeconomic realities, such as those of low and middle-income countries, where the number of persons enrolled in tertiary education has been growing markedly in recent decades, from less than 6% of the population in the '70s to almost 30% of the population in 2014^21^.

This paper reports baseline findings of a project addressing both the lack of direct student versus non-student comparisons and coming out of a middle-income country more representative of the world's population than the existing work almost entirely from the developed world: Ecuador. This paper reports on differences between the two cohorts (students vs non-students) both on MH and QoL measures but also on demographic, lifestyle and MH seeking variables. Associations between variables, and interactions in the prediction of the MH and QoL scores are explored to attempt to unravel how much other sociodemographic variables may contribute to differences between the two groups.

## Methods

This is the baseline of an observational cohort study for further details see the study protocol^22^.

### Ethical approval

All procedures contributing to this work comply with the ethical standards of the relevant national and institutional committees on human experimentation and with the Helsinki Declaration of 1975, as revised in 2008. This study received ethical approval by the Comité de Ética y Bioética (Ethics and Bioethics Committee) of the Universidad de Las Américas, Quito-Ecuador (2020-0807).

### Consent to participate

Participants were informed of the nature and purpose of the study and provided online consent.

### Participants

Participants are emerging adults (18–29 years) living in Quito, Ecuador having the competence to read and understand Spanish. There are two subsamples: students and non-students (hereafter "groups"). The inclusion criterion for the student group was to be enrolled in a university program and the non-student group was defined by an exclusion criterion: not being enrolled in any type of education program at baseline.

### Referential data

To assess comparability with the population of Quito we compared our sample with pertinent data from the census data for adults aged 18 to 29 living in Quito at the time of the last existing census. The aim was to assess sampling bias as we knew a truly random, representative sample was impossible.

### Measures

Sociodemographic, lifestyle and MH-related characteristics, apart from the status as student or non-student, basic demographic items were gender, age, housing status, financial issues, parenting and caregiver responsibilities. MH status was collected by asking by diagnoses and medication. Lifestyle questions focused on the average sleeping hours, frequency in maintaining everyday routines, frequency of exercising every week and the average number of hours spent on social media per day.

MH was measured using the responses given to the Clinical Outcomes in Routine Evaluation-Outcome Measure (CORE-OM^23^), a 34-item self-reported measure of psychological distress. The CORE-OM was originally developed in the UK and translated into Spanish in Spain^24^ but an exploration of the psychometric properties of that translation has been completed in Ecuador^25^, showing that the properties are adequate and similar to those found with samples from UK and Spain.

Health-related QoL was assessed using a well-known instrument, the EuroQol five-dimension-three-level (EQ-5D-3L), “EQ” hereafter^26^. This measure is a 5-item self-reported questionnaire that evaluates five dimensions of the quality of life: Mobility, Self-Care, Usual Activities, Pain and Discomfort, Anxiety and Depression. Participants rate each dimension based on 3 levels, which allow obtaining a 5-digit composite number that expresses participants’ health state. The health states can be later converted into a health-related quality of life index value using Ecuador’s specific scores^27^. This index is complemented by a Visual Analogue Scale (EQ VAS) that evaluates the perceived health state of the participant being 0 for the worst imaginable health and for 100 the best imaginable health.

Evaluation of relevant vital events was considered using the What’s Going On? (WGO) questionnaire, which is a self-reported measure to identify relevant vital events which are rated for impact on the person’s life. In the present analyses, the two items that assess the overall experience of lived events in the last two months were included, “good things happened to me these last two months” (WGO + ve) and “bad things happened to me these last two months” (WGO-ve). These two items are rated using a 7-point Likert scale from strongly agree to strongly disagree.

### Procedures

Participants of the student group were recruited by an institutional email inviting them to participate in the study, these participants were in turn asked to invite non-students to participate in the study. Data were collected using “formr” system^28^ based on R^29^. This generates secure online survey forms, starting with an explanation of the study. Participation was online through these forms. Participants were asked to complete the survey on seven occasions. This report, as per protocol analyses of the baseline data in which participants provided sociodemographic information, health and lifestyle-related information, and completed the CORE-OM, the EQ and the WGO questionnaire.

### Registration

The full study was registered in ClinicalTrials.gov (NCT04596345), and that registration included a Data Analysis Plan specifying the analyses including those of the baseline data. Recruitment of participants started on October 2020 and baseline data collection terminated in January 2021.

### Data analysis

All analyses were conducted using R^29^. The analyses are reported in eight blocks and are exploratory, descriptive rather than focused on hypothesis testing.

1. The analyses started with a comparison with census data.
2. Psychometric exploration of the multi-item dependent variables (CORE-OM, and EQ) included internal consistency assessed using Cronbach’s alpha and McDonald's omega coefficient. Bootstrap 95% confidence intervals (CIs) are not reported for omega as this is not a stable statistic as the factor model being tested is sample dependent so can vary across bootstrap resamples. Principal Component Analysis (PCA) was also used to explore the structure of the item covariances.
3. Simple effects of group on dependent variables. This was then put in context through the next four blocks of analyses.
4. Summary descriptions of the predictors (sociodemographic, lifestyle and MH variables).
5. Exploration of group differences between the groups (students vs. non-students).
6. Evaluation of associations between all predictors within each group.
7. Exploration of univariate associations between each predictor and each dependent variable within the groups.
8. Each of the predictors which showed significant relationships with the dependent variables were included, with group, in bivariate linear regression models for each dependent variable to assess the interaction between the sociodemographic predictors with group.
9. Finally, we describe distributions of the dependent variables to provide referential data for emerging adults in Quito potentially generalisable to Ecuador.

The multiple steps recognise that there will probably be differences between the groups on many of the socio-demographic variables, many systematic associations within those variables, and potentially many statistically significant "predictive" relationships between socio-demographic variables and both MH and QoL. Ascribing any causal relationships predicting MH and QoL is impossible in such a survey design, but these complexities make even describing simple associations, such as between group and MH and/or QoL potentially misleading without thorough exploration. We have summarised the main findings here and reference Supplementary Materials with more extensive findings where appropriate.

Where significance tests were reported the criterion was set at p < 0.01 to reduce the likely rate of Type I errors recognizing the great number of comparisons. However, as noted above, the approach is descriptive rather than one of formal null hypothesis testing and whenever possible for any sample statistics 95% bootstrapped confidence intervals (CI) are reported, these are shown in brackets “[LCL, UCL]”. Where applicable effect sizes are given to indicate the strengths of relationships with group, Cramer's V is used for categorical variables against group and Hedges' g for the effect of group against continuous variables and eta squared is used for all analyses in the exploration of interactions between variables in impact on the dependent variables. Confidence intervals for centiles are constructed using the quantileCI R package^30^ which implements the methods of Nyblom^31^.

The data and code used to perform these analyses are available at: osf.io/4fsyn.

## Results

In total, 1168 emerging adults met the inclusion criteria, agreed to participate in the study and completed the sociodemographic information. Of these 669 (57.3%) were non-students and 499 (42.7%) were students. However, only 1076 also completed the MH and QoL measures (non-students = 590 and students = 486: a non-significant difference in non-completion between groups χ^2^ (1, n = 1354) = 0.73, p = 0.393). These 1076 are the participants whose data is reported in this paper. The mean age of the participants was 22.9 years, 365 (59.0%) were female, 437 (40.6%) male and 4 (0.37%) declared as "other".

### Comparison with 2020 census data

The census^32^ had data for 508,723 18- to 29-year-old emerging adults in Quito of whom 35% were studying (though not necessarily for an undergraduate university degree). Of 495,197 (97.3%) who had answered the census question about having the highest level of education the modal highest level achieved was “Undergraduate”, n = 194,658, 38.3% of those responding.

The census used a binary classification of gender with 50.99% female, significantly lower than in our sample (59%; χ^2^ (1, n = 1072) = 44.88, p < 0.001, odds ratio (OR): 1.4 [1.24, 1.58]). Recategorising social status categories to align census and survey data (to “divorced”, “partnered” and “single”) gave census rates of 0.7%, 41.8% and 57.5% and sample rates of 0.6%, 3.9% and 95% (χ^2^ (2, n = 1076) = 641.10, p < 0.001, OR for married:single, 0.03 [0.01, 0.05]). Finally, the census data gave numbers of children for women only. The rate of not having any children of 91.0%, 95% [88.72%, 93.15%] was dramatically different from 51.34% for the census data (OR: 9.77 [7.5, 13.01]).

### Psychometric properties of the dependent variables

Second, a psychometric exploration of the dependent variables was conducted. The CORE-OM showed excellent internal consistency for each group (non-students α = 0.935 [0.928, 0.943], ω = 0.946; students α = 0.939 [0.932, 0.946], ω = 0.947). As expected for such a short scale, the internal consistency of the EQ was poor for both groups (non-students α = 0.498 [0.427, 0.561], ω = 0.576; students α = 0.410 [0.303, 0.496], ω = 0.589). Exploration of the PCA is included in the Supplementary Materials but suggested a three-component structure for the CORE-OM (positively worded items, negatively worded items and risk items) and a one-component structure for the EQ. As expected, these findings suggested that risk items of the CORE-OM are sufficiently distinct from the other items that the risk score should be interpreted separately so we report analyses of three scores: the total score (as this is most widely reported in the existing literature), the non-risk score and the risk score. For the EQ we analyse the index scores calculated from the 5 items and separately the EQ-VAS, as suggested by the guidelines^26^.

Looking at convergent validity (Table 1) score intercorrelations were very similar for each group and for the full sample (matrices for the groups are in the Supplementary Materials). As anticipated, the CORE-OM scores attained a negative and moderate correlation with the EQ scores.

**Table 1:** Pearson’s correlations and 95% bootstrapped confidence intervals of the dependent variables.

	CORE-OM Total	CORE-OM Risk	CORE-OM NR	EQ-Index	EQ-VAS	WGO+ ve
CORE-OM Risk	0.67 [0.63, 0.70]					
CORE-OM NR	1.00 [0.99, 1.00]	0.60 [0.56, 0.63]				
EQ-Index	− 0.58 [− 0.63, − 0.53]	− 0.42 [− 0.49, − 0.35]	− 0.57 [− 0.62, − 0.52]			
EQ-VAS	− 0.55 [− 0.60, − 0.49]	− 0.35 [− 0.41, − 0.28]	− 0.55 [− 0.60, − 0.50]	0.42 [0.36, 0.48]		
WGO + ve	− 0.49 [− 0.54, − 0.44]	− 0.30 [− 0.36, − 0.24]	− 0.50 [− 0.55, − 0.45]	0.27 [0.20, 0.33]	0.26 [0.19, 0.33]	
WGO-ve	− 0.47 [− 0.51, − 0.42]	− 0.25 [− 0.30, − 0.20]	− 0.47 [− 0.52, − 0.42]	0.30 [0.25, 0.35]	0.27 [0.22, 0.33]	0.29 [0.24, 0.35]

*CORE-OM* Clinical Outcomes in Routine Evaluation, *NR* Non-risk items score, *EQ-Index* EuroQol five-dimension-three-level Index, *EQ-VAS* EuroQol Visual Analogue Scale, *WGO+ve* What’s Going On score for “good things happened to me these last two months”, *WGO−ve* What’s Going On score for “bad things happened to me these last two months”.

### Simple effects of group on the measure scores

Third, we compared dependent variable scores between both groups (Table 2). The CORE-OM total score and the non-risk items of the CORE-OM are significantly different between groups with poorer MH for students than for non-students but with small effect sizes. No significant differences were found in the risk score. The EQ index and the EQ-VAS were significantly different between groups with better health related QoL for the non-students than the students, but the effect sizes were very small. Neither of the WGO items' scores were different between groups.

**Table 2:** Mean, SD, distribution of the dependent variables by group and mean difference and effect sizes (Hedges’ g) of the comparison between groups.

	Non-students (N = 590)	Students (N = 486)	Mean difference [95%CI]	Hedges’ g [95%CI]
CORE-OM total				
Mean (SD)	0.99 (0.57)	1.13 (0.62)	0.14 [0.07,0.21]	− 0.24 [− 0.35, − 0.12]
Median [min, max]	0.882 [0.03, 2.97]	1.06 [0, 3.26]		
CORE-OM non-risk (NR)				
Mean (SD)	1.16 (0.64)	1.32 (0.69)	0.17 [0.08, 0.025]	− 0.25 [− 0.37, − 0.13]
Median [min, max]	1.07 [0, 3.21]	1.29 [0, 3.68]		
CORE-OM risk				
Mean (SD)	0.23 (0.39)	0.24 (0.39)	0.009 [− 0.04, 0.06]	− 0.02 [− 0.14, 0.09]
Median [min, max]	0 [0, 2.67]	0 [0, 2.00]		
EQ-VAS				
Mean (SD)	76.10 (22.3)	70.20 (23.2)	− 5.88 [− 8.84, − 3.21]	0.26 [0.14, 0.38]
Median [min, max]	82.0 [1.00, 100]	74.5 [1.00, 100]		
EQ-index				
Mean (SD)	0.95 (0.06)	0.94 (0.07)	− 0.01 [− 0.02, − 0.01]	0.27 [0.15, 0.39]
Median [min, max]	0.96 [0.63, 1.00]	0.96 [0.42, 1.00]		
WGO positive				
Mean (SD)	5.83 (1.27)	5.65 (1.33)	− 0.17 [− 0.34, -0.02]	0.14 [0.02, 0.26]
Median [min, max]	6.00 [1.00, 7.00]	6.00 [1.00, 7.00]		
Missing	78 (11.7%)	13 (2.6%)		
WGO negative				
Mean (SD)	3.81 (1.76)	3.61 (1.77)	− 0.20 [− 0.41, − 0.02]	0.12 [− 0.005, 0.24]
Median [min, max]	3.50 [1.00, 7.00]	3.00 [1.00, 7.00]		

*CORE-OM* Clinical Outcomes in Routine Evaluation, *NR* Non-risk items score, *EQ-Index* EuroQol five-dimension-three-level Index, *EQ-VAS* EuroQol Visual Analogue Scale, *WGO+ve* What’s Going On score for “good things happened to me these last two months”, *WGO−ve* What’s Going On score for “bad things happened to me these last two months”.

### Disentangling associations and interactions

Fourth, we described a total of 19 sociodemographic variables. Table 3 shows the descriptive statistics of all the categorical predictors by group (more detailed analyses are in the Supplementary Materials). Any survey is liable to find that any simple comparisons, such as between the two groups here, are confounded by associations between variables and potentially by interactive effects on the dependent variables.

**Table 3:** Descriptive statistics, p values and effect sizes of the categorial predictors by group.

	Non-students (N = 590)	Students (N = 486)	p	OR [CI]
Gender			0.0028	
Female	321 (54.4%)	314 (64.6%)		
Male	266 (45.1%)	171 (35.2%)		0.66 [0.51, 0.84]
Other	3 (0.5%)	1 (0.2%)		0.37 [0.01, 3.22]
Employment*				
Home tasks	81 (13.7%)	0 (0%)		
Neither studying nor working	152 (25.8%)	0 (0%)		
Working	357 (60.5%)	0 (0%)		
Student	0 (0%)	384 (79.0%)		
Studying and working	0 (0%)	102 (21.0%)		
Living with			< 0.001	
Partner and/or kids	62 (10.5%)	14 (2.8%)		
Friends	8 (1.3%)	2 (0.4%)		1.16 [0.15, 5.44]
Living alone	36 (6.1%)	13 (2.7%)		1.59 [0.66, 3.81]
Parents	442(74.9%)	418 (86.0%)		4.15 [2.35, 7.84]
Relatives	33 (5.6%)	27 (5.6%)		3.57 [1.67, 7.94]
Other	9 (1.5%)	12 (2.5%)		5.74 [2.03, 17.0]
Housing			< 0.001	
Borrowed	49 (8.3%)	26 (5.4%)		
Other	5 (0.9%)	9 (1.8%)		3.30 [1.01, 12.06]
Own home	386 (65.4%)	370 (76.1%)		1.80 [1.10, 3.00]
Renting	150 (25.4%)	81 (16.8%)		1.02 [0.59, 1.78]
Civil status			0.0048	
Married	33 (5.6%)	9 (1.8%)		
Single	557 (94.4%)	477 (98.1%)		3.10 [1.52, 6.99]
Parent			< 0.001	
No	524 (88.8%)	475 (97.7%)		
Yes	66 (11.2%)	11 (2.3%)		0.19 [0.09, 0.34]
Children under 18 years			< 0.001	
No	525 (88.9%)	476 (97.9%)		
Yes	65 (11.0%)	10 (2.0%)		0.17 [0.08, 0.32]
Children under 6 years			< 0.001	
No	537 (91.2%)	477 (98.3%)		
Yes	52 (8.8%)	8 (1.7%)		0.18 [0.08, 0.36]
Elderly caregiver			< 0.001	
No	553 (93.7%)	426 (87.6%)		
Yes	37 (6.3%)	60 (12.4%)		2.10 [1.37, 3.25]
Main carer			< 0.001	
No	538 (91.2%)	476 (97.9%)		
Yes	52 (8.8%)	10 (2.1%)		0.22 [0.10, 0.42]
Diagnosis of mental health disorder			0.13	
No	445 (75.4%)	346 (71.9%)		
Yes	145 (24.6%)	140 (28.8%)		1.24 [0.95, 1.63]
Having medication for mental health disorder			0.89	
No	113 (77.9%)	111 (79.3%)		
Yes	32 (22.1%)	29 (20.7%)		0.92 [0.52, 1.63]
Diagnosis of chronic disease			0.004	
No	566 (95.9%)	445 (91.6%)		
Yes	24 (4.1%)	41 (8.4%)		2.17 [1.30, 3.69]

*Dependence test between employment and group was not conducted due to being part of the non-student group exclude the possibility to be studying and to be studying and working. However, we present the description of the categories for informative purposes.

Fifth, we compared the predictors between the groups. Our analyses showed that 10 variables (gender, civil status, living situation, housing, parenting, having children under 18, having children under 6 years, taking care of older adults, being the main carer and having a diagnosis of chronic disease) out of the 12 categorical variables differentiated significantly between groups at p < 0.01. Clearly employment is a direct function of the group membership rather than an association but the other statistically significant associations in Table 3 are informative. MH variables (diagnosis and medication) were not statistically significantly associated with group. Significance tests with p values are reported but arguably the effect sizes are more important and only reached the level of "small" in Cohen's^33^ categories. For the numeric predictors, four (age, financial dependency, sleeping hours, and average exercising days per week) out of seven presented significant differences (p < 0.01) between groups (Table 4). Students were significantly younger than non-students. In contrast to the findings for the categorical variables, two effects here were substantial: the difference in sleeping hours had Hedges' g of 0.54, a medium effect size and a difference in means of about 30 min from 6.47 h for the students and 7.15 and the effect sizes for age and rated financial dependency were both classified as strong effects.

**Table 4:** Descriptive statistics, mean differences and effect sizes (Hedges’ g) of the numeric predictors by group.

	Non-students (N = 590)	Students (N = 486)	Mean difference [95% CI]	Hedges’ g [95% CI]
Age				
Mean (SD)	23.1 (2.79)	21.7 (2.15)	− 2.17 [− 2.47, − 1.86]	0.86 [0.73, 0.98]
Median [min, max]	24.0 [18.0, 29.0]	21.0 [18.0, 29.0]		
Financial dependency				
Mean (SD)	2.03 (0.75)	2.65 (0.59)	0.62 [0.54, 0.70]	− 0.91 [− 1.04, − 0.79]
Median [min, max]	2.00 [1.00, 3.00]	3.00 [1.00, 3.00]		
Financial distress				
Mean (SD)	1.29 (0.83)	1.37 (0.94)	− 0.07 [− 0.04, − 0.179]	− 0.09 [− 0.21, − 0.04]
Median [min, max]	1.00 [0.00, 3.00]	1.00[0.00, 3.00]		
Average number of sleeping hours per day				
Mean (SD)	7.15 (1.22)	6.47 (1.33)	− 0.68 [− 0.83, − 0.52]	0.54 [0.42, 0.66]
Median [min, max]	7.00 [4.00, 15.0]	6.00 [1.00, 12.0]		
Days exercising per week				
Mean (SD)	2.58 (2.00)	2.22 (1.94)	− 0.36 [− 0.59, − 0.12]	0.18 [0.06, 0.30]
Median [min, max]	2.5 [0.00, 6.50]	2.5 [0.00, 6.50]		
Average number of hours using social networks per day				
Mean (SD)	4.73 (3.18)	4.37 (2.72)	− 0.36 [− 0.71, 0.01]	0.12 [0.001, 0.24]
Median [min, max]	4.00 [0, 24.0]	4.00 [0, 20.0]		
Maintaining a routine				
Mean (SD)	2.42 (1.13)	2.35 (1.04)	− 0.07 [− 0.21, 0.07]	0.07 [− 0.05, 0.18]
Median [min, max]	2.00 [0.00, 4.00]	2.00 [0.00, 4.00]		

Sixth, we explored the associations between the predictors within each group to determine which interactions should be considered when analysing the association with the dependent variables. This exploration showed that 22 pairs of categorical predictors showed statistically significant associations (p < 0.01) in one or both groups. Also, there are 14 statistically significant correlations (p < 0.01) between numeric dependent variables in one or both groups. The correlation between age and financial dependency is the strongest at -0.38 with a medium effect size in non-students, all the other effect sizes are small or very small even though they are statistically significant. Finally, there are 21 statistically significant relationships between a categorical predictor and a numeric predictor at p < 0.01.

Seventh, following those explorations of associations of the predictors within groups, we conducted several analyses to determine the possible associations between any of the predictors and any of the dependent variables within the groups. Six categorical predictors have an association with at least one dependent variable in at least one of the two groups significant at p < 0.01. These variables are gender, housing, taking care of older adults, having a chronic disease, MH diagnosis and having MH medication. Seven numerical predictors have an association with at least one dependent variable in at least one of the two groups significant at p < 0.01. These variables are age, financial dependency, financial distress, hours spent in social networks, having a constant routine, average exercising days per week and average sleeping hours per day. However, none of these relationships has an effect size that is more than small.

### Interaction between group and other predictors association and dependent variables

Eighth, we tested the relationship of all ten categorical variables showing significant associations with group for interactions with group against each of the seven dependent variables. Of the total 70 possible interactions considered, none were statistically significant interactions (p < 0.01). We followed the same procedure with the four numeric variables that presented significant differences between groups. In total 28 interactions were tested and only two showed statistically significant interactions (p < 0.01) with group on any of the dependent variables. The number of sleeping hours per day showed significant interactions with CORE-OM total score, CORE non-risk score. However, although statistically significant the effect sizes of all these interactions were very small.

### Distributions of the dependent variables

Finally, we include the description of the distributions of the dependent variables for referential purpose for emerging adults living in Quito. None of the variables showed a near fit to Gaussian distributions so the mean and SD are imperfect summary statistics for the empirical distributions making empirical percentiles more useful. However, the WGO ratings have only seven levels and there were only 25 observed scores for the EQ index and, as expected, just over 50% of the sample scored zero on the CORE-OM risk score making percentiles only of limited value for those measures. Full percentiles for the CORE-OM total score are given here in Table 5 and comprehensive detail on the distributions of all scores are in the Supplementary Materials.

**Table 5:** Referential centiles for the CORE-OM total score.

Group	Gender	5th	10th	20th	30th	40th	50th	60th	70th	80th	90th	95th
All	Both	0.26 [0.24, 0.28]	0.35 [0.32, 0.38]	0.50 [0.47, 0.53]	0.65 [0.59, 0.71]	0.79 [0.76, 0.85]	0.97 [0.91, 1.03]	1.15 [1.09, 1.21]	1.35 [1.29, 1.38]	1.56 [1.50, 1.62]	1.88 [1.79, 1.97]	2.21 [2.07, 2.29]
All	Female	0.29 [0.26, 0.35]	0.41 [0.35, 0.44]	0.56 [0.53, 0.62]	0.74 [0.65, 0.79]	0.91 [0.84, 0.97]	1.06 [1.00, 1.15]	1.25 [1.18, 1.32]	1.41 [1.35, 1.47]	1.62 [1.54, 1.71]	1.97 [1.88, 2.10]	2.29 [2.21, 2.38]
All	Male	0.21 [0.15, 0.24]	0.29 [0.24, 0.35]	0.44 [0.38, 0.47]	0.56 [0.50, 0.59]	0.69 [0.62, 0.75]	0.82 [0.76, 0.90]	0.97 [0.91, 1.09]	1.21 [1.12, 1.29]	1.44 [1.32, 1.53]	1.74 [1.65, 1.84]	1.98 [1.85, 2.12]
NS	Both	0.24 [0.21, 0.26]	0.35 [0.29, 0.38]	0.47 [0.44, 0.53]	0.59 [0.56, 0.62]	0.74 [0.65, 0.79]	0.88 [0.85, 0.94]	1.06 [0.98, 1.15]	1.26 [1.21, 1.35]	1.47 [1.41, 1.55]	1.79 [1.71, 1.88]	2.03 [1.94, 2.21]
NS	Female	0.26 [0.24, 0.34]	0.38 [0.32, 0.44]	0.53 [0.47, 0.59]	0.62 [0.59, 0.74]	0.85 [0.74, 0.94]	1.00 [0.91, 1.06]	1.15 [1.06, 1.26]	1.35 [1.26, 1.44]	1.53 [1.44, 1.65]	1.88 [1.78, 2.03]	2.18 [1.98, 2.38]
NS	Male	0.24 [0.13, 0.26]	0.29 [0.24, 0.35]	0.44 [0.38, 0.47]	0.53 [0.47, 0.59]	0.65 [0.56, 0.71]	0.78 [0.71, 0.88]	0.91 [0.85, 1.03]	1.15 [1.00, 1.27]	1.38 [1.26, 1.47]	1.68 [1.53, 1.80]	1.88 [1.74, 2.08]
S	Both	0.26 [0.21, 0.32]	0.38 [0.32, 0.44]	0.56 [0.50, 0.62]	0.74 [0.68, 0.79]	0.88 [0.82, 0.97]	1.06 [1.00, 1.15]	1.24 [1.15, 1.32]	1.41 [1.35, 1.50]	1.65 [1.59, 1.74]	1.99 [1.85, 2.16]	2.29 [2.18, 2.38]
S	Female	0.32 [0.24, 0.38]	0.45 [0.35, 0.53]	0.65 [0.56, 0.74]	0.79 [0.74, 0.88]	1.00 [0.88, 1.06]	1.15 [1.06, 1.26]	1.32 [1.24, 1.38]	1.47 [1.38, 1.59]	1.68 [1.61, 1.80]	2.08 [1.92, 2.27]	2.35 [2.26, 2.53]
S	Male	0.19 [0.15, 0.27]	0.29 [0.21, 0.38]	0.47 [0.38, 0.53]	0.59 [0.50, 0.71]	0.76 [0.63, 0.85]	0.91 [0.79, 1.03]	1.09 [0.93, 1.22]	1.29 [1.13, 1.43]	1.53 [1.35, 1.74]	1.82 [1.74, 2.02]	2.06 [1.88, 2.33]

*NS* non-student, *S* student, columns give scores for the centile with 95% confidence intervals.

## Discussion

The primary aim of this paper is to compare two groups: students and non-students to start to reduce the neglect of the latter group in the literature on MH and QoL in emerging adulthood. Another key aim was to start to compensate for a deficit of such information from middle-income countries.

As will be the case in many countries, in Ecuador there is no population register on which to base randomised sampling of participants nor is there funding to support other large-scale attempts to reach non-students for participation. Recognising these realities, we opted to "branch" the sample from volunteering participants reached through one university list of students' email addresses and then to invite students to invite non-student friends, relatives or acquaintances to participate in the survey. Clearly, this will create samples with definite biases: not all students will participate and those who do will invite in non-students who are not likely to be representative of the general emerging adult, non-student population.

In light of this, our results start with a comparison, where possible, of our sample and the groups, with 2010 census data for this age group in Quito. The census data showed that 35% were studying (though not necessarily for an undergraduate degree) and 38% already had an undergraduate degree. So, as we know, students and those who have already gained degrees do represent a very large proportion of the population underlining the importance of knowing more about their MH and QoL. The comparison of our sample with the census data showed an over-representation of women in our sample (59% versus 51%) but also very marked differences in social status and parenting with a far lower proportion of our sample partnered (and divorced) than in the census data and far fewer parents in our samples. Generalisation from these findings to the entire Ecuadorean population of emerging adults must be very cautious.

We found poorer MH in the students than the non-students, although effect sizes were small (g from − 0.25 to − 0.24) and scores on risk did not show statistically significant group differences despite the large group sizes. These results are similar to those found in two studies conducted in France^16,17^ and the USA with a Latinx population^18^, but different to those found in some other studies conducted in developed countries^12–14^ where the MH of the students was better than non-students. However, those studies reported results after analysing large national surveys and clearly larger and more representative samples of the population. Moreover, those studies report on the prevalence of diagnoses, while our study treated MH as a continuous variable as we believe this provides a better understanding of MH in emerging adults than do mapping from measures to diagnoses.

One previous study in Ecuador^25^, before the coronavirus pandemic, also found that students present significantly higher levels of distress than the general population. Our data were collected between October 2020 and January 2021 when, due to COVID-19 restrictions in the country, online teaching had replaced face-to-face teaching for all the students of our sample and it is possible because of the teaching change, restrictions impacted more students than non-students. Similar results were found in other countries during different stages of the pandemic^16,34,35^ where the psychological impact was greater in students than in non-students. Online learning might cause distress and even more after being mandatory for almost a year^36^, however, other pressures experienced in the pandemic might cause distress for all emerging adults, such as less social interaction, pressures to accommodate to technology and unstable internet connections, common in less developed countries. More research is clearly needed to understand the post-pandemic scenario for MH of the emerging adults.

In relation to QoL, non-students presented better health-related QoL than students, but again the effect sizes were small (EQ-VAS g = 0.26 and EQ index g = 0.27). Usually, only college students’ samples have been used to assess the psychometric properties of the EQ assessment tools^37^ and to compare with groups of people diagnosed with specific health conditions^38^.

Comparing between groups was not our only objective, we also wanted to understand how other sociodemographic, lifestyle and mental help-seeking variables are related to MH and QoL, and how these might relate to group. When analysing the direct association between sociodemographic predictors and MH and QoL, we found several statistically significant associations though all effect sizes were small. We then explored how any associations between sociodemographic predictors and MH/QoL might confound group differences. The average number of sleeping hours per day showed significant associations with group, with a strong effect size. It also showed a statistically significant interaction with group for CORE-OM total score and non-risk items score, however, these interactions had very small effect sizes. It is essentially impossible to attribute causality to any of these relationships and that was not our aim. What the data do show is that differences between the groups on some sociodemographic predictor variables were statistically significant and had fairly strong effects, notably for social status, sleeping hours and parenting. By comparison, though there were statistically significant differences between groups in MH and QoL scores, these differences were small or very small. Interestingly, none of the predictor variables showed important confounding with group effects on MH and QoL.

We are particularly interested in life events as potential contributors to perceived MH and QoL and included two items, WGO+ and WGO−, assessing the perception of positive and negative events experienced in the previous two months. Neither showed statistically significant differences between groups. Our main interest in life events is in temporal fluctuations across the further six two monthly evaluation points of the study, and in whether they show any temporal association with MH and QoL, so finding no group differences at baseline to some extent simplifies the later analyses. Though rating events did not differ significantly between the groups, it remains possible that the nature of the reported events reported would differ and that will be the subject of later thematic analysis.

## Limitations

As noted above, generalisation from these findings must be very cautious. No true randomised sampling was possible and convenience sampling methods branching from volunteers in the student group were used. The sample is also from just one university and one located in the capital city, Quito. We hope to conduct replication and extension work with other sampling frames and locations. As with all volunteer sampling declining to participate will not be random and this will affect both groups as the non-student sample was reached by branching from the student participants which may well mean that the participating non-student sample may be more similar to the student sample than are other non-student emerging adult Ecuadorians. Unsurprisingly, these issues resulted in differences between the sample and data from the last census conducted in Ecuador (in 2010) as described above.

The main other limitations are related to the measures used. Limited socio-demographic data were collected to avoid high non-participation. This meant that many important aspects of family relationships, income and cohabitation were not collected. Similarly, to keep the repeated measures relatively quick to complete, the issue of stressors and life events, so important for emerging adults was covered using a partly participant-generated measure to avoid long checklist approaches. We hope that different approaches, including small n qualitative work, can start to complement our findings and expand our understanding of emerging adulthood in Ecuador and other LMIC countries.

## Conclusions

Despite the inevitable limitations on generalisability, this study provides the first valuable information about MH and QoL of emerging adults living in a developing country and about the differences between students and non-students. We provide information about sociodemographic, lifestyle and MH variables related to MH and QoL using a continuous score model to identify the levels of distress rather than reducing scores to the estimated prevalence of MH diagnoses. More research is needed to capture the MH and QoL of emerging adults not included in this study in Ecuador and in another middle- and low-income countries. Such work should include samples of those living in rural areas, with limited resources and with different opportunities for employment and training than those offered by studying at a university.

However, the findings of this baseline analysis will be of interest to public and private organizations concerned about the MH of emerging adults. Middle-income countries are growing and workforces are changing, studying at university is becoming more common in the life paths of inhabitants of these regions, creating a huge global need to understand these changes and the effects on MH and QoL of this evolving population. The development of these countries, as well as their entry into a globalized society, grants the opportunity to think about emerging adulthood as a common stage in the lifespan of the entire population. Society is evolving, as well as  the tasks and features of each life stage. Large-scale research on MH of emerging adults living in low-and-middle income countries is still needed to generate psychosocial interventions adjusted to their needs and the existing resources.

## Supplementary Information

Supplementary Information.

## Data Availability

The data and code used to perform these analyses is available at Open Science Framework: osf.io/4fsyn.
