# Supplementary material for: A comparison of mental health of student and not student emerging adults living in Ecuador
Source: Sci Rep. 2023 Jan 27;13:1487. doi: 10.1038/s41598-023-27695-0 (PMC9880921; doi:10.1038/s41598-023-27695-0)
Supplement: Supplementary file 1 — Supplementary Information. [file 41598_2023_27695_MOESM1_ESM.pdf]

A comparison of mental health of student and not student emerging adults living in Ecuador

(Supplementarymaterial:code)

Clara Paz & Chris Evans

2021

Contents

Overview **3**

Flow of participants **4**

[1] Comparison with census data **4**

Highest educational attainment . . . 4

Gender . . . 5

Social status . . . 5

Number of children . . . 7

Summarising the comparison with the census data . . . 9

[2] Psychometrics of measures **9**

Internal consistency . . . 9

CORE . . . 9

EQ5D3L . . . 10

PCA . . . 10

CORE . . . 10

Non-students . . . 10

Students . . . 12

Both groups . . . 14

EQ5D3L . . . 16

Non-students . . . 16

Students . . . 18

Both groups . . . 19

CORE and EQ . . . 20

Non-students . . . 20

Students . . . 22
Both groups . . . 24
Score correlations . . . 25
Table 1: score intercorrelations . . . 25

[4] Simple contrasts between groups **27**
Table 3: categorical variables . . . 27
[3] (Table 2): continuous variables . . . 32
Table 4: continuous dependent variables . . . 33

Associations and interactions of variables **33**
[3] Are there differences between the two groups (E/NE) at p < .01? . . . 34
Dependent variables . . . 34
CORE scores . . . 34
EQ scores . . . 35
[4] Correlations between dependent variables . . . 38
EQ-Index and CORE . . . 38
EQ-VAS and CORE . . . 39
Correlations with the WGO . . . 39
WGO positive . . . 39
WGO positive and CORE total . . . 40
WGO positive and EQ . . . 41
WGO negative . . . 41
WGO negative and CORE total . . . 42
WGO negative and EQ . . . 43

[9] Distributions **43**
Summarising relationships between dependents and group . . . 44
Relationships between categorical predictors and group . . . 44
Relationships between numeric predictors and group . . . 46

Within group relationships betweeen numeric predictor variables and dependent variables **47**
COREnr . . . 60
CORErisk . . . 61
COREtotal . . . 62
EQ.index . . . 63
EQ.VAS . . . 64
WGO.negative . . . 65
WGO.positive . . . 66

Within group relationships betweeen predictors **66**

Cumulative summary of what we have so far **70**

Do categorical predictors associated with group affect how group affects scores on dependent variables? 71

Do numeric predictors correlated with group affect how group impacts on dependent variables? . . 72

(9) Distributions **73**

CORE-OM total score 74

CORE-OM non-risk score 76

CORE-OM risk score 78

EQ index score 80

EQ VAS 82

WGO negative 84

WGO positive 87

Conventional summary statistics 88

Centiles for scores 90

Centiles for CORE-OM total score 90

Centiles for CORE-OM non-risk score 93

Centiles for CORE-OM risk score 96

Centiles for EQ index score 98

Centiles for EQ VAS 101

Centiles for WGO negative 104

Centiles for WGO positive 107

Overview

This Rmarkdown file provides the analyses reported in the paper (full title " "). In the methods section of the paper we give the steps in the analyses as follows.

```
[1] The analyses started with a comparison with census data.
[2] Psychometric exploration of the multi-item dependent variables (CORE-OM, and EQ) included internal c
[3] Simple effects of group on dependent variables.  This was then put in context through the next four
[4] Summary descriptions of the predictors (sociodemographic, lifestyle and mental health variables).
[5] Exploration of group differences between the groups (students vs. non-students).
[6] Evaluation of associations between all predictors within each group.
[7] Exploration of univariate associations between each predictor and each dependent variable within the
[8] Each of the predictors which showed significant relationships with the dependent variables were incl
[9] Finally, we describe distributions of the dependent variables to provide referential data for emergi
```

Where sections of this file map to that sequence, the corresponding number is given in the section heading.

Flow of participants

We start with 1354 participants agreeing to participate and meeting inclusion criteria, however, only 1076 went on to complete the measures at baseline. Does this non-participation relate to group?

complete	nonstudents	students
complete	54.8% (590)	45.2% (486)
nomeasures	57.9% (161)	42.1% (117)

So no statistically significant difference between groups in non-participation: χ^2(1, n = 1354) = 0.73, p = .393. OK.

[1] Comparison with census data

We compared our sample, and the separate groups ("groups" in the paper) with referential data from the 2020 census. The referential data are for Quito for young people aged 18 to 29.

We know that there are essentially no truly unbiased population samples or even sampling frames that will result in truly unbiased samples (plausible selective non-response inclinations lead to opting out that is not unbiased even when the sampling frame is unbiased in principle).

We know that ours is definitely not an unbiased sample: that was made impossible both by the absence of population registers that can be used for research purposes, and because funding for the study was minimal. Hence the design was opt in volunteering across students in one private university in Quito and then branching per opting in participant suggesting non-student people they knew [... should fill in precise details here some time ...]. There will be tiny non-response bias even in the census and clearly the population of Quito will have changed between 2010 and 2021.

Hence the aim, was not to test for statistically significant differences between the demographics of the groups and the census data but to have a clear description of the extent to which the demographics of the two groups differed from that of the census data: from the Quito resident 18 to 29 year olds from the 2020 census.

The analyses clearly had to be limited to the variables present in the survey and in the census and data from the census about education only provide a context rather than even an exploratory descriptive comparison as the survey groups are selected on education.

Highest educational attainment

This was answered by 495,197 i.e. 97.3% of the census responders.

level	n	perc
Kindergarden	591	0.1
Primary	67505	13.3
Secondary	125398	24.6
Elementary	13061	2.6
High School	69750	13.7
Professional training	11632	2.3
Undergraduate	194658	38.3
Postgraduate	6821	1.3

It can be seen that the modal highest level achieved was "Undergraduate", n = 194,658, 38.3% of those responding.

Gender

gender	n	percent
female	635	59.01%
male	437	40.61%
other	4	0.37%

The referential data are (copying and pasting from the Excel).

```
Male    Female  Total
249305  259418  508723
49.01%  50.99%  100.00%
```

So remove "other" from our data.

gender	n	percent
female	635	59.24%
male	437	40.76%

So that gives us χ^2(1, n = 509795) = 28.74, p < .001 which is pretty uninformative but the OR is more informative: 1.4 [1.24, 1.58]. So, as we expected, females over-represented in our sample. Perhaps not as much as I'd expected.

Social status

Here is the breakdown for our overall sample.

civil.status	n	percent
divorced	6	0.6%
married	42	3.9%
single	1027	95.4%
widower	1	0.1%

And by group.

civil.status	nonstudents	students
divorced	0.8% (5)	0.2% (1)
married	5.6% (33)	1.9% (9)
single	93.4% (551)	97.9% (476)
widower	0.2% (1)	0.0% (0)

As we knew, that difference is statistically significant: χ^2(3, n = 1076) = 12.93, p = .005 with OR 1.4 [1.24, 1.58].

The referential data are as follows.

name	value	check	perc
Married	117959	117959	23.19
living wiht a partner	94704	212663	18.62
Separated	12208	224871	2.4
Divorced	3611	228482	0.7098
Willowed	843	229325	0.1657
Single	279398	508723	54.92
Total	508723	NA	100

OK, need to do some data munging here to get some alignment between our categories and the census categories.

I think the values that can be compared most sensibly between our data and the referential data, given the rarity of divorced status in our data are married, single. Here are the census data.

civil.status	n	perc
married	212663	41.8
single	296060	58.2

So how do the non-students compare with that?

civil.status	n	perc
married	33	5.6
single	552	93.6
NA	5	0.8

So for the non-students *versus* the referential for status values of we have χ^2(2, n = 721976) = 674.03, $p <$ .001. Clearly a huge difference, the odds ratio is 0.08 [0.06, 0.12].

Now the students.

civil.status	n	perc
married	9	1.9
single	476	97.9
NA	1	0.2

And for the students *versus* the referential for status values of we have χ^2(2, n = 721872) = 649.92, $p <$ .001: even bigger difference, OR: 0.03 [0.01, 0.05].

And our total sample.

civil.status	n	perc
married	42	3.9
single	1028	95.5
NA	6	0.6

χ^2(2, n = 721872) = 649.92, $p <$ .001. OR: 0.08 [0.06, 0.12].

Number of children

Here's our breakdown in the women.

number.kids	n	percent
0	579	91.18%
1	48	7.56%
2	8	1.26%

Broken down by group.

number.kids	nonstudents	students
0	85.67% (275)	96.82% (304)
1	12.15% (39)	2.87% (9)
2	2.18% (7)	0.32% (1)

nKids2	nonstudents	students
none	85.67% (275)	96.82% (304)
one or two	14.33% (46)	3.18% (10)

χ^2(1, n = 635) = 23.15, p < .001, OR 0.2 [0.09, 0.39]. As we can see, a highly statistically significant relationship between having kids or not and group. (We knew this already!)

Here are the referential data.

nKids	n	nKids2	check	nTot	nOK	percTot	percOK	LCL	UCL
0	122844	0	122844	259418	252441	47.35	48.66	0.4847	0.4886
1	70253	1	193097	259418	252441	27.08	27.83	0.2765	0.28
2	40811	2	233908	259418	252441	15.73	16.17	0.1602	0.1631
3	12872	3	246780	259418	252441	4.96	5.1	0.05014	0.05186
4	3693	4	250473	259418	252441	1.42	1.46	0.01417	0.0151
5	862	5	251335	259418	252441	0.33	0.34	0.003195	0.00365
6	207	6	251542	259418	252441	0.08	0.08	0.0007157	0.0009395
7	613	7	252155	259418	252441	0.24	0.24	0.002244	0.002628
8	57	8	252212	259418	252441	0.02	0.02	0.0001743	0.0002925
9	183	9	252395	259418	252441	0.07	0.07	0.0006273	0.0008378
10	8	10	252403	259418	252441	0	0	1.606e-05	6.254e-05
11	22	11	252425	259418	252441	0.01	0.01	5.756e-05	0.000132
12	16	12	252441	259418	252441	0.01	0.01	3.902e-05	0.000103
No response	6597	NA	259038	259418	252441	2.54	NA	NA	NA
Unknown	380	NA	259418	259418	252441	0.15	NA	NA	NA
Total	259418	NA	NA	259418	252441	100	NA	NA	NA

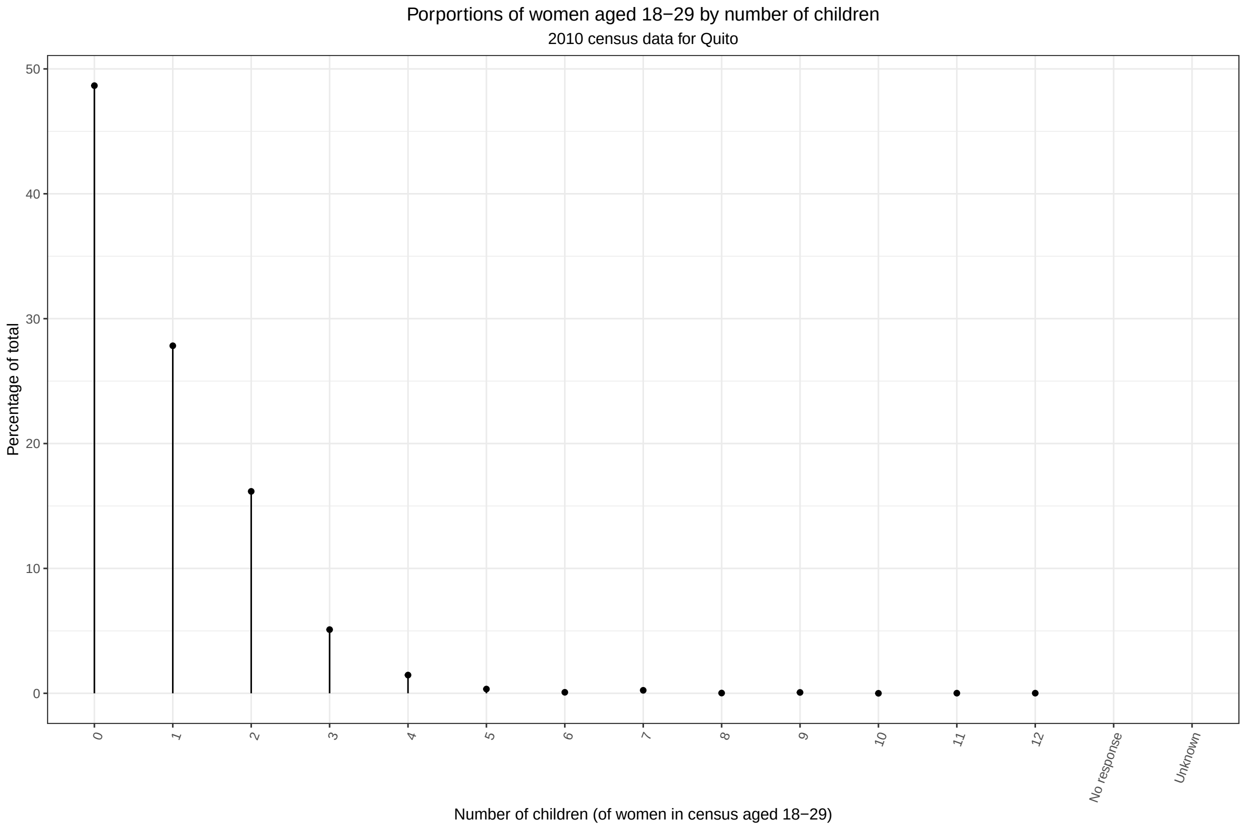

So the number of children ranged from zero to 15 with mean 0.862 and SD 1.097 and our sample is completely different from the number of children declared by the women. It's so different that this next is hardly worth doing but this next rather silly plot shows the proportions of the women in the total sample, and in each group, with one or more children with the horizontal reference line from the census data way above all three of those rates.

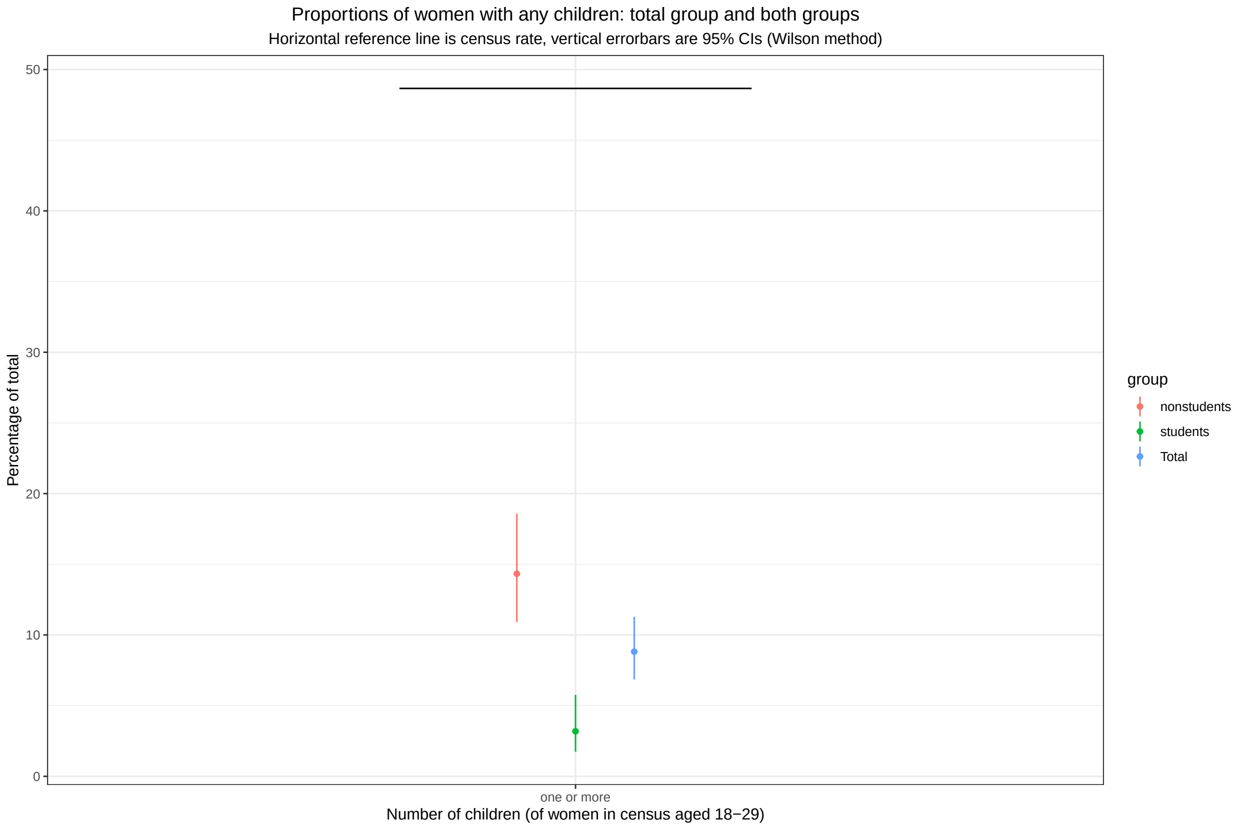

In our entire sample 91.18% did not have children, 95% CI 88.72% to 93.15%, dramatically different from 51.34% for the census data. $\chi^2(1, \text{n} = 253076) = 400.95$, $p < .001$, OR: 9.77 [7.5, 13.01].

Summarising the comparison with the census data

- Gender: we knew we had fewer men and more women. The census has as binary gender classification but our tiny number (4) isn't the explanation for any under-representation of men in the sample.
- Number of children: the census only gives this for women. The census rate of having one or more children for women is way above that of the women in either of our groups or, of course, the total sample. Whether our student data would be closer to the UDLA women students' rate of having children is another matter.
- There is a huge difference between the social status of both our groups (and the total sample) and the referential data: our participants have way fewer married/partnered and way more single.

[2] Psychometrics of measures

Internal consistency

CORE

group	obsAlpha	LCLAlpha	UCLAlpha	omega
nonstudents	0.9357	0.9277	0.9424	0.9462
students	0.9396	0.9315	0.9466	0.9476

EQ5D3L

group	obsAlpha	LCLAlpha	UCLAlpha	omega
nonstudents	0.498	0.4241	0.5605	0.5841
students	0.4105	0.3057	0.5033	0.5896

PCA

CORE

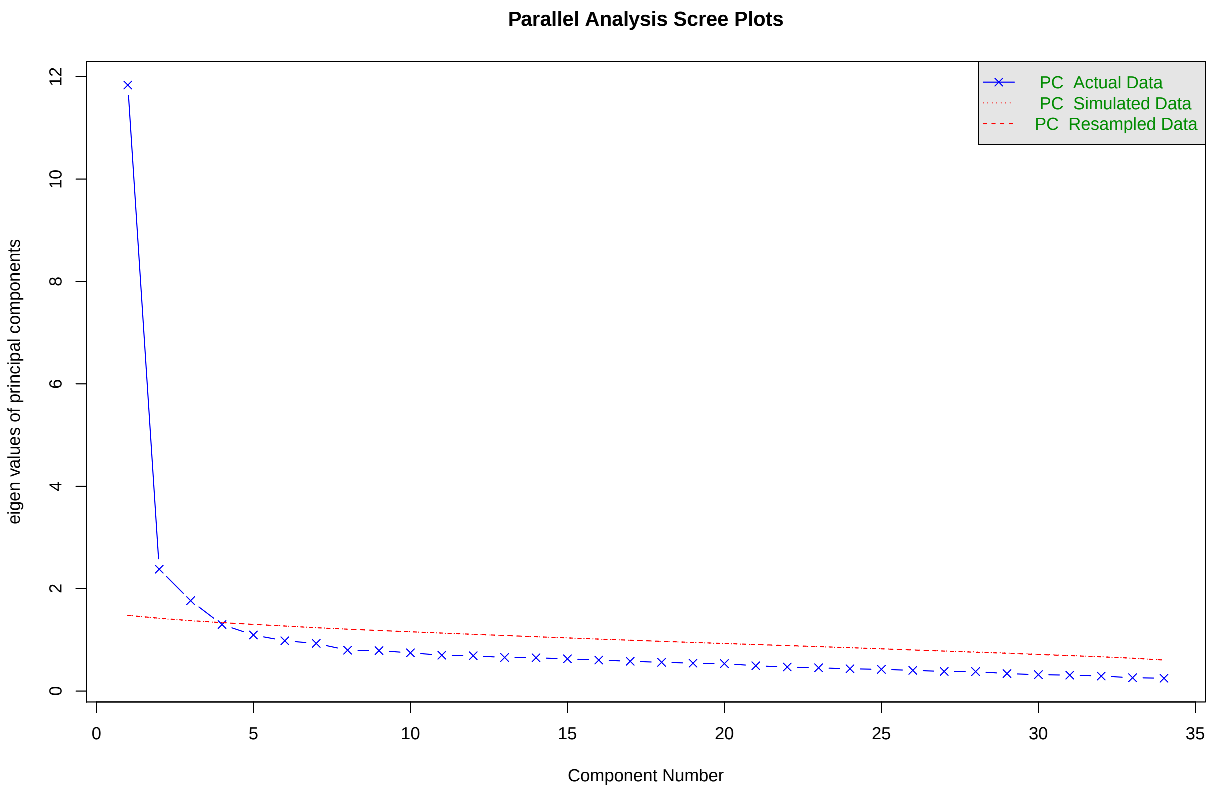

Non-students

```
## Parallel analysis suggests that the number of factors =  NA  and the number of components =  3
```

```
##
## Loadings:
##         RC1   RC2   RC3
## CORE01n  0.66
## CORE02n  0.83
## CORE05n  0.59
## CORE08n  0.54
## CORE10n  0.62
## CORE11n  0.63
## CORE13n  0.63
## CORE14n  0.85
## CORE15n  0.60
```

```
## CORE17n  0.76
## CORE18n  0.52
## CORE20n  0.67
## CORE23n  0.69
## CORE25n  0.70
## CORE26n  0.58
## CORE27n  0.52
## CORE28n  0.64
## CORE29n  0.76
## CORE30n  0.68
## CORE33n  0.52
## CORE03p        0.53
## CORE04p        0.57
## CORE07p        0.67
## CORE12p        0.73
## CORE19p        0.58
## CORE21p        0.69
## CORE31p        0.72
## CORE32p        0.82
## CORE09r              0.75
## CORE16r              0.83
## CORE22r              0.62
## CORE24r              0.56
## CORE34r              0.69
## CORE06r              0.41
##
##                  RC1  RC2  RC3
## SS loadings    8.97 3.85 2.75
## Proportion Var 0.26 0.11 0.08
## Cumulative Var 0.26 0.38 0.46
```

```
##                             RC1       RC2        RC3
## SS loadings           9.1944682 3.9844351 2.80295890
## Proportion Var        0.2704255 0.1171893 0.08243997
## Cumulative Var        0.2704255 0.3876148 0.47005477
## Proportion Explained  0.5753064 0.2493098 0.17538375
## Cumulative Proportion 0.5753064 0.8246163 1.00000000
```

The number of adequate components is 3 and the structure is clean (Component1 = positive worded items, Component 2 = Negative worded items and Component 3 = risk items)

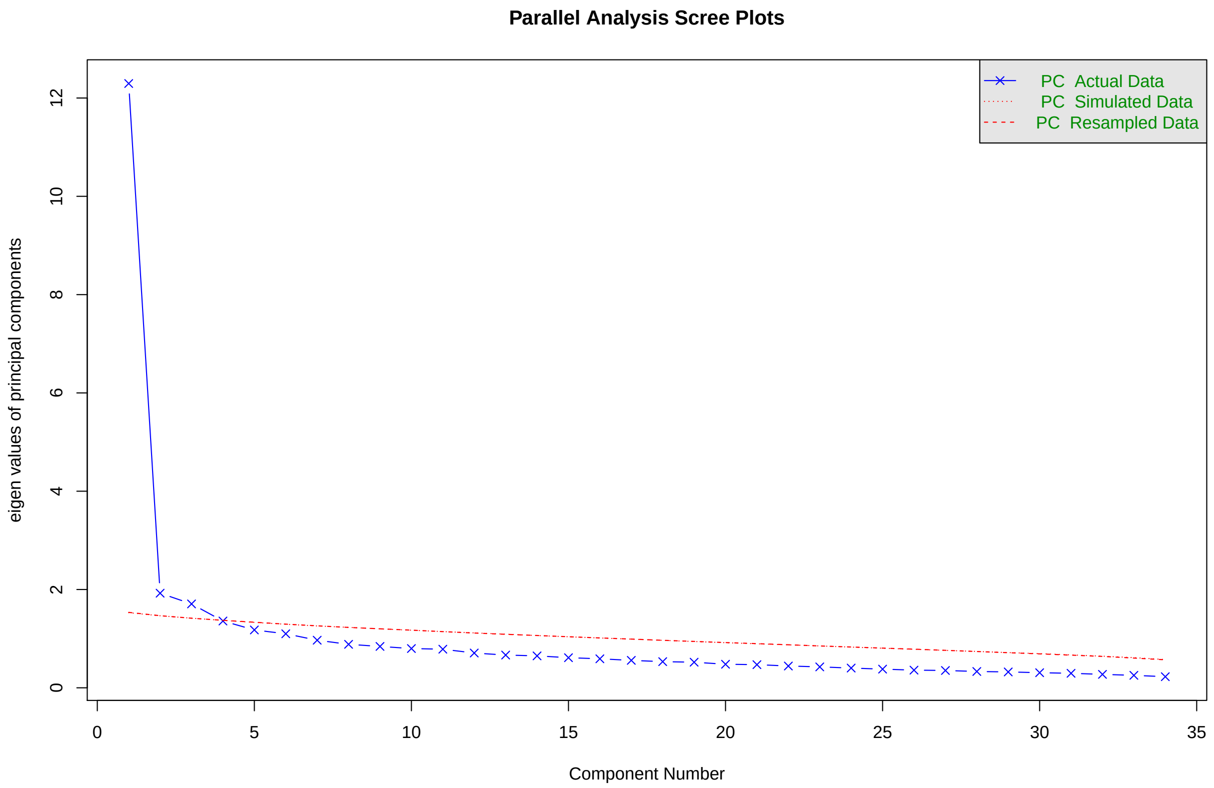

Students

```
## Parallel analysis suggests that the number of factors =  NA  and the number of components =  3

##
## Loadings:
##          RC1   RC3   RC2
## CORE01n  0.59
## CORE02n  0.85
## CORE05n  0.70
## CORE08n  0.66
## CORE10n  0.50
## CORE11n  0.68
## CORE13n  0.56
## CORE14n  0.74
## CORE15n  0.65
## CORE17n  0.86
## CORE20n  0.78
## CORE23n  0.63
## CORE25n  0.61
## CORE28n  0.56
## CORE29n  0.72
## CORE30n  0.55
## CORE33n  0.52
## CORE03p        0.62
## CORE12p        0.67
## CORE19p -0.44  0.71
## CORE21p        0.73
```

```
## CORE31p        0.59
## CORE32p        0.62
## CORE09r              0.80
## CORE16r              0.90
## CORE24r              0.72
## CORE34r              0.61
## CORE04p  0.41  0.43
## CORE06r
## CORE07p        0.48
## CORE18n  0.40
## CORE22r
## CORE26n  0.46
## CORE27n  0.45
##
##                 RC1  RC3  RC2
## SS loadings    8.90 3.26 2.98
## Proportion Var 0.26 0.10 0.09
## Cumulative Var 0.26 0.36 0.45
```

```
##                              RC1       RC3       RC2
## SS loadings           9.3287848 3.5673708 3.0310490
## Proportion Var        0.2743760 0.1049227 0.0891485
## Cumulative Var        0.2743760 0.3792987 0.4684472
## Proportion Explained  0.5857139 0.2239797 0.1903064
## Cumulative Proportion 0.5857139 0.8096936 1.0000000
```

The adequate number of components is 3, Component 1 = include all the negative worded items and item 19 ("He sentido afecto o cariño por alguien") a positive worded item which loads negatively, Component 3 include all the positive worded items and Component 2 include 3 of the six items of risks, the two other risk items do not load in any component (item 6 "He sido violento físicamente hacia los demás" and 22 "he amenazado o intimidado a otra persona"), those are items related to hurt another person.

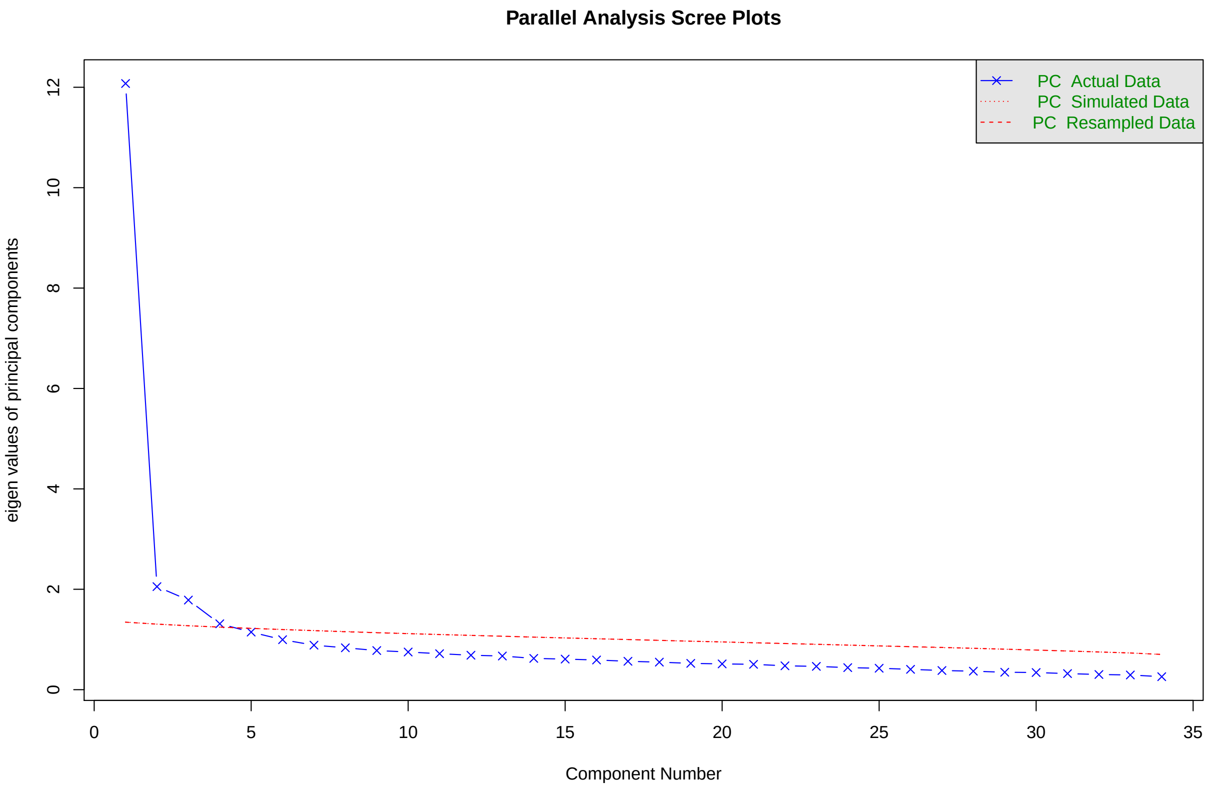

Both groups

```
## Parallel analysis suggests that the number of factors =  NA  and the number of components =  4

##
## Loadings:
##         RC1   RC2   RC3   RC4
## CORE01n  0.63
## CORE02n  0.89
## CORE05n  0.70
## CORE08n  0.63
## CORE10n  0.53
## CORE11n  0.67
## CORE13n  0.62
## CORE14n  0.83
## CORE15n  0.62
## CORE17n  0.81
## CORE20n  0.72
## CORE23n  0.65
## CORE25n  0.59
## CORE28n  0.60
## CORE29n  0.72
## CORE30n  0.60
## CORE04p        0.51
## CORE07p        0.58
## CORE12p        0.67
## CORE21p        0.64
## CORE31p        0.66
```

```
## CORE32p        0.74
## CORE09r              0.81
## CORE16r              0.88
## CORE24r              0.67
## CORE34r              0.67
## CORE03p                    0.64
## CORE19p                    0.74
## CORE06r                    0.49
## CORE18n  0.48
## CORE22r                    0.47
## CORE26n  0.46
## CORE27n  0.44
## CORE33n  0.43              0.40
##
##                  RC1  RC2  RC3  RC4
## SS loadings    8.76 2.95 2.60 2.12
## Proportion Var 0.26 0.09 0.08 0.06
## Cumulative Var 0.26 0.34 0.42 0.48
```

```
##                             RC1        RC2        RC3        RC4
## SS loadings           9.0496431 3.36602819 2.63615059 2.17403528
## Proportion Var        0.2661660 0.09900083 0.07753384 0.06394221
## Cumulative Var        0.2661660 0.36516680 0.44270064 0.50664286
## Proportion Explained  0.5253523 0.19540556 0.15303451 0.12620767
## Cumulative Proportion 0.5253523 0.72075782 0.87379233 1.00000000
```

For the whole sample 4 components seems to be adequate to extract. Component 1 = with all the negative worded items, Componet 2 = 6 out of 8 positive worded items, Component 3 = 4 of the 6 risk items, and Component 4 = the 2 negative worded missed items 3 "He sentido que tenía alguien en quien apoyarme cuando lo he necesitado" and 19 "He sentido cariño o afecto por alguien", the two missed risk items (22 and 6 which refer to hurt others) and one negative worded item 33 "Me he sentido humillada o avergonzada por otras personas". Component 4 collects items related to socialization and relationships with others.

EQ5D3L

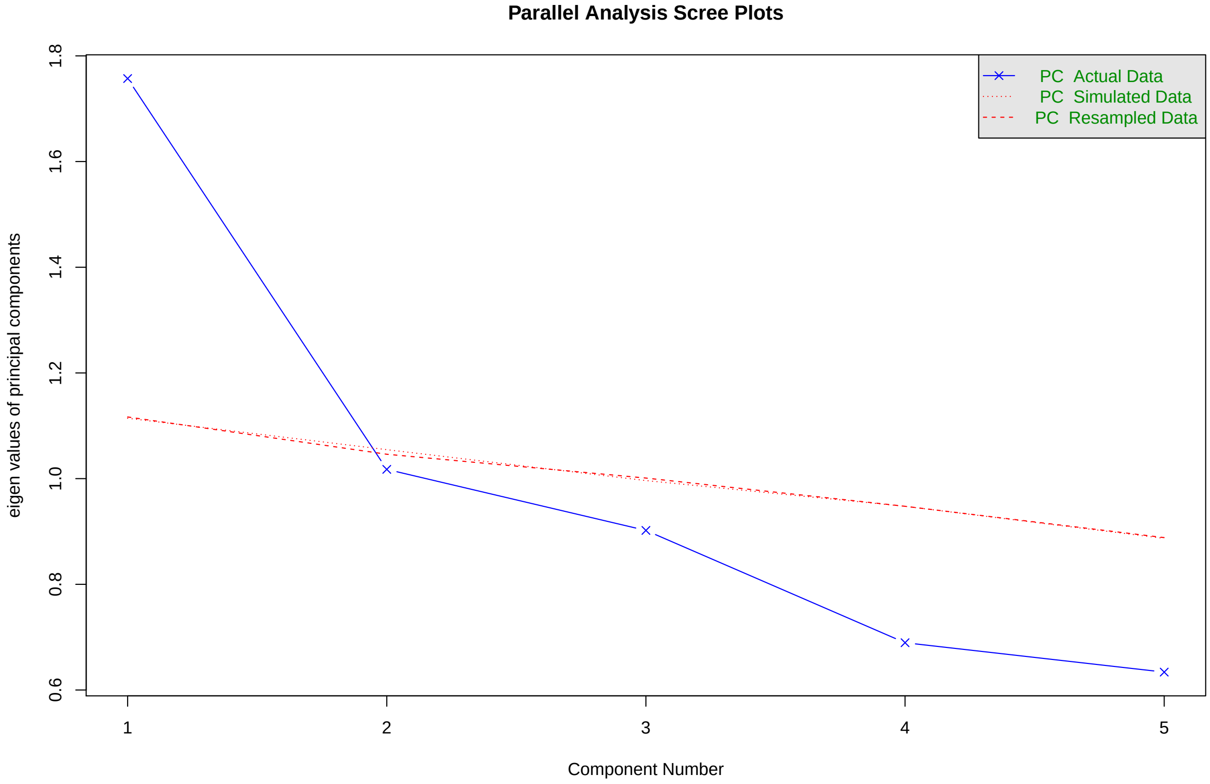

Non-students

```
## Parallel analysis suggests that the number of factors =  NA  and the number of components =  1
```

```
##
## Loadings:
## [1]  0.60  0.67  0.69 -0.65        0.43
##
##                   PC1
## SS loadings    1.96
## Proportion Var 0.33
```

```
##                        PC1
## SS loadings    1.9586545
## Proportion Var 0.3264424
```

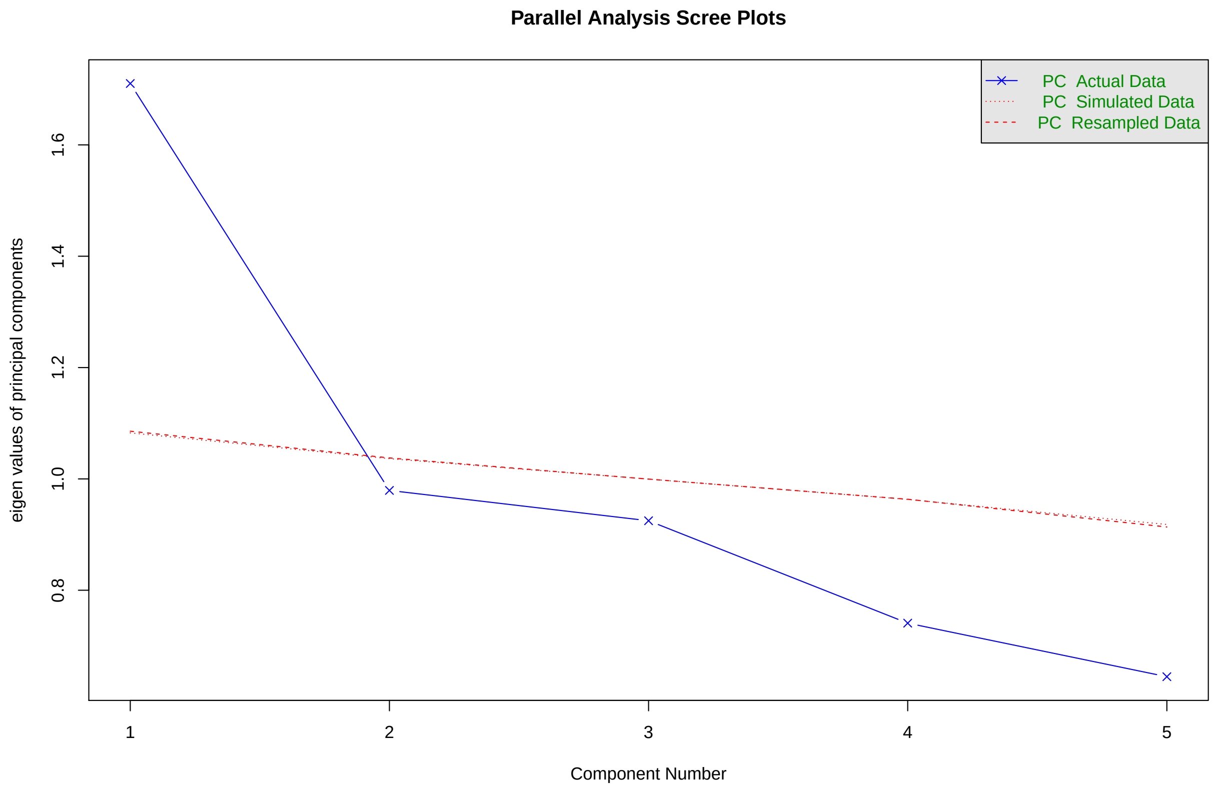


```
## Parallel analysis suggests that the number of factors =  NA  and the number of components =  1
```

```
##
## Loadings:
##  [1] 0.74 0.69 0.74 0.65 0.54 0.58 0.60 0.69 0.57 0.68 0.72 0.68 0.77 0.53 0.69
## [16] 0.78 0.64 0.61 0.58 0.69 0.67 0.65 0.62 0.61 0.55 0.58           0.41 0.42
## [31]      0.44
##
##                  PC1
## SS loadings    12.08
## Proportion Var  0.36
```

```
##                      PC1
## SS loadings    12.075031
## Proportion Var  0.355148
```

Due to the small number of items, one component is adequate to describe EQ5D3L scores

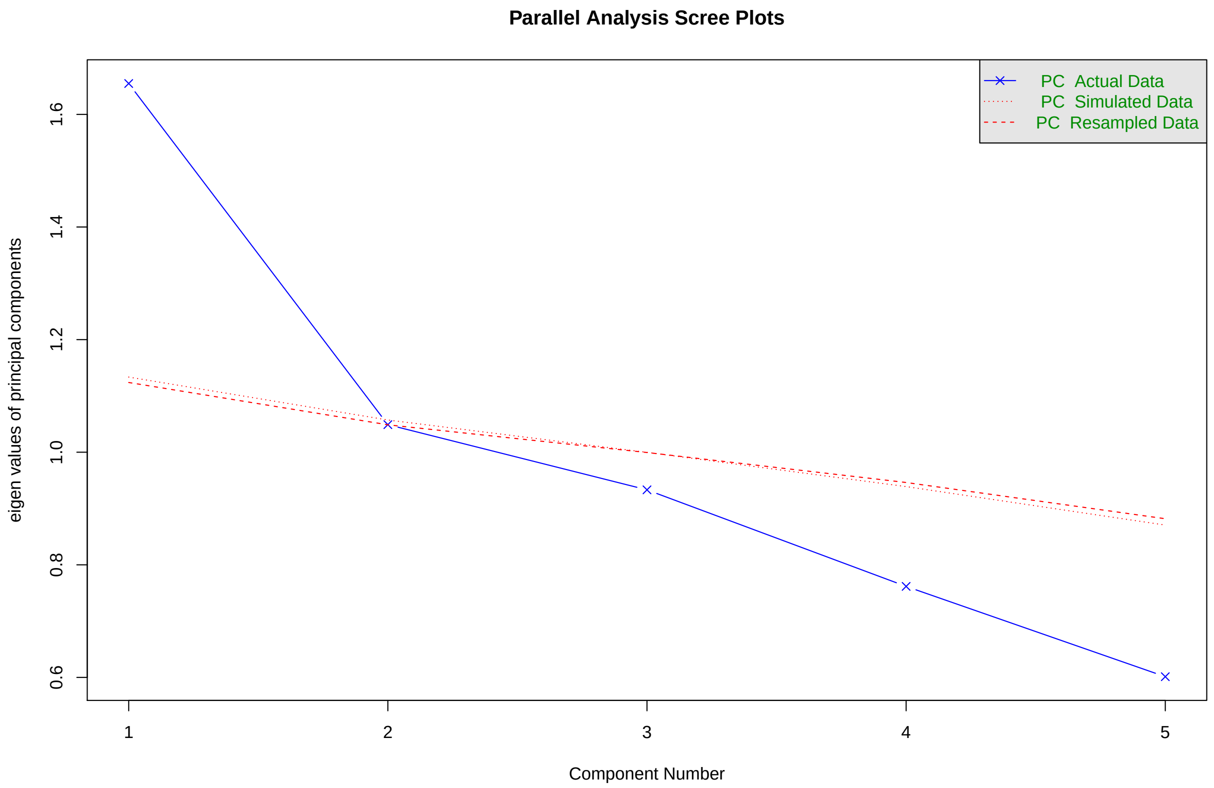

Students

```
## Parallel analysis suggests that the number of factors =  NA  and the number of components =  1
```

```
##
## Loadings:
## [1] 0.51 0.73 0.71      0.49
##
##                  PC1
## SS loadings     1.65
## Proportion Var 0.33
```

```
##                      PC1
## SS loadings    1.6548870
## Proportion Var 0.3309774
```

Due to the small number of items, one component is adequate to describe EQ5D3L scores

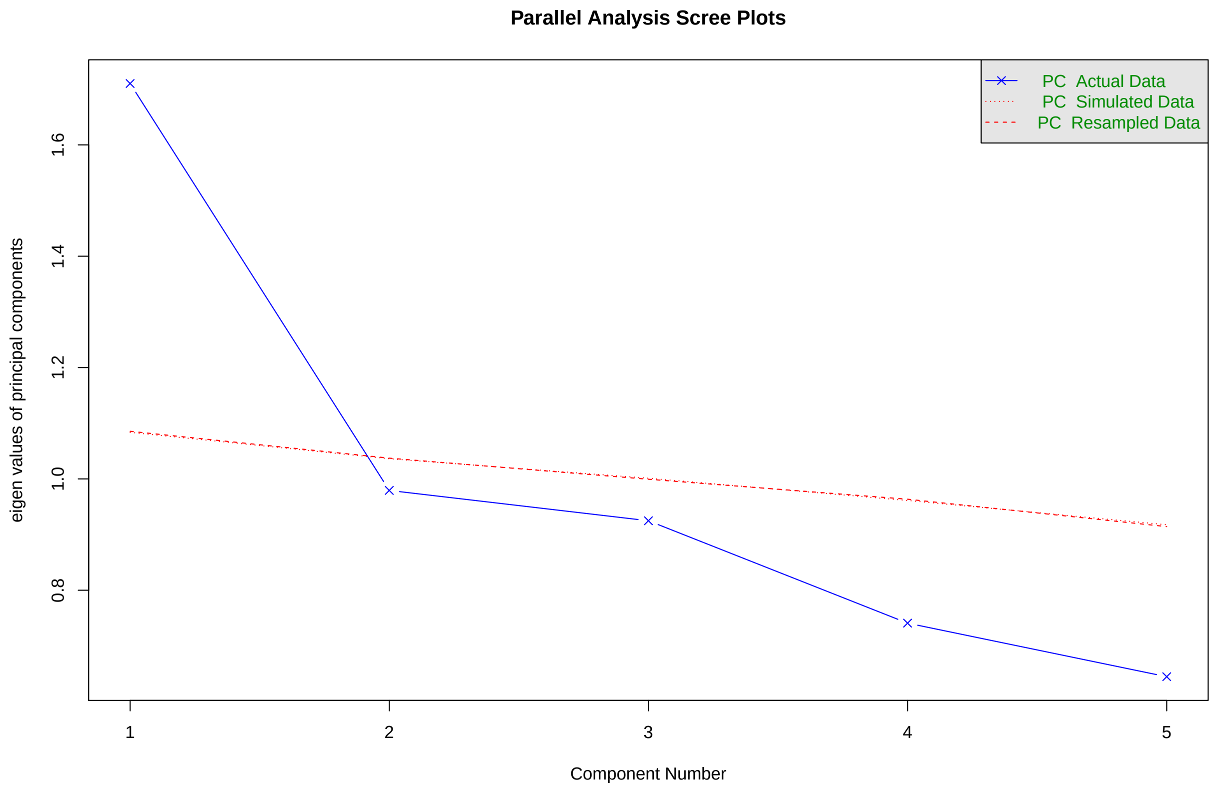

Both groups

```
## Parallel analysis suggests that the number of factors =  NA  and the number of components =  1
```

```
##
## Loadings:
##  [1] 0.74 0.69 0.74 0.65 0.54 0.58 0.60 0.69 0.57 0.68 0.72 0.68 0.77 0.53 0.69
## [16] 0.78 0.64 0.61 0.58 0.69 0.67 0.65 0.62 0.61 0.55 0.58           0.41 0.42
## [31]      0.44
##
##                  PC1
## SS loadings    12.08
## Proportion Var  0.36
```

```
##                      PC1
## SS loadings    12.075031
## Proportion Var  0.355148
```

Due to the small number of items, one component is adequate to describe EQ5D3L scores

CORE and EQ

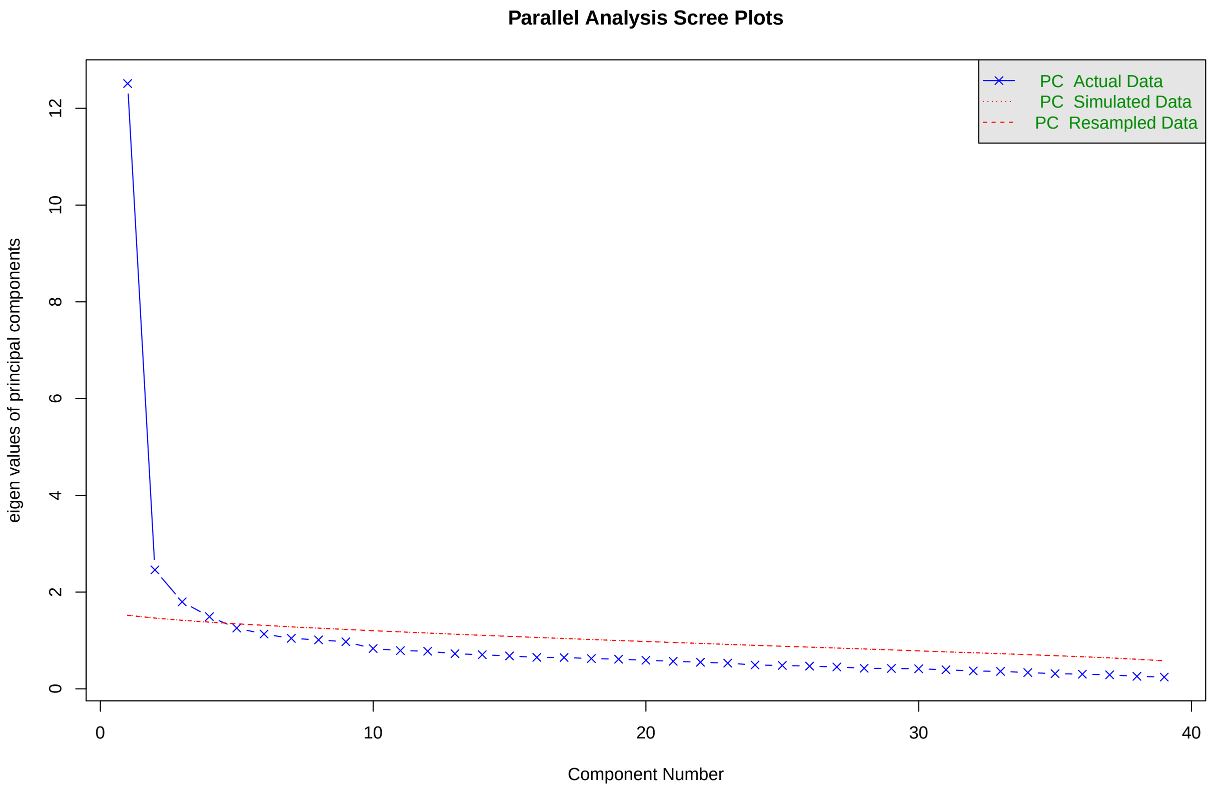

Non-students

```
## Parallel analysis suggests that the number of factors =  NA  and the number of components =  4
```

```
##
## Loadings:
##          RC1    RC2    RC3    RC4
## EQ.AD     0.60
## CORE01n  0.64
## CORE02n  0.81
## CORE05n  0.55
## CORE10n  0.59
## CORE11n  0.60
## CORE13n  0.64
## CORE14n  0.85
## CORE15n  0.62
## CORE17n  0.73
## CORE18n  0.54
## CORE20n  0.68
## CORE23n  0.70
## CORE25n  0.76
## CORE26n  0.60
## CORE27n  0.55
## CORE28n  0.67
## CORE29n  0.77
## CORE30n  0.68
```

```
## CORE33n  0.58
## CORE03p        0.51
## CORE04p        0.57
## CORE07p        0.67
## CORE12p        0.73
## CORE19p        0.52
## CORE21p        0.69
## CORE31p        0.72
## CORE32p        0.84
## CORE09r              0.73
## CORE16r              0.83
## CORE22r              0.61
## CORE24r              0.56
## CORE34r              0.69
## EQ.SC                      0.55
## EQ.PD                      0.60
## EQ.MO                      0.48
## EQ.UA
## CORE06r
## CORE08n  0.42              0.44
##
##                  RC1  RC2  RC3  RC4
## SS loadings    9.45 3.85 2.76 1.70
## Proportion Var 0.24 0.10 0.07 0.04
## Cumulative Var 0.24 0.34 0.41 0.46
```

```
##                              RC1       RC2       RC3        RC4
## SS loadings           9.7060421 3.9811679 2.7823574 1.79014420
## Proportion Var        0.2488729 0.1020812 0.0713425 0.04590113
## Cumulative Var        0.2488729 0.3509541 0.4222966 0.46819773
## Proportion Explained  0.5315551 0.2180302 0.1523769 0.09803792
## Cumulative Proportion 0.5315551 0.7495852 0.9019621 1.00000000
```

When we included the items from the CORE-OM and EQ5D3L, four components is the adequate solution. Component 1 = all negative worded items of the CORE-OM and the item of the EQ5D3L that refers to Depression and Anxiety, Component 3 = includes all the positive worded items, Component 3 = all the risk items and Component 4 = the other 4 items from the EQ.

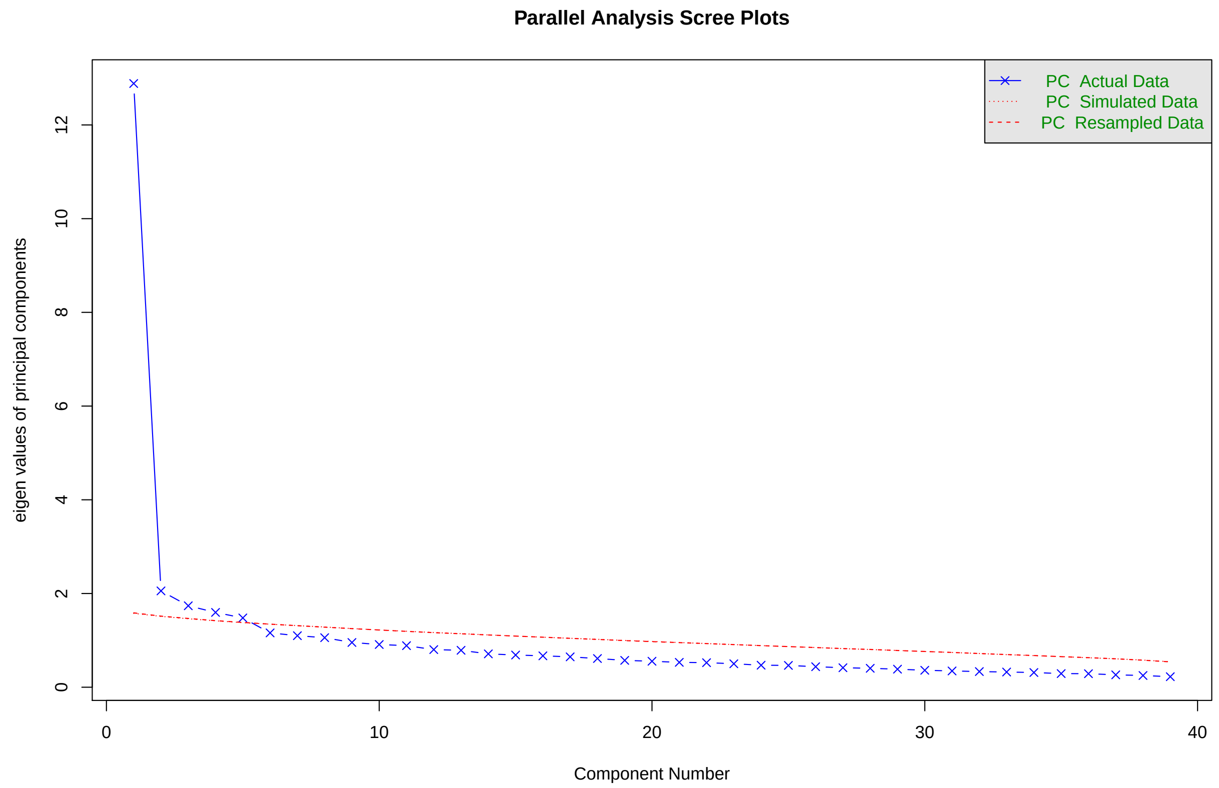

Students

```
## Parallel analysis suggests that the number of factors =  NA  and the number of components =  5

##
## Loadings:
##          RC1   RC3   RC2   RC5   RC4
## EQ.AD     0.63
## CORE01n  0.66
## CORE02n  0.84
## CORE05n  0.70
## CORE11n  0.64
## CORE13n  0.59
## CORE14n  0.81
## CORE15n  0.63
## CORE17n  0.84
## CORE20n  0.77
## CORE23n  0.67
## CORE25n  0.58
## CORE28n  0.58
## CORE29n  0.67
## CORE30n  0.56
## CORE12p        0.61
## CORE21p        0.69
## CORE31p        0.55
## CORE32p        0.59
## CORE09r              0.78
## CORE16r              0.85
```

```
## CORE24r              0.69
## CORE34r              0.62
## EQ.SC                      0.53  0.40
## CORE03p        0.49        0.57
## CORE19p -0.44  0.57        0.63
## EQ.UA                            0.70
## EQ.PD                            0.72
## CORE08n                          0.60
## EQ.MO
## CORE04p  0.50  0.41
## CORE06r
## CORE07p        0.45
## CORE10n  0.47
## CORE18n
## CORE22r
## CORE26n  0.41              0.41
## CORE27n  0.48
## CORE33n  0.42
##
##                  RC1  RC3  RC2  RC5  RC4
## SS loadings    9.26 2.73 2.66 2.01 1.85
## Proportion Var 0.24 0.07 0.07 0.05 0.05
## Cumulative Var 0.24 0.31 0.38 0.43 0.47
```

```
##                             RC1        RC3        RC2        RC5        RC4
## SS loadings           9.8667229 3.09866477 2.76088685 2.14278249 1.88403450
## Proportion Var        0.2529929 0.07945294 0.07079197 0.05494314 0.04830858
## Cumulative Var        0.2529929 0.33244584 0.40323781 0.45818095 0.50648953
## Proportion Explained  0.4995027 0.15686986 0.13976986 0.10847834 0.09537922
## Cumulative Proportion 0.4995027 0.65637258 0.79614244 0.90462078 1.00000000
```

For the student group 5 components is the adequate structure for the data. In Component 1 = all the CORE-OM negative worded items, except for 26 “He pensado que no tengo amigos” are included, item 19 (“He sentido afecto o cariño por alguien”) a positive worded item which loads negatively and item 4 “Me he sentido bien conmigo mismo” loasd postively and the AD item of the EQ. In Component 3 = the other positive itmes are included, Component 2 = 4 of the risk items are included and the other two (22 and 6) do not load in any item. In component 4 = the EQ items (SC, UA, PD) are included with the item 8 of the CORE-OM (“He tenido molestias, dolores y otros problemas físicos”), this is a component about physical difficulties, EQ.MO wich refers to mobility does not load in any component. In component 5 = items 3, “He sentido que tenía alguien en quien apoyarme cuando lo he necesitado” 19 “He sentido cariño o afecto por alguien”, 26 “He pensado que no tengo amigos” and the item of the EQ.SC (Self Care) load in this component.

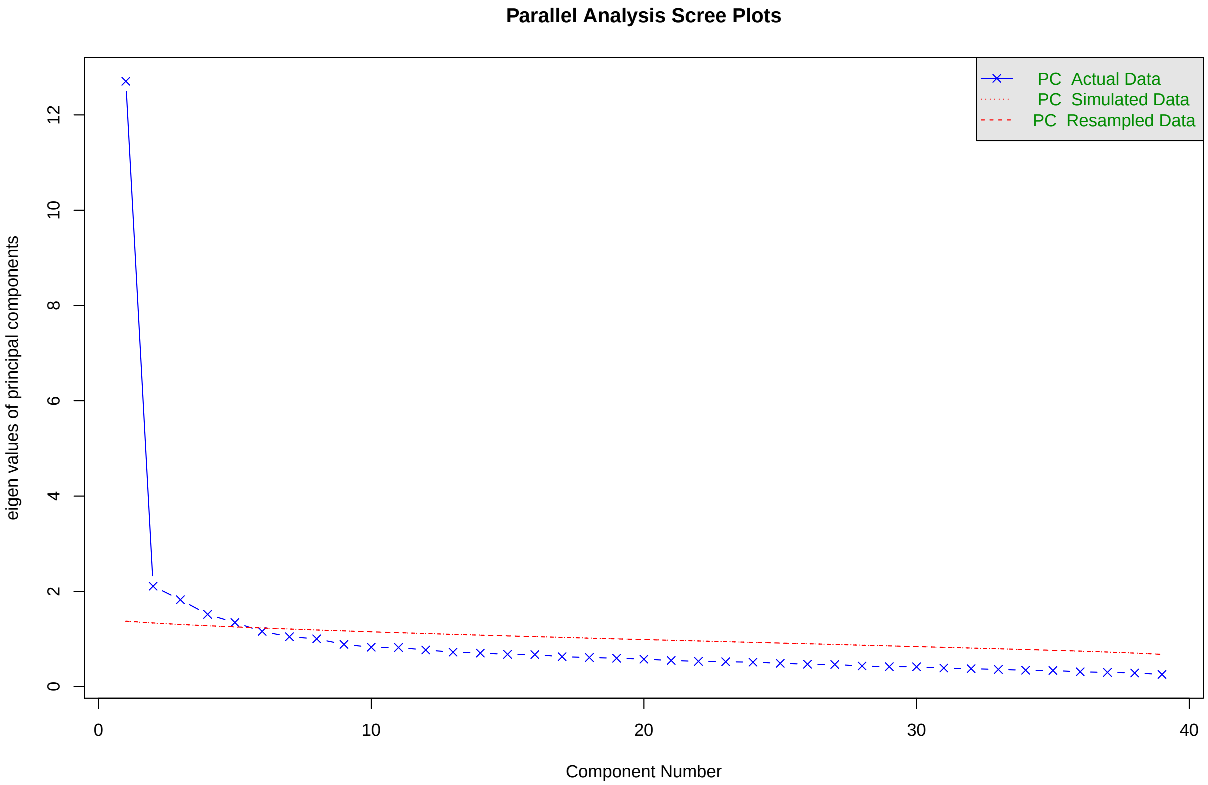

Both groups

```
## Parallel analysis suggests that the number of factors =  NA  and the number of components =  5

##
## Loadings:
##          RC1   RC2   RC3   RC5   RC4
## EQ.AD     0.61
## CORE01n  0.64
## CORE02n  0.82
## CORE05n  0.64
## CORE11n  0.59
## CORE13n  0.65
## CORE14n  0.86
## CORE15n  0.63
## CORE17n  0.77
## CORE20n  0.71
## CORE23n  0.68
## CORE25n  0.57
## CORE28n  0.62
## CORE29n  0.67
## CORE30n  0.59
## CORE04p  0.40  0.51
## CORE07p        0.58
## CORE12p        0.68
## CORE21p        0.67
## CORE31p        0.66
## CORE32p        0.74
```

```
## CORE09r              0.79
## CORE16r              0.85
## CORE24r              0.66
## CORE34r              0.65
## CORE03p                    0.53
## CORE19p        0.40        0.62
## EQ.SC                            0.50
## EQ.UA                            0.65
## EQ.PD                            0.73
## CORE08n                          0.57
## EQ.MO                            0.41
## CORE06r                    0.47
## CORE10n  0.47
## CORE18n  0.45
## CORE22r                    0.43
## CORE26n  0.42              0.46
## CORE27n  0.49
## CORE33n                    0.44
##
##                 RC1  RC2  RC3  RC5  RC4
## SS loadings    8.65 3.05 2.52 2.06 1.83
## Proportion Var 0.22 0.08 0.06 0.05 0.05
## Cumulative Var 0.22 0.30 0.36 0.42 0.46
```

```
##                             RC1        RC2        RC3        RC5        RC4
## SS loadings           9.2801115 3.42710881 2.60882240 2.30518635 1.88210227
## Proportion Var        0.2379516 0.08787458 0.06689288 0.05910734 0.04825903
## Cumulative Var        0.2379516 0.32582616 0.39271904 0.45182639 0.50008542
## Proportion Explained  0.4758219 0.17571915 0.13376291 0.11819449 0.09650158
## Cumulative Proportion 0.4758219 0.65154102 0.78530393 0.90349842 1.00000000
```

For both groups 5 components is an adequate structure. Component 1 = all the negative worded items of the CORE, EQ.AD and CORE 4 “Me he sentido bien conmigo mismo”. Component 2 = all the positive worded items except for item 3 “He sentido que tenía alguien en quien apoyarme cuando lo he necesitado”. Component 4 = all the items of the EQ except for AD and CORE8 which referes to physical issues. Component 5 = items 3, “He sentido que tenía alguien en quien apoyarme cuando lo he necesitado” 19 “He sentido cariño o afecto por alguien”, 26 “He pensado que no tengo amigos” 33 “Me he sentido humillado o avergonzado por otras personas” and the two risk items related to hurt another person 6 and 22.

Score correlations

Table 1: score intercorrelations

group	var1	COREtotal	COREerisk	COREnr	EQ.index	EQ.VAS	WGO.positive	WGO.negative
all	COREtotal							
all	COREerisk	+0.67 [+0.63, +0.70]						
all	COREnr	+1.00 [+0.99, +1.00]	+0.60 [+0.56, +0.64]					

group	var1	COREtotal	COREisk	COREnr	EQ.index	EQ.VAS	WGO.positive	WGO.negative
all	EQ.index	-0.58 [-0.62, -0.53]	-0.42 [-0.49, -0.35]	-0.57 [-0.61, -0.53]				
all	EQ.VAS	-0.55 [-0.60, -0.50]	-0.35 [-0.41, -0.28]	-0.55 [-0.60, -0.49]	+0.42 [+0.37, +0.48]			
all	WGO.positive	-0.49 [-0.54, -0.44]	-0.30 [-0.37, -0.24]	-0.50 [-0.55, -0.45]	+0.27 [+0.20, +0.33]	+0.26 [+0.20, +0.33]		
all	WGO.negative	-0.47 [-0.51, -0.42]	-0.25 [-0.30, -0.21]	-0.47 [-0.52, -0.42]	+0.30 [+0.25, +0.35]	+0.27 [+0.22, +0.33]	+0.29 [+0.24, +0.35]	
nonstudents	COREtotal							
nonstudents	COREisk	+0.67 [+0.61, +0.71]						
nonstudents	COREnr	+1.00 [+0.99, +1.00]	+0.59 [+0.53, +0.64]					
nonstudents	EQ.index	-0.58 [-0.64, -0.52]	-0.46 [-0.56, -0.33]	-0.57 [-0.63, -0.51]				
nonstudents	EQ.VAS	-0.53 [-0.61, -0.45]	-0.34 [-0.43, -0.24]	-0.53 [-0.60, -0.46]	+0.41 [+0.33, +0.49]			
nonstudents	WGO.positive	-0.50 [-0.57, -0.43]	-0.34 [-0.42, -0.25]	-0.49 [-0.56, -0.43]	+0.25 [+0.16, +0.35]	+0.27 [+0.18, +0.35]		
nonstudents	WGO.negative	-0.48 [-0.54, -0.42]	-0.21 [-0.28, -0.15]	-0.50 [-0.56, -0.44]	+0.33 [+0.27, +0.40]	+0.29 [+0.21, +0.37]	+0.27 [+0.18, +0.34]	
students	COREtotal							
students	COREisk	+0.68 [+0.63, +0.72]						
students	COREnr	+1.00 [+0.99, +1.00]	+0.61 [+0.56, +0.66]					
students	EQ.index	-0.56 [-0.63, -0.49]	-0.39 [-0.48, -0.29]	-0.56 [-0.63, -0.49]				
students	EQ.VAS	-0.55 [-0.63, -0.47]	-0.36 [-0.44, -0.27]	-0.55 [-0.63, -0.48]	+0.41 [+0.33, +0.49]			
students	WGO.positive	-0.48 [-0.56, -0.40]	-0.25 [-0.35, -0.16]	-0.49 [-0.57, -0.40]	+0.27 [+0.18, +0.36]	+0.25 [+0.15, +0.36]		
students	WGO.negative	-0.44 [-0.51, -0.37]	-0.30 [-0.37, -0.22]	-0.44 [-0.51, -0.37]	+0.27 [+0.18, +0.34]	+0.25 [+0.16, +0.34]	+0.32 [+0.24, +0.40]	

[4] Simple contrasts between groups

Table 3: categorical variables

This sequence of blocks gives all the values for Table 3. First the raw counts.

```
## # A tibble: 13 x 14
##    demogVar          nMiss observed1 observed2 observed3 observed4 observed5 observed6 observed7 obse
##    <ord>             <int>     <dbl>     <dbl>     <dbl>     <dbl>     <dbl>     <dbl>     <dbl>
##  1 gender                0       321       314       266       171         3         1        NA
##  2 employment            0        81         0       152         0         0       384         0
##  3 living                0        62        14         8         2        36        13         9
##  4 housing               0        49        26         5         9       386       370       150
##  5 civil.status          0         5         1        33         9       551       476         1
##  6 paternity             0       524       475        66        11        NA        NA        NA
##  7 minors                0       525       476        65        10        NA        NA        NA
##  8 infants               2       537       477        52         8        NA        NA        NA
##  9 care.older.adults     0       553       426        37        60        NA        NA        NA
## 10 main.caretaker        0       538       476        52        10        NA        NA        NA
## 11 mh.diagnosis          0       445       346       145       140        NA        NA        NA
## 12 mh.medication       791       113       111        32        29        NA        NA        NA
## 13 chronic.disease       0       566       445        24        41        NA        NA        NA
```

Now the percentages. These are raw cell percentages of the total.

```
## # A tibble: 13 x 14
##    demogVar          nMiss perc1  perc2  perc3  perc4  perc5  perc6  perc7  perc8 perc9  perc10 perc1
##    <ord>             <int> <chr>  <chr>  <chr>  <chr>  <chr>  <chr>  <chr>  <chr> <chr>  <chr>  <chr
##  1 gender                0 29.83% 29.18% 24.72% 15.89% 0.28%  0.09%  <NA>   <NA>  <NA>   <NA>   <NA>
##  2 employment            0 7.53%  0%     14.13% 0%     0%     35.69% 0%     9.48% 33.18% 0%     <NA>
##  3 living                0 5.76%  1.3%   0.74%  0.19%  3.35%  1.21%  0.84%  1.12% 41.08% 38.85% 3.07
##  4 housing               0 4.55%  2.42%  0.46%  0.84%  35.87% 34.39% 13.94% 7.53% <NA>   <NA>   <NA>
##  5 civil.status          0 0.46%  0.09%  3.07%  0.84%  51.21% 44.24% 0.09%  0%    <NA>   <NA>   <NA>
##  6 paternity             0 48.7%  44.14% 6.13%  1.02%  <NA>   <NA>   <NA>   <NA>  <NA>   <NA>   <NA>
##  7 minors                0 48.79% 44.24% 6.04%  0.93%  <NA>   <NA>   <NA>   <NA>  <NA>   <NA>   <NA>
##  8 infants               2 49.91% 44.33% 4.83%  0.74%  <NA>   <NA>   <NA>   <NA>  <NA>   <NA>   <NA>
##  9 care.older.adults     0 51.39% 39.59% 3.44%  5.58%  <NA>   <NA>   <NA>   <NA>  <NA>   <NA>   <NA>
## 10 main.caretaker        0 50%    44.24% 4.83%  0.93%  <NA>   <NA>   <NA>   <NA>  <NA>   <NA>   <NA>
## 11 mh.diagnosis          0 41.36% 32.16% 13.48% 13.01% <NA>   <NA>   <NA>   <NA>  <NA>   <NA>   <NA>
## 12 mh.medication       791 10.5%  10.32% 2.97%  2.7%   <NA>   <NA>   <NA>   <NA>  <NA>   <NA>   <NA>
## 13 chronic.disease       0 52.6%  41.36% 2.23%  3.81%  <NA>   <NA>   <NA>   <NA>  <NA>   <NA>   <NA>
```

Then I realised is sensibl to use column percentages. OK!

```
## # A tibble: 13 x 14
##    demogVar          nMiss perc1  perc2  perc3  perc4  perc5  perc6  perc7  perc8  perc9  perc10 per
##    <ord>             <int> <chr>  <chr>  <chr>  <chr>  <chr>  <chr>  <chr>  <chr>  <chr>  <chr>  <ch
##  1 gender                0 54.41% 64.61% 45.08% 35.19% 0.51%  0.21%  <NA>   <NA>   <NA>   <NA>   <NA
##  2 employment            0 13.73% 0%     25.76% 0%     0%     79.01% 0%     20.99% 60.51% 0%     <NA
##  3 living                0 10.51% 2.88%  1.36%  0.41%  6.1%   2.67%  1.53%  2.47%  74.92% 86.01% 5.59
##  4 housing               0 8.31%  5.35%  0.85%  1.85%  65.42% 76.13% 25.42% 16.67% <NA>   <NA>   <NA
##  5 civil.status          0 0.85%  0.21%  5.59%  1.85%  93.39% 97.94% 0.17%  0%     <NA>   <NA>   <NA
```

```
##  6 paternity            0 88.81% 97.74% 11.19% 2.26%  <NA>   <NA>   <NA>   <NA>   <NA>   <NA>   <NA
##  7 minors               0 88.98% 97.94% 11.02% 2.06%  <NA>   <NA>   <NA>   <NA>   <NA>   <NA>   <NA
##  8 infants              2 91.17% 98.35% 8.83%  1.65%  <NA>   <NA>   <NA>   <NA>   <NA>   <NA>   <NA
##  9 care.older.adults    0 93.73% 87.65% 6.27%  12.35% <NA>   <NA>   <NA>   <NA>   <NA>   <NA>   <NA
## 10 main.caretaker       0 91.19% 97.94% 8.81%  2.06%  <NA>   <NA>   <NA>   <NA>   <NA>   <NA>   <NA
## 11 mh.diagnosis         0 75.42% 71.19% 24.58% 28.81% <NA>   <NA>   <NA>   <NA>   <NA>   <NA>   <NA
## 12 mh.medication      791 77.93% 79.29% 22.07% 20.71% <NA>   <NA>   <NA>   <NA>   <NA>   <NA>   <NA
## 13 chronic.disease      0 95.93% 91.56% 4.07%  8.44%  <NA>   <NA>   <NA>   <NA>   <NA>   <NA>   <NA
```

Note: Using an external vector in selections is ambiguous. i Use `all_of(vecDemogVars)` instead of `vecDemogVars` to silence this message. i See https://tidyselect.r-lib.org/reference/faq-external-vector.html. This message is displayed once per session.

variable	value	nonstudents	students
gender	female	321 (54.41%)	314 (64.61%)
gender	male	266 (45.08%)	171 (35.19%)
gender	other	3 (0.51%)	1 (0.21%)
employment	working	357 (60.51%)	NA
employment	neither studying nor working	152 (25.76%)	NA
employment	home tasks	81 (13.73%)	NA
employment	studying	NA	384 (79.01%)
employment	studying and working	NA	102 (20.99%)
living	parents	442 (74.92%)	418 (86.01%)
living	couple or kids	62 (10.51%)	14 (2.88%)
living	living alone	36 (6.1%)	13 (2.67%)
living	relatives	33 (5.59%)	27 (5.56%)
living	other	9 (1.53%)	12 (2.47%)
living	friends	8 (1.36%)	2 (0.41%)
housing	own home	386 (65.42%)	370 (76.13%)
housing	renting	150 (25.42%)	81 (16.67%)
housing	borrowed	49 (8.31%)	26 (5.35%)
housing	other	5 (0.85%)	9 (1.85%)
civil.status	single	551 (93.39%)	476 (97.94%)
civil.status	married	33 (5.59%)	9 (1.85%)
civil.status	divorced	5 (0.85%)	1 (0.21%)
civil.status	widower	1 (0.17%)	NA
paternity	no	524 (88.81%)	475 (97.74%)
paternity	yes	66 (11.19%)	11 (2.26%)
minors	no	525 (88.98%)	476 (97.94%)
minors	yes	65 (11.02%)	10 (2.06%)
infants	no	537 (91.02%)	477 (98.15%)
infants	yes	52 (8.81%)	8 (1.65%)
infants	NA	1 (0.17%)	1 (0.21%)
care.older.adults	no	553 (93.73%)	426 (87.65%)
care.older.adults	yes	37 (6.27%)	60 (12.35%)
main.caretaker	no	538 (91.19%)	476 (97.94%)
main.caretaker	yes	52 (8.81%)	10 (2.06%)
mh.diagnosis	no	445 (75.42%)	346 (71.19%)
mh.diagnosis	yes	145 (24.58%)	140 (28.81%)
mh.medication	NA	445 (75.42%)	346 (71.19%)
mh.medication	no	113 (19.15%)	111 (22.84%)
mh.medication	yes	32 (5.42%)	29 (5.97%)
chronic.disease	no	566 (95.93%)	445 (91.56%)

variable	value	nonstudents	students
chronic.disease	yes	24 (4.07%)	41 (8.44%)

Here are the odds ratios. I haven't found a clean way to do this so have used "echo=TRUE" to show what is what.

```
tmpGetOR <- function(x, y){
  epitools::oddsratio.midp(x, y, conf.level = .95)$measure %>%
    as_tibble(rownames = "value") %>%
    filter(row_number() != 1) %>%
    mutate(across(-value, ~ round(.x, 2)),
           CI = str_c("[",
                      lower,
                      ", ",
                      upper,
                      "]"),
           OR = str_c(estimate,
                      " ",
                      CI)) %>%
    select(value, OR) -> tmp
  tmp
}
tmpGetOR(tibBaseline$gender, tibBaseline$group)
```

```
## # A tibble: 2 x 2
##   value OR
##   <chr> <chr>
## 1 male  0.66 [0.51, 0.84]
## 2 other 0.37 [0.01, 3.22]
```

```
tmpGetOR(tibBaseline$living, tibBaseline$group)
```

```
## # A tibble: 5 x 2
##   value        OR
##   <chr>        <chr>
## 1 friends      1.16 [0.15, 5.44]
## 2 living alone 1.59 [0.66, 3.81]
## 3 other        5.74 [2.03, 17]
## 4 parents      4.15 [2.35, 7.84]
## 5 relatives    3.57 [1.67, 7.94]
```

```
tmpGetOR(tibBaseline$housing, tibBaseline$group)
```

```
## # A tibble: 3 x 2
##   value    OR
##   <chr>    <chr>
## 1 other    3.3 [1.01, 12.06]
## 2 own home 1.8 [1.1, 3]
## 3 renting  1.02 [0.59, 1.78]
```

```r
# tmpGetOR(tibBaseline$civil.status, tibBaseline$group)
 # Error in uniroot(function(or) { :
 #  f() values at end points not of opposite sign
tibBaseline %>%
  select(civil.status, group) %>%
  # tabyl(civil.status, group) #%>%
  mutate(civil.status = recode(civil.status,
                               "married" = "married",
                               "single" = "single",
                               "divorced" = "single",
                               "widower" = "single")) -> tmpTib

tmpTib %>%
  tabyl(civil.status, group) %>%
  adorn_percentages(denominator = "col") %>%
  adorn_pct_formatting(digits = 1) %>%
  adorn_ns()
```

```
##  civil.status nonstudents    students
##       married  5.6%  (33)  1.9%   (9)
##        single 94.4% (557) 98.1% (477)
```

```r
table(tmpTib$civil.status, tmpTib$group)
```

```
##
##            nonstudents students
##   married           33        9
##   single           557      477
```

```r
tmpGetOR(tmpTib$civil.status, tmpTib$group)
```

```
## # A tibble: 1 x 2
##   value  OR
##   <chr>  <chr>
## 1 single 3.1 [1.52, 6.99]
```

```r
tmpGetOR(tibBaseline$paternity, tibBaseline$group)
```

```
## # A tibble: 1 x 2
##   value OR
##   <chr> <chr>
## 1 yes   0.19 [0.09, 0.34]
```

```r
tmpGetOR(tibBaseline$minors, tibBaseline$group)
```

```
## # A tibble: 1 x 2
##   value OR
##   <chr> <chr>
## 1 yes   0.17 [0.08, 0.32]
```

```
tmpGetOR(tibBaseline$infants, tibBaseline$group)
```

```
## # A tibble: 1 x 2
##   value OR
##   <chr> <chr>
## 1 yes   0.18 [0.08, 0.36]
```

```
tmpGetOR(tibBaseline$care.older.adults, tibBaseline$group)
```

```
## # A tibble: 1 x 2
##   value OR
##   <chr> <chr>
## 1 yes   2.1 [1.37, 3.25]
```

```
tmpGetOR(tibBaseline$main.caretaker, tibBaseline$group)
```

```
## # A tibble: 1 x 2
##   value OR
##   <chr> <chr>
## 1 yes   0.22 [0.1, 0.42]
```

```
tmpGetOR(tibBaseline$mh.diagnosis, tibBaseline$group)
```

```
## # A tibble: 1 x 2
##   value OR
##   <chr> <chr>
## 1 yes   1.24 [0.95, 1.63]
```

```
tmpGetOR(tibBaseline$mh.medication, tibBaseline$group)
```

```
## # A tibble: 1 x 2
##   value OR
##   <chr> <chr>
## 1 yes   0.92 [0.52, 1.63]
```

```
tmpGetOR(tibBaseline$chronic.disease, tibBaseline$group)
```

```
## # A tibble: 1 x 2
##   value OR
##   <chr> <chr>
## 1 yes   2.17 [1.3, 3.69]
```

```
# tibBaseline %>%
#   select(group, vecDemogVars) %>%
#   pivot_longer(cols = -group, names_to = "variable", values_to = "value") %>%
#   ### turn variable into ordered factor to be able to sort sensibly later
#   mutate(variable = ordered(variable,
#                             levels = vecDemogVars,
```

```
#                               labels = vecDemogVars)) %>%
#   filter(variable == "gender") %>%
#   split(.$variable) %>%
#   map(~ table(.$value, .$group)) %>%
#   map(~ epitools::oddsratio.midp(.data))
```

And now the full chi squared values (though we don't use these in Table 1 and rightly not in my view, reviewers/APA may say different so we have got the necessary values here if they do!) Quitting from lines 1169-1172 (Baseline_code_20211222.Rmd) Error: Can't subset columns that don't exist. x Column `demogVar` doesn't exist. Backtrace: x 1. +-rmarkdown::render(…) 2. | -knitr::knit(knit_input, knit_output, envir = envir, quiet = quiet) 3. | -knitr:::process_file(text, output) 4. | +-base::withCallingHandlers(…) 5. | +-knitr:::process_group(group) 6. | -knitr:::process_group.block(group) 7. | -knitr:::call_block(x) 8. | -knitr:::block_exec(params) 9. | -knitr:::eng_r(options) 10. | +-knitr:::in_dir(…) 11. | -knitr:::evaluate(…) 12. | -evaluate::evaluate(…) 13. | -evaluate:::evaluate_call(…) 14. | +-evaluate:::timing_fn(…) 15. | +-base:::handle(…) 16. | +-base::withCallingHandlers(…) 17. | +-base::withVisible(eval(expr, envir, enclos)) 18. | -base::eval(expr, envir, enclos) 19. | -base::eval(expr, envir, enclos) 20. +-tmpTib %>% select(demogVar:p) %>% pander(justify = "lrrrr") 21. +-pander::pander(., justify = "lrrrr") 22. +-dplyr::select(., demogVar:p) 23. -dplyr:::select.data.frame(., demogVar:p) 24. -tidyselect::eval_select(expr(c(…)), .data) 25. -tidyselect:::eval_select_impl(…) 26. +-tidyselect:::with_subscript_errors(…) 27. | +-base::tryCatch(…) 28. | | -base:::tryCatchList(expr, classes, parentenv, handlers) 29. | | -base:::tryCatchOne(expr, names, parentenv, handlers[[1L]]) 30. | | -base:::doTryCatch(return(expr), name, parentenv, handler) 31. | -tidyselect:::instrument_base_errors(expr) 32. | -base::withCallingHandlers(…) 33. -tidyselect:::vars_select_eval(…) 34. -tidyselect:::walk_data_tree(expr, data_mask, context_mask) 35. -tidyselect:::eval_c(expr, data_mask, context_mask) 36. -tidyselect:::reduce_sels(node, data_mask, context_mask, init = init) 37. -tidyselect:::walk_data_tree(new, data_mask, context_mask) 38. -tidyselect:::eval_colon(expr, data_mask, context_mask) 39. -tidyselect:::walk_data_tree(…) 40. -tidyselect:::as_indices_sel_impl(…) 41. -tidyselect:::as_indices_impl(x, vars, strict = strict) 42. -tidyselect:::chr_as_locations(x, vars) 43. -vctrs::vec_as_location(x, n = length(vars), names = vars) 44. -(function () … 45. -vctrs:::stop_subscript_oob(…) 46. -vctrs:::stop_subscript(…) There were 21 warnings (use warnings() to see them)

[3] (Table 2): continuous variables

```
## # A tibble: 8 x 23
##   variable              nMiss obsmean_NS obsmean_S LCLmean_NS LCLmean_S UCLmean_NS UCLmean_S SD_NS  S
##   <chr>                 <int>      <dbl>     <dbl>      <dbl>     <dbl>      <dbl>     <dbl> <dbl> <d
## 1 age                       0     23.9      21.7        23.6      21.5        24.1      21.9    2.79  2
## 2 exercising.week2          0      2.58      2.22        2.43      2.05        2.74      2.40   2.00  1
## 3 financial.dependency2     0      2.03      2.65        1.97      2.60        2.09      2.70   0.749 0
## 4 financial.distress2       0      1.29      1.37        1.22      1.28        1.36      1.45   0.832 0
## 5 hours.social.networks     0      4.73      4.37        4.48      4.14        4.98      4.62   3.18  2
## 6 number.kids               0      0.134     0.0247      0.105     0.0123      0.168     0.0412 0.405 0
## 7 routine2                  0      2.42      2.35        2.33      2.25        2.51      2.44   1.13  1
## 8 sleeping.hours            0      7.15      6.47        7.05      6.35        7.25      6.58   1.22  1
```

```
## # A tibble: 8 x 23
##   variable              nMiss obsmean_NS obsmean_S LCLmean_NS LCLmean_S UCLmean_NS UCLmean_S SD_NS  S
##   <chr>                 <int>      <dbl>     <dbl>      <dbl>     <dbl>      <dbl>     <dbl> <dbl> <d
## 1 routine2                  0      2.42      2.35        2.33      2.25        2.51      2.44   1.13  1
## 2 financial.distress2       0      1.29      1.37        1.22      1.28        1.36      1.45   0.832 0
## 3 hours.social.networks     0      4.73      4.37        4.48      4.14        4.98      4.62   3.18  2
## 4 exercising.week2          0      2.58      2.22        2.43      2.05        2.74      2.40   2.00  1
```

```
## 5 number.kids            0      0.134    0.0247      0.105    0.0123      0.168    0.0412 0.405 0
## 6 sleeping.hours         0      7.15     6.47        7.05     6.35        7.25     6.58   1.22  1
## 7 age                    0     23.9     21.7        23.6     21.5        24.1     21.9    2.79  2
## 8 financial.dependency2  0      2.03     2.65        1.97     2.60        2.09     2.70   0.749 0
```

Table 4: continuous dependent variables

```
## # A tibble: 7 x 22
##   variable     nMiss obsmean_NS obsmean_S LCLmean_NS LCLmean_S UCLmean_NS UCLmean_S   SD_NS    SD_S
##   <chr>        <int>      <dbl>     <dbl>      <dbl>     <dbl>      <dbl>     <dbl>   <dbl>   <dbl>
## 1 COREnr           0      1.16      1.32       1.11      1.26       1.21      1.38  0.644   0.692
## 2 COREriskk        0      0.231     0.240      0.202     0.207      0.262     0.279 0.395   0.399
## 3 COREtotal        0      0.993     1.13       0.947     1.08       1.03      1.19  0.574   0.615
## 4 EQ.index         0      0.954     0.935      0.948     0.928      0.958     0.941 0.0633  0.0761
## 5 EQ.VAS           0     76.1      70.2       74.3      68.1       77.9      72.3  22.3    23.2
## 6 WGO.negative     0      3.81      3.61       3.66      3.44       3.96      3.77  1.76    1.77
## 7 WGO.positive     0      5.83      5.65       5.72      5.55       5.93      5.77  1.27    1.33
```

Reordered.

```
## # A tibble: 7 x 22
##   variable     nMiss obsmean_NS obsmean_S LCLmean_NS LCLmean_S UCLmean_NS UCLmean_S   SD_NS    SD_S
##   <chr>        <int>      <dbl>     <dbl>      <dbl>     <dbl>      <dbl>     <dbl>   <dbl>   <dbl>
## 1 COREriskk        0      0.231     0.240      0.202     0.207      0.262     0.279 0.395   0.399
## 2 WGO.negative     0      3.81      3.61       3.66      3.44       3.96      3.77  1.76    1.77
## 3 WGO.positive     0      5.83      5.65       5.72      5.55       5.93      5.77  1.27    1.33
## 4 COREtotal        0      0.993     1.13       0.947     1.08       1.03      1.19  0.574   0.615
## 5 COREnr           0      1.16      1.32       1.11      1.26       1.21      1.38  0.644   0.692
## 6 EQ.VAS           0     76.1      70.2       74.3      68.1       77.9      72.3  22.3    23.2
## 7 EQ.index         0      0.954     0.935      0.948     0.928      0.958     0.941 0.0633  0.0761
```

Associations and interactions of variables

One aim behind the full study was to fill a gap in our knowledge caused because, while there is a considerable body of work on mental health and wellbeing in university students, there is very little in young adults of similar age who are not students. Accordingly, the study has two study groups: students and non-students and one focus is on differences between the groups. However, as this is survey data not a designed, balanced group experiment, group is likely to be associated with other variables. This section of this report looks at:
* associations between group and demographic variables and
* between demographic variables within group then at
* how these associations inevitably confound simple group differences on the dependent variables.

The specific clause in the DAP is this:

> where bivariate associations between tibBaseline variables (including students/non-student group) are statistically significant at p < .01, the joint relationship of both variables and the dependent variables will be reported with the interaction terms as an exploration of these complexities

The key predictor variables are *group* i.e. students *vs.* nonstudents, E *vs.* NE: the crucial group variable, and then the demographic variables that I have in *vecOthPreds*: *gender*, *age*, *city*, *living*,

housing, *civil.status*, *financial.dependency*, *financial.distress*, *paternity*, *number.kids*, *main.caretaker*, *minors*, *infants*, *care.older.adults*, *sleeping.hours*, *routine*, *exercising.week*, *hours.social.networks*, *mh.diagnosis*, *mh.medication* and *chronic.disease*

The dependent variables for the purposes of this exploration are in *vecDeps*: *EQ.VAS*, *EQ.index*, *COREtotal*, *CORErisk*, *COREnr*, *WGO.positive* and *WGO.negative*.

We have data from 1076 participants with the breakdown by group as follows.

group	n
nonstudents	590
students	486

[3] Are there differences between the two groups (E/NE) at p < .01?

This is our starting point. Look at this, then look at associations of predictors within the two groups, then look at relationships between predictors and dependents within groups and finally look at multivariate predictions. Ooh, this is messier than I was really describing!

Dependent variables

CORE scores These are all numeric. Look at differences between groups as all this is about seeing how much the other predictors may be contributing to, or reducing/hiding, any relationships between groups on the dependents. I will start with the CORE variables. Here are simple descriptives by group.

group	variable	n	nNA	propNA	min	max	mean	SD	median
nonstudents	COREnr	590	0	0	0	3.214	1.16	0.64	1.07
nonstudents	CORErisk	590	0	0	0	2.667	0.23	0.39	0
nonstudents	COREtotal	590	0	0	0.02941	2.971	0.99	0.57	0.88
students	COREnr	486	0	0	0	3.679	1.32	0.69	1.29
students	CORErisk	486	0	0	0	2	0.24	0.4	0
students	COREtotal	486	0	0	0	3.265	1.13	0.62	1.06

And here are histograms.

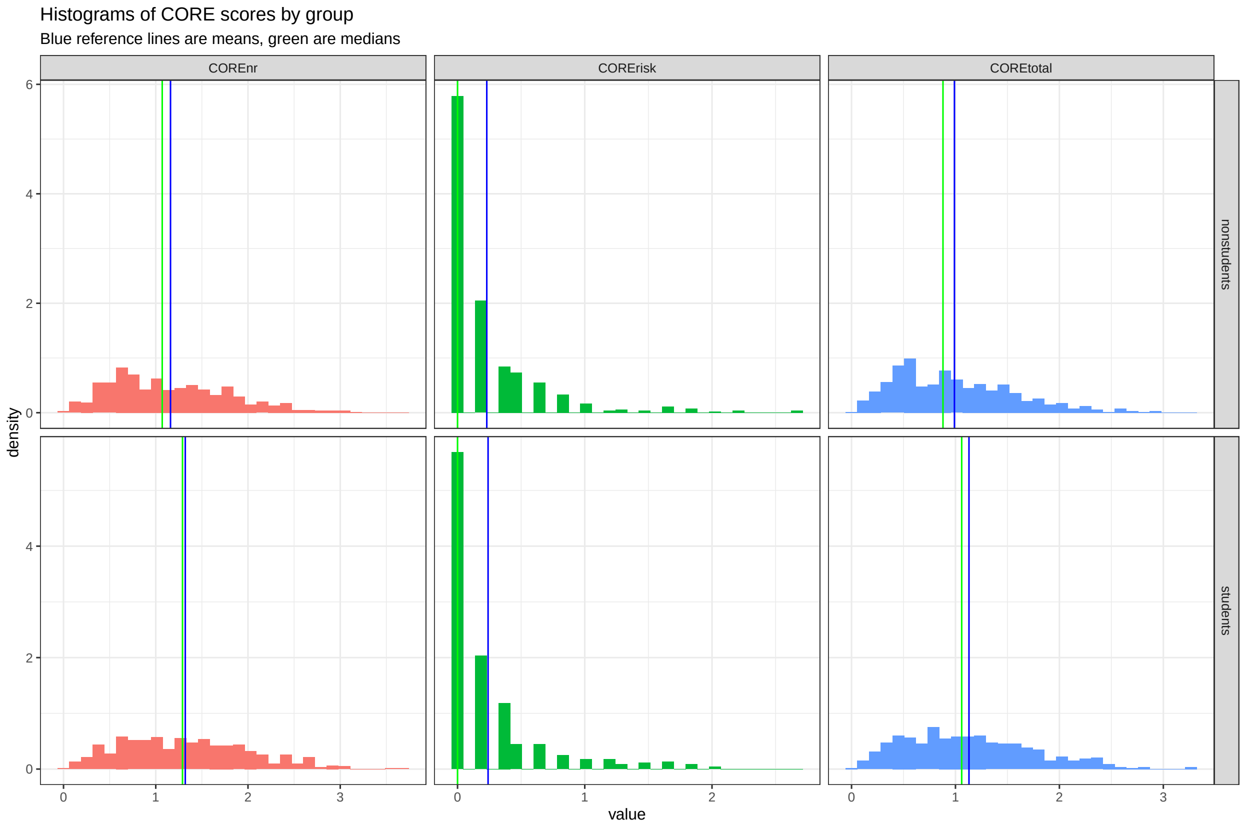

Hm. None of those look truly Gaussian and we are hypothesis testing and we have large numbers so I'm sure we are correct to use Mann-Whitney/Wilcoxon testing. However, when we come to look at interations we will have to go to ANOVA I think.

variable	group1	group2	n1	n2	statistic	p	plt01
COREnr	nonstudents	students	590	486	122922	5.55e-05	TRUE
CORErisk	nonstudents	students	590	486	141916	0.755	FALSE
COREtotal	nonstudents	students	590	486	123952	0.000129	TRUE

Fair enough! What about the effect sizes.

variable	mean1	meanNE	meanE	sdNE	sdE	Parameter	Eta2	CI	CI_low	CI_high	Cohen1992
COREnr	1.232	1.156	1.324	0.6442	0.6919	group	0.01552	0.95	0.004272	0.0333	very small
CORErisk	0.2351	0.2308	0.2404	0.395	0.399	group	0.0001454	0.95	0	0.004892	very small
COREtotal	1.056	0.993	1.133	0.5743	0.6155	group	0.01363	0.95	0.003296	0.03055	very small

So although group membership is statistically significantly related to CORE.nr and CORE.total scores at p < .01, the effect sizes are very small.

EQ scores

group	variable	n	nNA	propNA	min	max	mean	SD	median
nonstudents	EQ.index	590	0	0	0.632	1	0.95	0.06	0.96
nonstudents	EQ.VAS	590	0	0	1	100	76.1	22.33	82
students	EQ.index	486	0	0	0.422	1	0.93	0.08	0.96
students	EQ.VAS	486	0	0	1	100	70.22	23.16	74.5

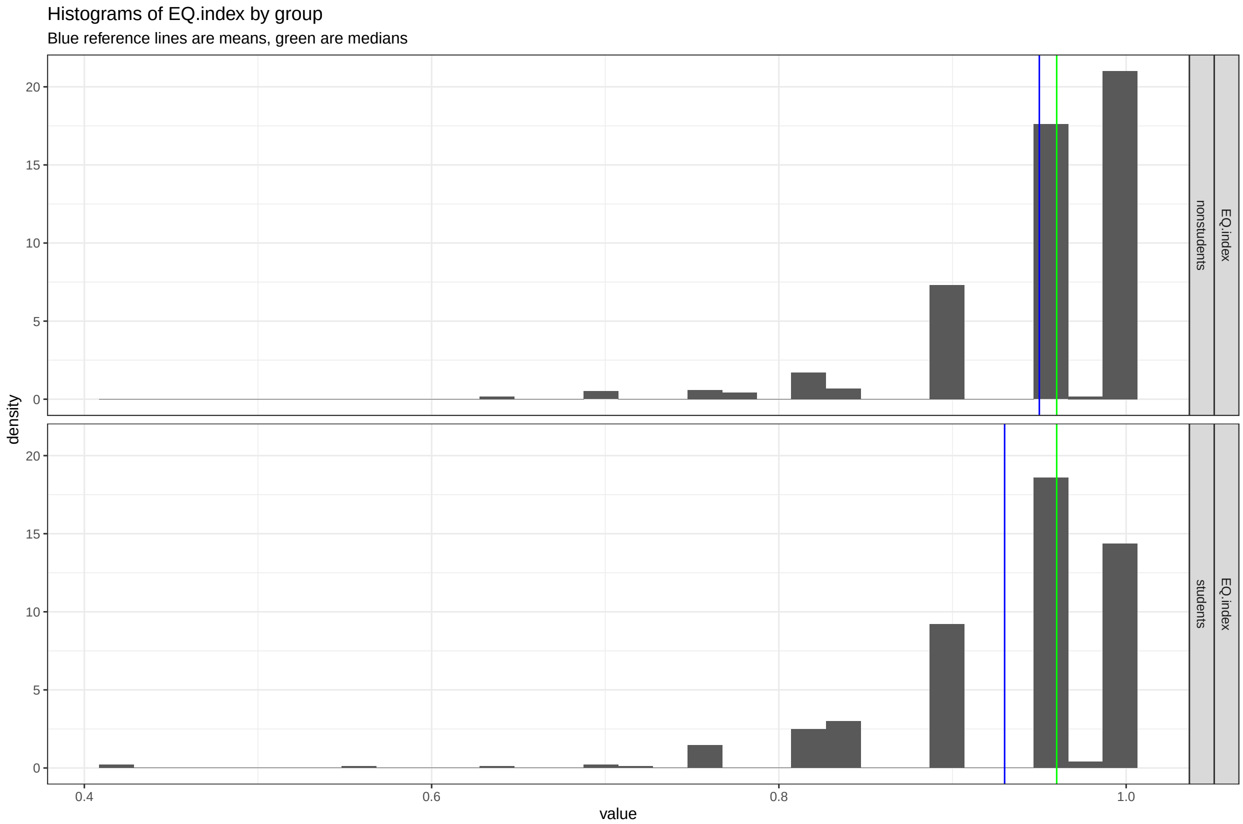

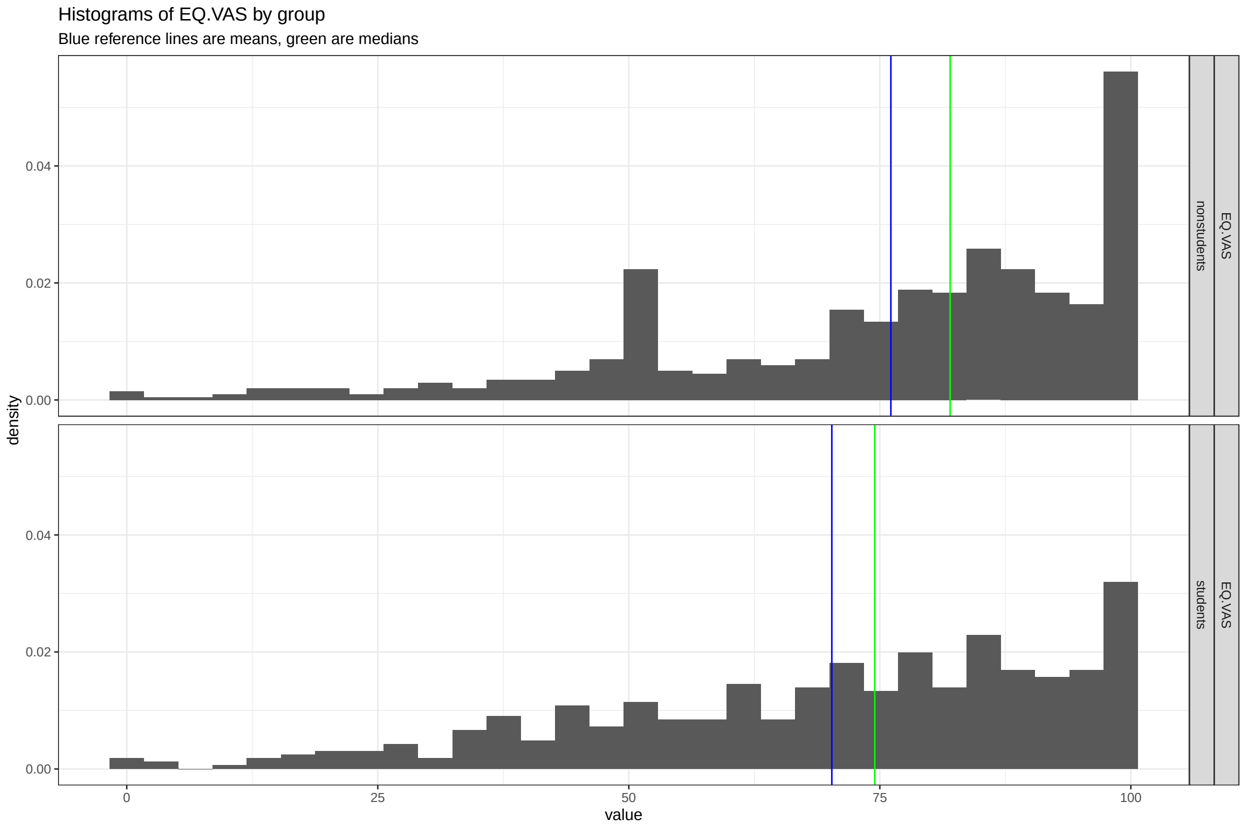

Again, nowhere near Gaussian.

variable	group1	group2	n1	n2	statistic	p	plt01
EQ.index	nonstudents	students	590	486	168179	3.17e-07	TRUE
EQ.VAS	nonstudents	students	590	486	167036	2.98e-06	TRUE

Clear!

variable	mean1	meanNE	meanE	sdNE	sdE	Parameter	Eta2	CI	CI_low	CI_high	Cohen1992
EQ.index	0.945	0.9535	0.9346	0.06326	0.0761	group	0.01804	0.95	0.005657	0.03684	very small
EQ.VAS	73.45	76.1	70.22	22.33	23.16	group	0.01637	0.95	0.004726	0.03449	very small

Both statistically significant at $p < .01$ but again, effect sizes very small.

Finally, the WGO.

group	variable	n	nNA	propNA	min	max	mean	SD	median
nonstudents	WGO.negative	590	0	0	1	7	3.81	1.76	3.5
nonstudents	WGO.positive	590	0	0	1	7	5.83	1.27	6
students	WGO.negative	486	0	0	1	7	3.61	1.77	3
students	WGO.positive	486	0	0	1	7	5.65	1.33	6

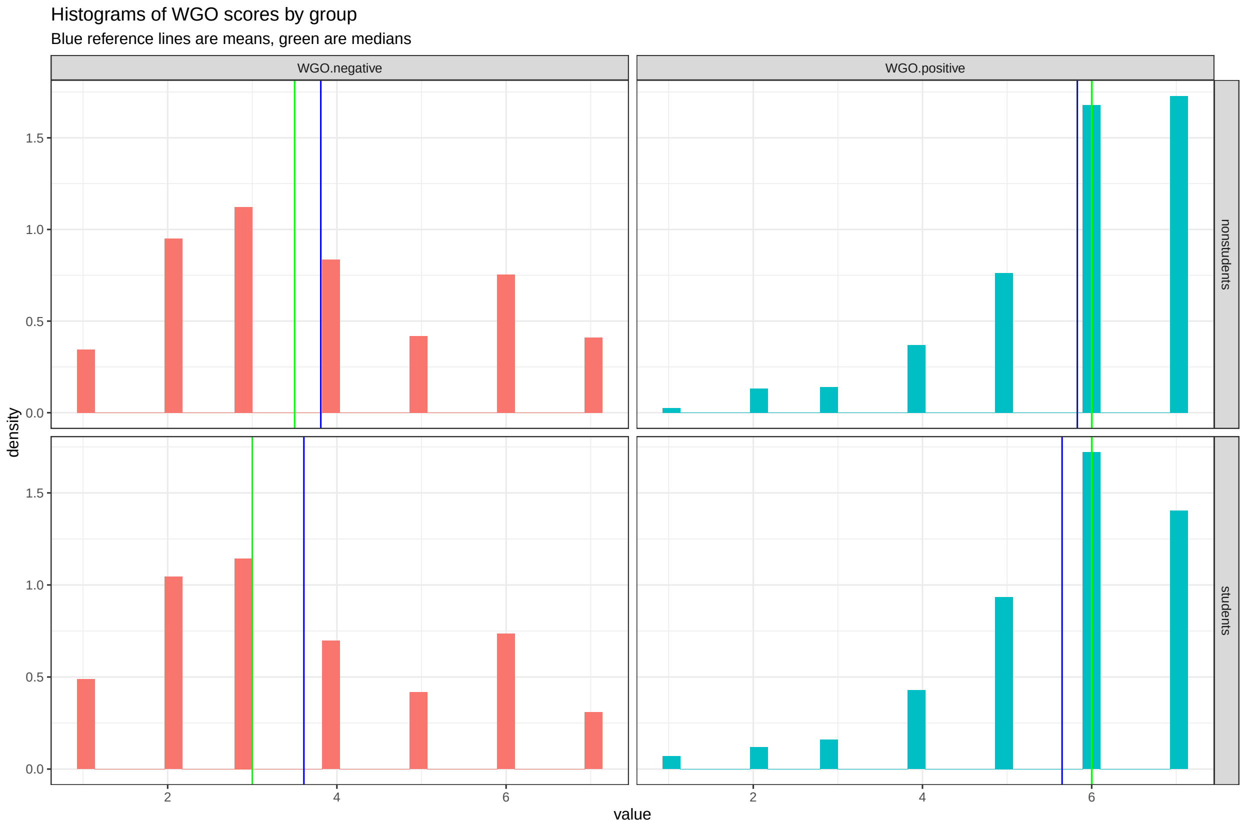

Again, nowhere near Gaussian.

variable	group1	group2	n1	n2	statistic	p	plt01
WGO.negative	nonstudents	students	590	486	153128	0.0507	FALSE
WGO.positive	nonstudents	students	590	486	155508	0.0124	FALSE

Hm, WGO.positive is only missing by a whisker!

variable	mean1	meanNE	meanE	sdNE	sdE	Parameter	Eta2	CI	CI_low	CI_high	Cohen1992
WGO.negative	3.722	3.814	3.611	1.761	1.77	group	0.003253	0.95	0	0.0135	very small
WGO.positive	5.747	5.827	5.65	1.27	1.33	group	0.00459	0.95	0	0.01606	very small

[4] Correlations between dependent variables

EQ-Index and CORE

group	scale	obsCorr	LCLCorr	UCLCorr
nonstudents	COREtotal	-0.5847	-0.6474	-0.5186
nonstudents	COREnr	-0.573	-0.6318	-0.5071
nonstudents	COREtisk	-0.4568	-0.5543	-0.3444

group	scale	obsCorr	LCLCorr	UCLCorr
students	COREtotal	-0.5598	-0.6263	-0.4844
students	COREnr	-0.5567	-0.6219	-0.4837
students	CORErisk	-0.3881	-0.4838	-0.2884

EQ-VAS and CORE

group	scale	obsCorr	LCLCorr	UCLCorr
nonstudents	COREtotal	-0.5303	-0.601	-0.4535
nonstudents	COREnr	-0.5292	-0.6018	-0.4498
nonstudents	CORErisk	-0.3421	-0.4325	-0.2406
students	COREtotal	-0.5539	-0.6288	-0.4697
students	COREnr	-0.5541	-0.6246	-0.4764
students	CORErisk	-0.3574	-0.4483	-0.2617

Correlations with the WGO

WGO positive

group	scale	obsCorr	LCLCorr	UCLCorr
nonstudents	COREtotal	-0.4985	-0.5635	-0.4271
nonstudents	COREnr	-0.4949	-0.5605	-0.4268
nonstudents	CORErisk	-0.3411	-0.4247	-0.2534
nonstudents	EQ.index	0.2495	0.1522	0.3407
nonstudents	EQ.VAS	0.2657	0.1773	0.3495
students	COREtotal	-0.4819	-0.5579	-0.4
students	COREnr	-0.4892	-0.5645	-0.4043
students	CORErisk	-0.253	-0.3465	-0.1525
students	EQ.index	0.2712	0.1752	0.3605
students	EQ.VAS	0.2477	0.1444	0.3492

WGO positive and CORE total

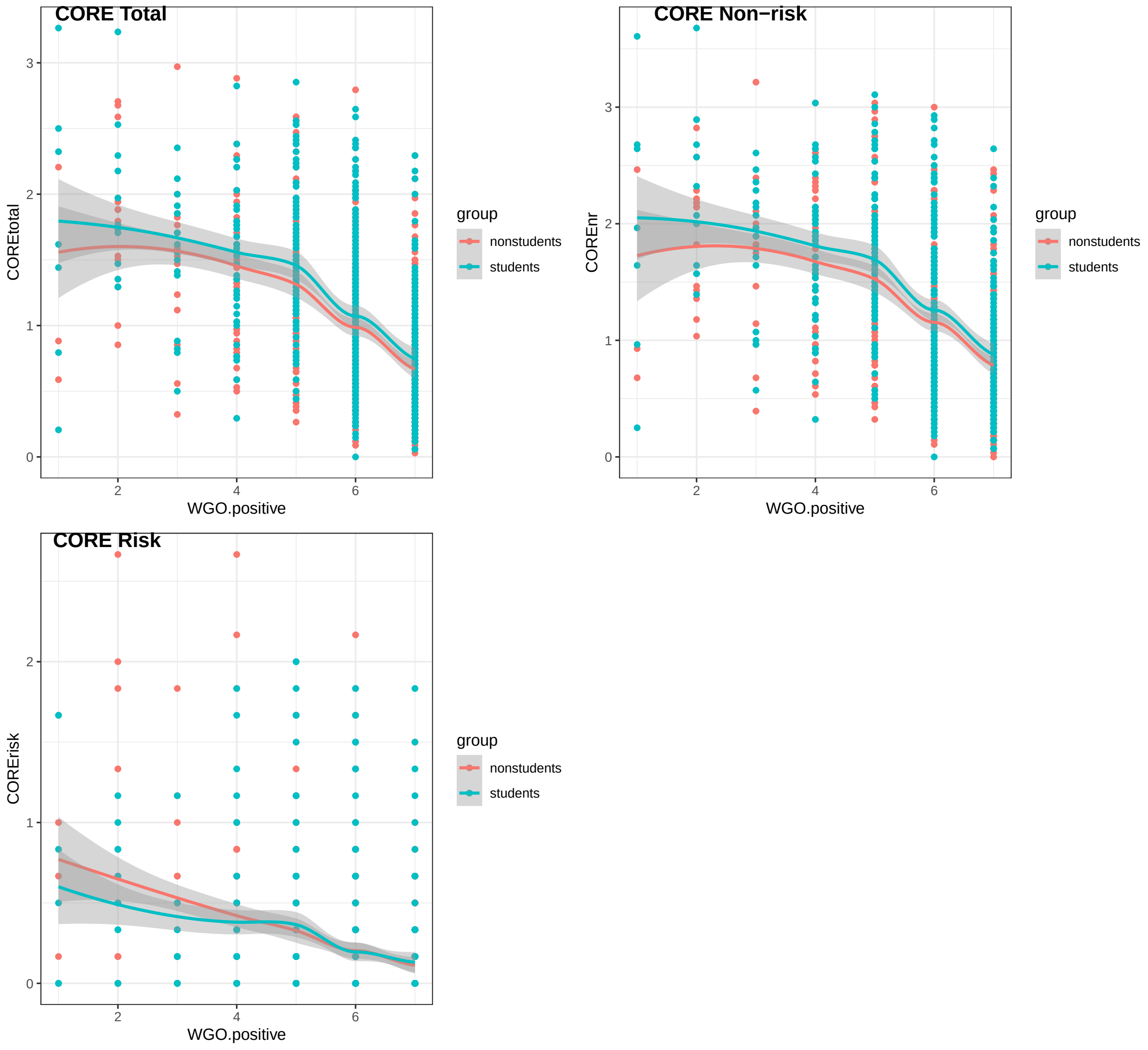

WGO positive and EQ

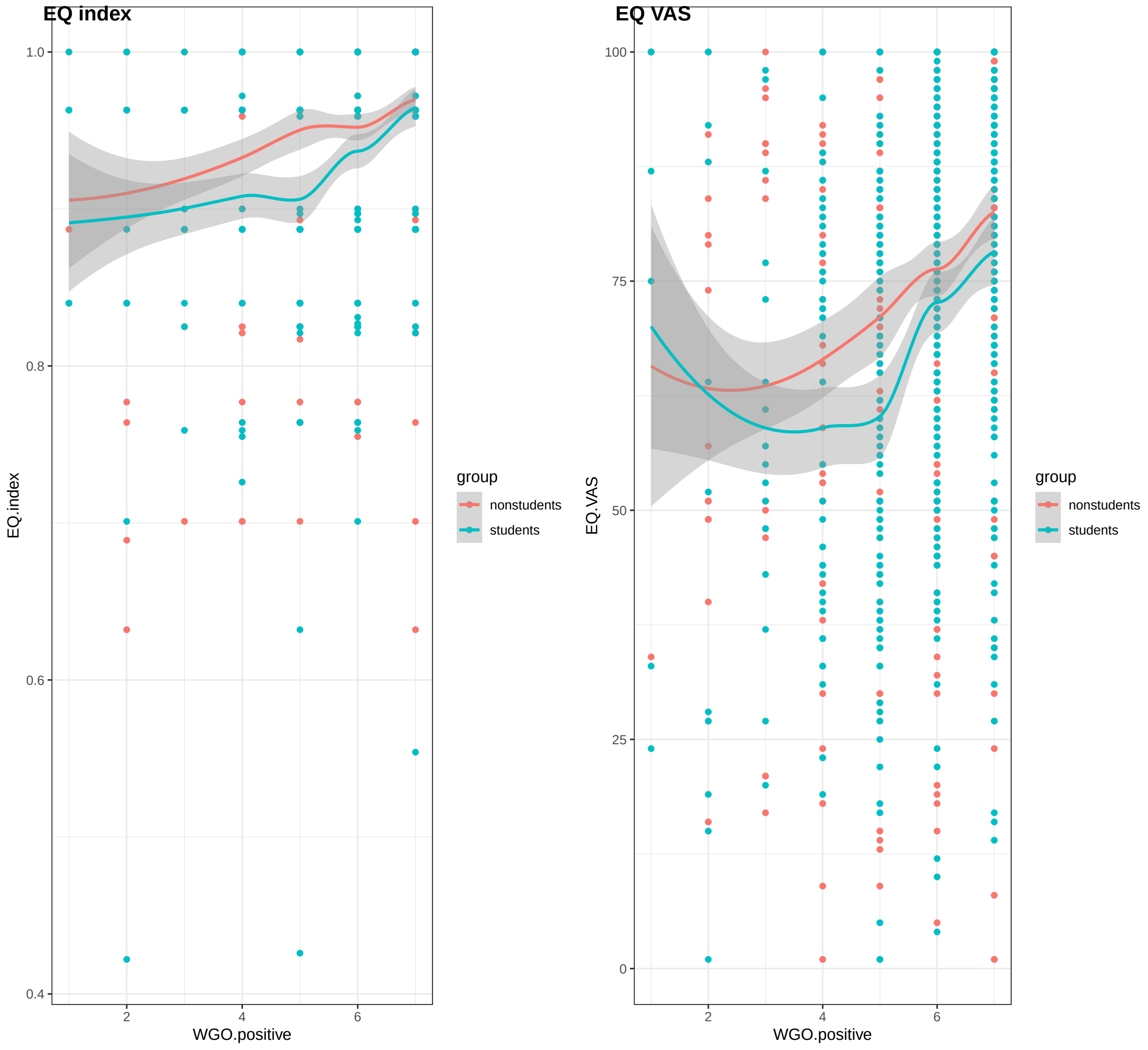

WGO negative

group	scale	obsCorr	LCLCorr	UCLCorr
nonstudents	COREtotal	-0.4841	-0.5372	-0.4271
nonstudents	COREnr	-0.4961	-0.5515	-0.4401
nonstudents	CORErisk	-0.2134	-0.275	-0.1484
nonstudents	EQ.index	0.3321	0.2653	0.3997
nonstudents	EQ.VAS	0.2896	0.2138	0.3668
students	COREtotal	-0.4392	-0.5084	-0.3595
students	COREnr	-0.4375	-0.5099	-0.3619
students	CORErisk	-0.298	-0.3685	-0.2184
students	EQ.index	0.265	0.1868	0.3467
students	EQ.VAS	0.2474	0.1607	0.3316

WGO negative and CORE total

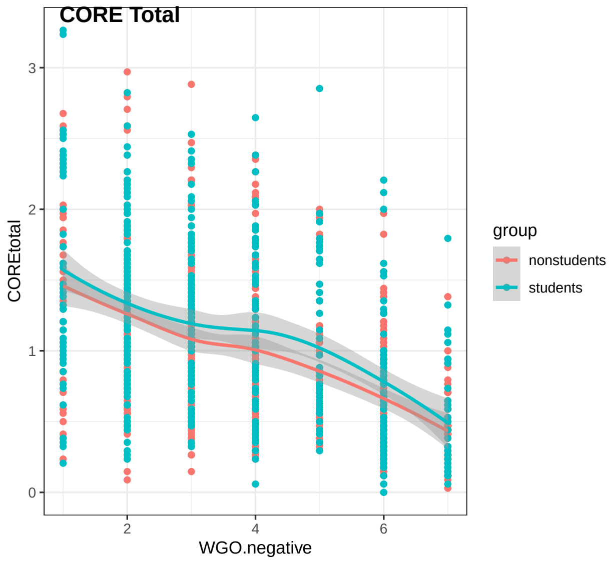

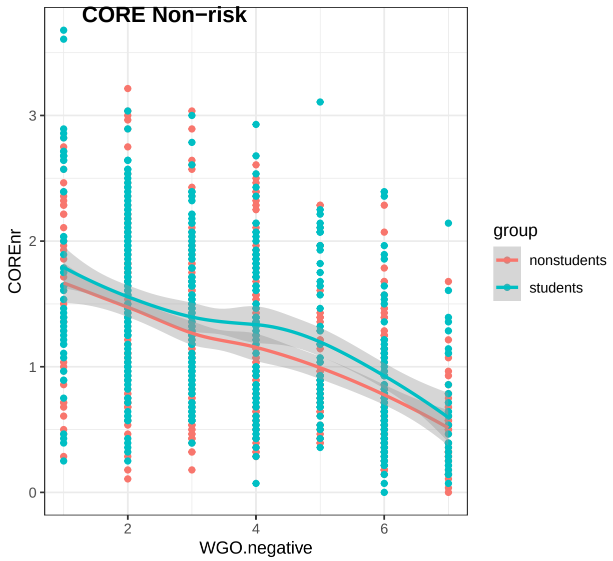

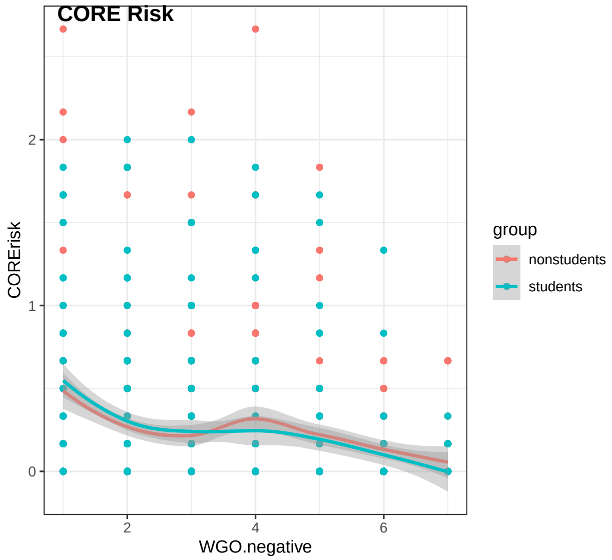

WGO negative and EQ

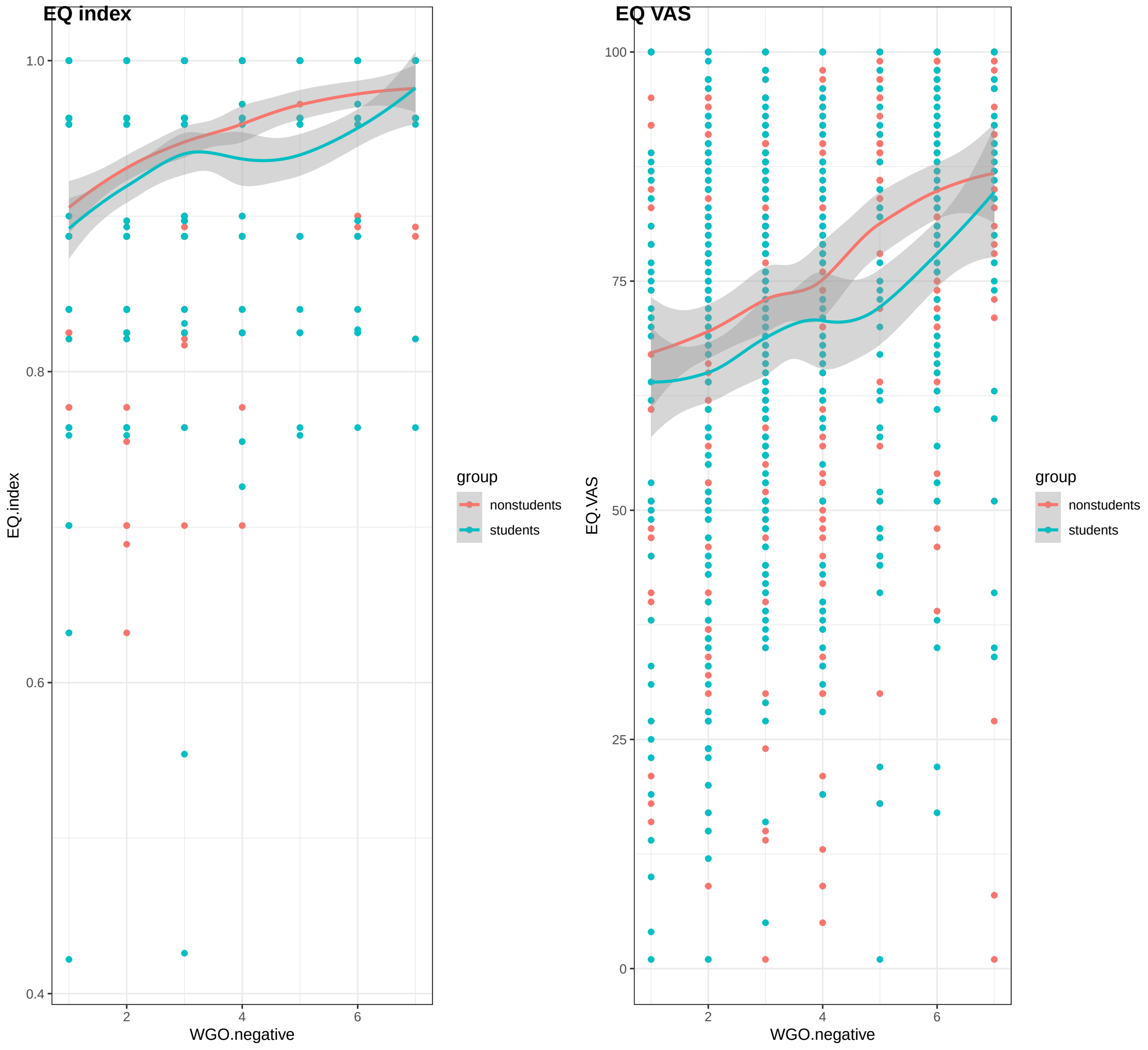

[9] Distributions

group	scale	n	min	pct95th	95CILCL	95CIUCL	max
nonstudents	COREtotal	590	0.02941	2.029	1.949	2.206	2.971
nonstudents	COREnr	590	0	2.357	2.259	2.464	3.214
nonstudents	COORErisk	590	0	1	0.8333	1.317	2.667
nonstudents	EQ.index	590	0.632	1	1	1	1
nonstudents	EQ.VAS	590	1	100	100	100	100
students	COREtotal	486	0	2.287	2.176	2.382	3.265
students	COREnr	486	0	2.571	2.408	2.679	3.679
students	COORErisk	486	0	1.167	1	1.5	2
students	EQ.index	486	0.422	1	1	1	1

group	scale	n	min	pct95th	95CILCL	95CIUCL	max
students	EQ.VAS	486	1	100	100	100	100

Summarising relationships between dependents and group

- CORE total and NR scores have significant relationships with group at p < .01 but not the risk score
- EQ.VAS and EQ.index both have significant relationshipp with group at p < .01
- neither WGO score is significantly related to group (though WGO.positive nearly is!)
- all effect sizes are very small. I've plotted the η^2 with the 95% confidence intervals below.

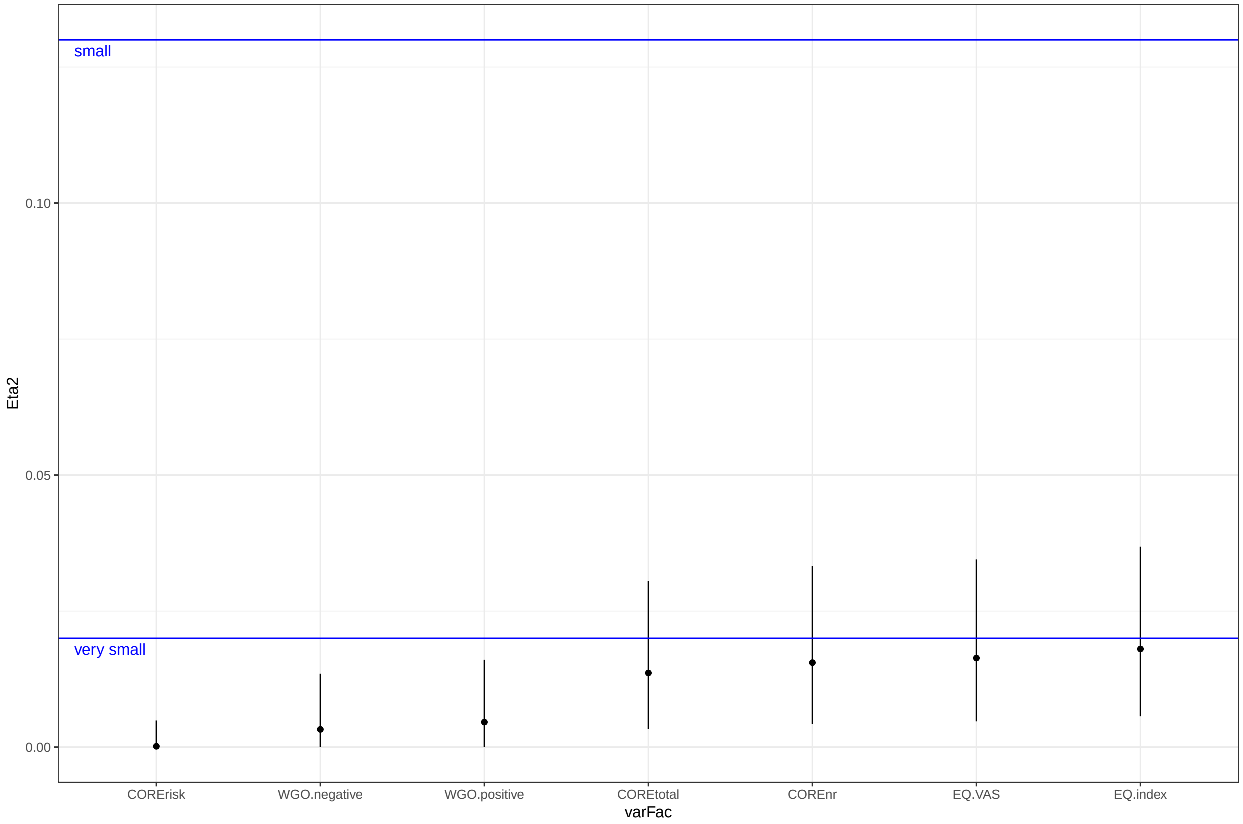

Again that confirms that all the η^2 values are in Cohen's "very small" range though some upper CLs do stretch into the "small" range. I'm not a great fan of those ranges and what will matter is how they compare with the effect sizes for other predictors.

Relationships between categorical predictors and group

Let's look at associations between categories without splitting by group. Simple descriptives first.

variable	n	nNA	propNA	nLevels	level1	level2	level3	level4	level5	level6
care.older.adults	1076	0	0	2	no	yes	NA	NA	NA	NA
chronic.disease	1076	0	0	2	no	yes	NA	NA	NA	NA
city	1076	0	0	1	Quito	NA	NA	NA	NA	NA
civil.status	1076	0	0	4	single	married	divorced	widower	NA	NA

variable	n	nNA	propNA	nLevels	level1	level2	level3	level4	level5	level6
exercising.week	1076	0	0	6	2 or 3	1	never	5	4	more than 5
financial.dependency	1076	0	0	3	totally dependent	totally independent	middle dependency	NA	NA	NA
financial.distress	1076	0	0	4	no	occassionally	frequently	constant	NA	NA
gender	1076	0	0	3	female	male	other	NA	NA	NA
housing	1076	0	0	4	own home	renting	borrowed	other	NA	NA
infants	1076	2	0	2	no	yes	NA	NA	NA	NA
living	1076	0	0	6	parents	couple or kids	living alone	relatives	other	friends
main.caretaker	1076	0	0	2	no	yes	NA	NA	NA	NA
mh.diagnosis	1076	0	0	2	no	yes	NA	NA	NA	NA
mh.medication	1076	791	0.74	2	yes	no	NA	NA	NA	NA
minors	1076	0	0	2	no	yes	NA	NA	NA	NA
paternity	1076	0	0	2	no	yes	NA	NA	NA	NA
routine	1076	0	0	5	many times	sometimes	seldom	always	never	NA

OK.

- Everyone has *city* == "Quito" so that can go.
- I have recoded *civil.status* to "married"/"single".
- I have recoded *exercising.week* to 1 to 6 and numeric as it's a clear ordered quantitative variable.
- I have recoded *financial.dependency* and *financial.distress* and *routine* to numeric too.

So now we have these categorical variables.

variable	n	nNA	propNA	nLevels	level1	level2	level3	level4	level5	level6
care.older.adults	1076	0	0	2	no	yes	NA	NA	NA	NA
chronic.disease	1076	0	0	2	no	yes	NA	NA	NA	NA
civil.status	1076	0	0	2	single	married	NA	NA	NA	NA
gender	1076	0	0	3	female	male	other	NA	NA	NA
housing	1076	0	0	4	own home	renting	borrowed	other	NA	NA
infants	1076	2	0	2	no	yes	NA	NA	NA	NA
living	1076	0	0	6	parents	couple or kids	living alone	relatives	other	friends
main.caretaker	1076	0	0	2	no	yes	NA	NA	NA	NA
mh.diagnosis	1076	0	0	2	no	yes	NA	NA	NA	NA
mh.medication	1076	791	0.74	2	yes	no	NA	NA	NA	NA
minors	1076	0	0	2	no	yes	NA	NA	NA	NA
paternity	1076	0	0	2	no	yes	NA	NA	NA	NA

And applying the chi-squared test to the association of each of those with group membership gets the following.

variable	chisq	parameter.df	p.value	plt01
minors	31.62	1	1.874e-08	TRUE
paternity	30.61	1	3.16e-08	TRUE
living	36.7	5	6.876e-07	TRUE
infants	24.65	1	6.886e-07	TRUE
main.caretaker	21.17	1	4.197e-06	TRUE
housing	19.27	3	0.00024	TRUE
care.older.adults	11.26	1	0.0007922	TRUE
civil.status	8.972	1	0.002741	TRUE
gender	11.79	2	0.002757	TRUE
chronic.disease	8.206	1	0.004174	TRUE
mh.diagnosis	2.237	1	0.1348	FALSE
mh.medication	0.01804	1	0.8932	FALSE

So we have ten categorical variables associated with group with p < .01. They are: *minors*, *paternity*, *living*, *infants*, *main.caretaker*, *housing*, *care.older.adults*, *civil.status*, *gender* and *chronic.disease*. Only the two mental health questions are *not* associated with group at p < .01.

Relationships between numeric predictors and group

OK, descriptives and then very cramped histograms.

group	variable	n	nNA	propNA	min	max	mean	SD	median
nonstudents	age	590	0	0	18	29	23.86	2.79	24
nonstudents	exercising.week	590	0	0	1	6	3.08	1.51	3
nonstudents	financial.dependency	590	0	0	1	3	2.03	0.75	2
nonstudents	financial.distress	590	0	0	1	4	2.29	0.83	2
nonstudents	hours.social.networks	590	0	0	0	24	4.73	3.18	4
nonstudents	number.kids	590	0	0	0	3	0.13	0.4	0
nonstudents	routine	590	0	0	1	5	3.42	1.13	3
nonstudents	sleeping.hours	590	0	0	4	15	7.15	1.22	7
students	age	486	0	0	18	29	21.69	2.15	21
students	exercising.week	486	0	0	1	6	2.79	1.48	3
students	financial.dependency	486	0	0	1	3	2.65	0.59	3
students	financial.distress	486	0	0	1	4	2.37	0.94	2
students	hours.social.networks	486	0	0	0	20	4.37	2.72	4
students	number.kids	486	0	0	0	2	0.02	0.17	0
students	routine	486	0	0	1	5	3.35	1.04	3
students	sleeping.hours	486	0	0	1	12	6.47	1.33	6

Looks as if there is a difference in the proportions who have children between the two groups but no point in analysing *number.kids* given the cell sizes.

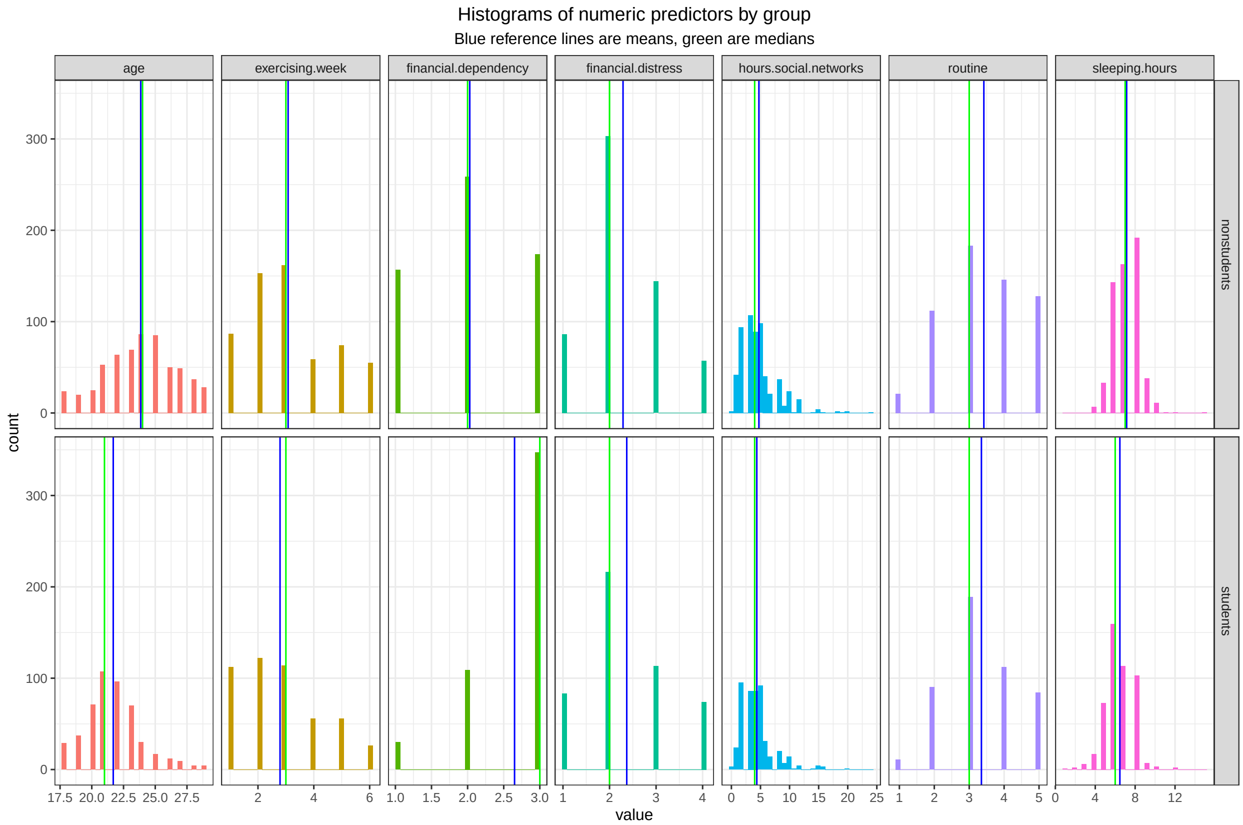

Again, none of those are really Gaussian so go non-parametric.

variable	group1	group2	n1	n2	statistic	p	plt01
financial.dependency	nonstudents	students	590	486	78616	4.99e-44	TRUE
age	nonstudents	students	590	486	210831	7.89e-41	TRUE
sleeping.hours	nonstudents	students	590	486	185002	2.51e-17	TRUE
exercising.week	nonstudents	students	590	486	158650	0.00207	TRUE
hours.social.networks	nonstudents	students	590	486	150418	0.16	FALSE
routine	nonstudents	students	590	486	149512	0.209	FALSE
financial.distress	nonstudents	students	590	486	138227	0.277	FALSE

Clear enough! We have four of these numeric variables associated with group at $p < .01$.

Within group relationships betweeen numeric predictor variables and dependent variables

Start with the nonstudents, showing just correlations significant at $p < .01$.

var1	var2	cor	statistic	p	conf.low	conf.high	plt01
age	COREtotal	-0.2	-4.958	9.34e-07	-0.2766	-0.1216	TRUE
age	COREnr	-0.2	-4.949	9.75e-07	-0.2762	-0.1212	TRUE
age	WGO.positive	0.13	3.229	0.00131	0.05182	0.2105	TRUE
age	CORErisk	-0.13	-3.145	0.00175	-0.2072	-0.0484	TRUE

var1	var2	cor	statistic	p	conf.low	conf.high	plt01
exercising.week	COREnr	-0.23	-5.626	2.85e-08	-0.3012	-0.148	TRUE
exercising.week	COREtotal	-0.22	-5.514	5.27e-08	-0.2971	-0.1436	TRUE
exercising.week	EQ.VAS	0.17	4.215	2.89e-05	0.0918	0.2485	TRUE
exercising.week	EQ.index	0.15	3.745	0.000198	0.07281	0.2305	TRUE
exercising.week	WGO.negative	0.15	3.615	0.000326	0.06753	0.2255	TRUE
exercising.week	WGO.positive	0.14	3.414	0.000684	0.05937	0.2177	TRUE
exercising.week	COREirisk	-0.11	-2.603	0.00948	-0.1858	-0.02623	TRUE
financial.dependency	COREnr	0.22	5.383	1.06e-07	0.1384	0.2923	TRUE
financial.dependency	COREtotal	0.21	5.253	2.1e-07	0.1333	0.2875	TRUE
financial.dependency	WGO.positive	-0.18	-4.355	1.57e-05	-0.2539	-0.09742	TRUE
financial.dependency	EQ.index	-0.11	-2.782	0.00557	-0.1929	-0.03358	TRUE
financial.distress	COREnr	0.35	9.15	9.3e-19	0.2803	0.4217	TRUE
financial.distress	COREtotal	0.35	9.106	1.32e-18	0.2787	0.4203	TRUE
financial.distress	EQ.index	-0.27	-6.839	2e-11	-0.3446	-0.195	TRUE
financial.distress	EQ.VAS	-0.24	-5.893	6.4e-09	-0.3109	-0.1584	TRUE
financial.distress	COREirisk	0.21	5.206	2.67e-07	0.1314	0.2858	TRUE
financial.distress	WGO.negative	-0.21	-5.107	4.42e-07	-0.2821	-0.1275	TRUE
financial.distress	WGO.positive	-0.19	-4.568	5.99e-06	-0.2619	-0.106	TRUE
hours.social.networks	COREtotal	0.22	5.351	1.26e-07	0.1371	0.2911	TRUE
hours.social.networks	COREnr	0.21	5.337	1.35e-07	0.1366	0.2906	TRUE
hours.social.networks	EQ.VAS	-0.15	-3.592	0.000355	-0.2246	-0.06662	TRUE
hours.social.networks	COREirisk	0.14	3.417	0.000677	0.05948	0.2178	TRUE
hours.social.networks	EQ.index	-0.14	-3.32	0.000957	-0.214	-0.05553	TRUE
routine	COREnr	-0.25	-6.186	1.16e-09	-0.3215	-0.1698	TRUE
routine	COREtotal	-0.24	-6.035	2.81e-09	-0.3161	-0.164	TRUE
routine	WGO.negative	0.16	3.886	0.000113	0.07853	0.236	TRUE
routine	EQ.index	0.15	3.778	0.000174	0.07414	0.2318	TRUE
routine	WGO.positive	0.15	3.657	0.000278	0.06923	0.2271	TRUE
routine	EQ.VAS	0.13	3.134	0.00181	0.04796	0.2068	TRUE
routine	COREirisk	-0.11	-2.655	0.00814	-0.1879	-0.02838	TRUE
sleeping.hours	WGO.negative	0.12	2.948	0.00333	0.04034	0.1994	TRUE
sleeping.hours	EQ.index	0.12	2.94	0.00341	0.04004	0.1992	TRUE

Now within the students, again, just the correlations significant at p < .01.

var1	var2	cor	statistic	p	conf.low	conf.high	plt01
exercising.week	COREtotal	-0.25	-5.727	1.8e-08	-0.3334	-0.1667	TRUE
exercising.week	COREnr	-0.25	-5.716	1.91e-08	-0.333	-0.1662	TRUE
exercising.week	EQ.index	0.22	4.995	8.25e-07	0.1351	0.3043	TRUE
exercising.week	EQ.VAS	0.18	3.951	8.95e-05	0.08921	0.2616	TRUE
exercising.week	COREirisk	-0.17	-3.721	0.000221	-0.252	-0.07901	TRUE
exercising.week	WGO.negative	0.13	2.864	0.00436	0.04062	0.2156	TRUE
financial.distress	COREnr	0.31	7.235	1.84e-12	0.2298	0.3905	TRUE
financial.distress	COREtotal	0.31	7.204	2.25e-12	0.2286	0.3894	TRUE
financial.distress	EQ.index	-0.2	-4.57	6.19e-06	-0.2871	-0.1166	TRUE
financial.distress	COREirisk	0.19	4.307	2.01e-05	0.105	0.2763	TRUE
financial.distress	WGO.positive	-0.18	-4.094	4.98e-05	-0.2675	-0.09554	TRUE
financial.distress	EQ.VAS	-0.18	-3.94	9.36e-05	-0.2611	-0.08871	TRUE
financial.distress	WGO.negative	-0.17	-3.877	0.00012	-0.2585	-0.08595	TRUE
routine	COREtotal	-0.31	-7.185	2.56e-12	-0.3887	-0.2278	TRUE
routine	COREnr	-0.31	-7.171	2.81e-12	-0.3881	-0.2272	TRUE

var1	var2	cor	statistic	p	conf.low	conf.high	plt01
routine	WGO.positive	0.21	4.828	1.85e-06	0.1279	0.2976	TRUE
routine	EQ.index	0.21	4.822	1.91e-06	0.1276	0.2974	TRUE
routine	CORErisk	-0.21	-4.626	4.8e-06	-0.2894	-0.119	TRUE
routine	EQ.VAS	0.19	4.354	1.63e-05	0.107	0.2783	TRUE
routine	WGO.negative	0.13	2.994	0.00289	0.04648	0.2212	TRUE
sleeping.hours	COREnr	-0.27	-6.248	9.13e-10	-0.3535	-0.1888	TRUE
sleeping.hours	COREtotal	-0.26	-6.043	3.02e-09	-0.3457	-0.1802	TRUE
sleeping.hours	EQ.index	0.21	4.832	1.81e-06	0.128	0.2978	TRUE
sleeping.hours	EQ.VAS	0.19	4.351	1.65e-05	0.1069	0.2782	TRUE
sleeping.hours	WGO.negative	0.12	2.622	0.00901	0.02972	0.2051	TRUE

Now put those results together so we can see them for each group side by side.

var1	var2	cor_E	stat_E	p_E	conf.low_E	conf.high_E	plt01_E	cor_NE	stat_NE	p_NE	conf.low_NE	conf.high_NE	plt01_NE	plt01
age	COREnr	-0.093	-2.062	0.0398	-0.1808	-0.004399	FALSE	-0.2	-4.949	9.75e-07	-0.2762	-0.1212	TRUE	1
age	WGO.positive	0.092	2.031	0.0428	0.003007	0.1794	FALSE	0.13	3.229	0.00131	0.05182	0.2105	TRUE	1
age	COREtotal	-0.085	-1.873	0.0617	-0.1725	0.004164	FALSE	-0.2	-4.958	9.34e-07	-0.2766	-0.1216	TRUE	1
age	EQ.VAS	0.05	1.107	0.269	-0.03888	0.1386	FALSE	0.096	2.336	0.0198	0.01528	0.1752	FALSE	0
age	COREris	0.014	0.3028	0.762	-0.07527	0.1026	FALSE	-0.13	-3.145	0.00175	-0.2072	-0.0484	TRUE	1
age	WGO.negative	-0.013	-0.2901	0.772	-0.102	0.07585	FALSE	0.089	2.165	0.0308	0.008267	0.1684	FALSE	0
age	EQ.index	-0.011	-0.2515	0.802	-0.1003	0.07759	FALSE	0.083	2.03	0.0428	0.002726	0.1631	FALSE	0
exercising.week	COREtotal	-0.25	-5.727	1.8e-08	-0.3334	-0.1667	TRUE	-0.22	-5.514	5.27e-08	-0.2971	-0.1436	TRUE	2
exercising.week	COREnr	-0.25	-5.716	1.91e-08	-0.333	-0.1662	TRUE	-0.23	-5.626	2.85e-08	-0.3012	-0.148	TRUE	2
exercising.week	EQ.index	0.22	4.995	8.25e-07	0.1351	0.3043	TRUE	0.15	3.745	0.000198	0.07281	0.2305	TRUE	2
exercising.week	EQ.VAS	0.18	3.951	8.95e-05	0.08921	0.2616	TRUE	0.17	4.215	2.89e-05	0.0918	0.2485	TRUE	2
exercising.week	COREcrisk	-0.17	-3.721	0.000221	-0.252	-0.07901	TRUE	-0.11	-2.603	0.00948	-0.1858	-0.02623	TRUE	2
exercising.week	WGO.negative	0.13	2.864	0.00436	0.04062	0.2156	TRUE	0.15	3.615	0.000326	0.06753	0.2255	TRUE	2
exercising.week	WGO.positive	0.11	2.48	0.0135	0.02329	0.199	FALSE	0.14	3.414	0.000684	0.05937	0.2177	TRUE	1
financial.dependency	EQ.VAS	-0.11	-2.406	0.0165	-0.1958	-0.01995	FALSE	-0.1	-2.433	0.0153	-0.1791	-0.01927	FALSE	0
financial.dependency	COREnr	0.07	1.539	0.124	-0.01927	0.1578	FALSE	0.22	5.383	1.06e-07	0.1384	0.2923	TRUE	1
financial.dependency	COREtotal	0.064	1.409	0.159	-0.02517	0.152	FALSE	0.21	5.253	2.1e-07	0.1333	0.2875	TRUE	1
financial.dependency	WGO.negative	-0.062	-1.375	0.17	-0.1505	0.02672	FALSE	-0.067	-1.621	0.106	-0.1466	0.01411	FALSE	0
financial.dependency	EQ.index	0.046	1.012	0.312	-0.04315	0.1344	FALSE	-0.11	-2.782	0.00557	-0.1929	-0.03358	TRUE	1
financial.dependency	WGO.positive	-0.013	-0.2944	0.769	-0.1022	0.07566	FALSE	-0.18	-4.355	1.57e-05	-0.2539	-0.09742	TRUE	1

var1	var2	cor_E	stat_E	p_E	conf.low	conf.high	plt01_E	cor_NE	stat_NE	p_NE	conf.low	conf.high	plt01_NE	plt01
financial.dependency	CORErisk	-0.0062	-0.1355	0.892	-0.09505	0.08283	FALSE	0.095	2.317	0.0208	0.01452	0.1745	FALSE	0
financial.distress	COREnr	0.31	7.235	1.84e-12	0.2298	0.3905	TRUE	0.35	9.15	9.3e-19	0.2803	0.4217	TRUE	2
financial.distress	COREtotal	0.31	7.204	2.25e-12	0.2286	0.3894	TRUE	0.35	9.106	1.32e-18	0.2787	0.4203	TRUE	2
financial.distress	EQ.index	-0.2	-4.57	6.19e-06	-0.2871	-0.1166	TRUE	-0.27	-6.839	2e-11	-0.3446	-0.195	TRUE	2
financial.distress	CORErisk	0.19	4.307	2.01e-05	0.105	0.2763	TRUE	0.21	5.206	2.67e-07	0.1314	0.2858	TRUE	2
financial.distress	WGO.positive	-0.18	-4.094	4.98e-05	-0.2675	-0.09554	TRUE	-0.19	-4.568	5.99e-06	-0.2619	-0.106	TRUE	2
financial.distress	EQ.VAS	-0.18	-3.94	9.36e-05	-0.2611	-0.08871	TRUE	-0.24	-5.893	6.4e-09	-0.3109	-0.1584	TRUE	2
financial.distress	WGO.negative	-0.17	-3.877	0.00012	-0.2585	-0.08595	TRUE	-0.21	-5.107	4.42e-07	-0.2821	-0.1275	TRUE	2
hours.social.networks	WGO.negative	-0.029	-0.6321	0.528	-0.1174	0.06038	FALSE	-0.098	-2.391	0.0171	-0.1774	-0.01755	FALSE	0
hours.social.networks	EQ.VAS	0.02	0.4375	0.662	-0.06918	0.1086	FALSE	-0.15	-3.592	0.000355	-0.2246	-0.06662	TRUE	1
hours.social.networks	WGO.positive	0.018	0.3885	0.698	-0.0714	0.1064	FALSE	-0.096	-2.33	0.0202	-0.175	-0.01502	FALSE	0
hours.social.networks	CORErisk	-0.013	-0.2833	0.777	-0.1017	0.07616	FALSE	0.14	3.417	0.000677	0.05948	0.2178	TRUE	1
hours.social.networks	COREtotal	-0.0033	-0.07265	0.942	-0.09222	0.08567	FALSE	0.22	5.351	1.26e-07	0.1371	0.2911	TRUE	1
hours.social.networks	COREnr	-0.002	-0.04346	0.965	-0.09091	0.08699	FALSE	0.21	5.337	1.35e-07	0.1366	0.2906	TRUE	1
hours.social.networks	EQ.index	0.00039	0.008515	0.993	-0.08856	0.08933	FALSE	-0.14	-3.32	0.000957	-0.214	-0.05553	TRUE	1
number.kids	EQ.VAS	0.059	1.311	0.19	-0.02961	0.1477	FALSE	0.011	0.2726	0.785	-0.06954	0.09188	FALSE	0
number.kids	CORErisk	-0.058	-1.277	0.202	-0.1461	0.03117	FALSE	0.0028	0.06892	0.945	-0.0779	0.08354	FALSE	0
number.kids	EQ.index	0.042	0.924	0.356	-0.04716	0.1304	FALSE	-0.026	-0.6216	0.534	-0.1061	0.05521	FALSE	0
number.kids	COREtotal	-0.039	-0.8682	0.386	-0.1279	0.04969	FALSE	-0.023	-0.5538	0.58	-0.1034	0.058	FALSE	0
number.kids	COREnr	-0.035	-0.78	0.436	-0.124	0.05368	FALSE	-0.025	-0.6087	0.543	-0.1056	0.05574	FALSE	0
number.kids	WGO.positive	-0.017	-0.3656	0.715	-0.1054	0.07244	FALSE	0.019	0.4533	0.651	-0.06212	0.09926	FALSE	0
number.kids	WGO.negative	-0.0092	-0.2033	0.839	-0.09811	0.07977	FALSE	-0.044	-1.056	0.291	-0.1238	0.03733	FALSE	0
routine	COREtotal	-0.31	-7.185	2.56e-12	-0.3887	-0.2278	TRUE	-0.24	-6.035	2.81e-09	-0.3161	-0.164	TRUE	2
routine	COREnr	-0.31	-7.171	2.81e-12	-0.3881	-0.2272	TRUE	-0.25	-6.186	1.16e-09	-0.3215	-0.1698	TRUE	2
routine	WGO.positive	0.21	4.828	1.85e-06	0.1279	0.2976	TRUE	0.15	3.657	0.000278	0.06923	0.2271	TRUE	2
routine	EQ.index	0.21	4.822	1.91e-06	0.1276	0.2974	TRUE	0.15	3.778	0.000174	0.07414	0.2318	TRUE	2

var1	var2	cor_E	stat_E	p_E	conf.low.x	conf.high.x	lt01_E	cor_NE	stat_NE	p_NE	conf.low.y	conf.high.y	lt01_NE	nlt01
routine	CORErisk	-0.21	-4.626	4.8e-06	-0.2894	-0.119	TRUE	-0.11	-2.655	0.00814	-0.1879	-0.02838	TRUE	2
routine	EQ.VAS	0.19	4.354	1.63e-05	0.107	0.2783	TRUE	0.13	3.134	0.00181	0.04796	0.2068	TRUE	2
routine	WGO.negative	0.13	2.994	0.00289	0.04648	0.2212	TRUE	0.16	3.886	0.000113	0.07853	0.236	TRUE	2
sleeping.hours	COREnr	-0.27	-6.248	9.13e-10	-0.3535	-0.1888	TRUE	-0.075	-1.822	0.0689	-0.1547	0.005811	FALSE	1
sleeping.hours	COREtotal	-0.26	-6.043	3.02e-09	-0.3457	-0.1802	TRUE	-0.074	-1.799	0.0725	-0.1538	0.006762	FALSE	1
sleeping.hours	EQ.index	0.21	4.832	1.81e-06	0.128	0.2978	TRUE	0.12	2.94	0.00341	0.04004	0.1992	TRUE	2
sleeping.hours	EQ.VAS	0.19	4.351	1.65e-05	0.1069	0.2782	TRUE	0.091	2.211	0.0274	0.01015	0.1703	FALSE	1
sleeping.hours	WGO.negative	0.12	2.622	0.00901	0.02972	0.2051	TRUE	0.12	2.948	0.00333	0.04034	0.1994	TRUE	2
sleeping.hours	CORErisk	-0.1	-2.31	0.0213	-0.1916	-0.01562	FALSE	-0.039	-0.9549	0.34	-0.1197	0.0415	FALSE	0
sleeping.hours	WGO.positive	0.063	1.391	0.165	-0.026	0.1512	FALSE	0.012	0.2986	0.765	-0.06848	0.09294	FALSE	0

So using the correlations within each of the groups we have 7 numeric variables (of 8) which have a correlation with at least one of the dependent variables significant at $p < .01$ in at least one of the groups. These are in *vecCorrPredictors* and they are: *age*, *exercising.week*, *financial.dependency*, *financial.distress*, *hours.social.networks*, *routine* and *sleeping.hours*.

I generally start with age and gender, treating them a big separately from the other predictors as these seem such fundamental demographic variables. The following shows the relationship between scores on each of the dependents and age pooled across gender and group.

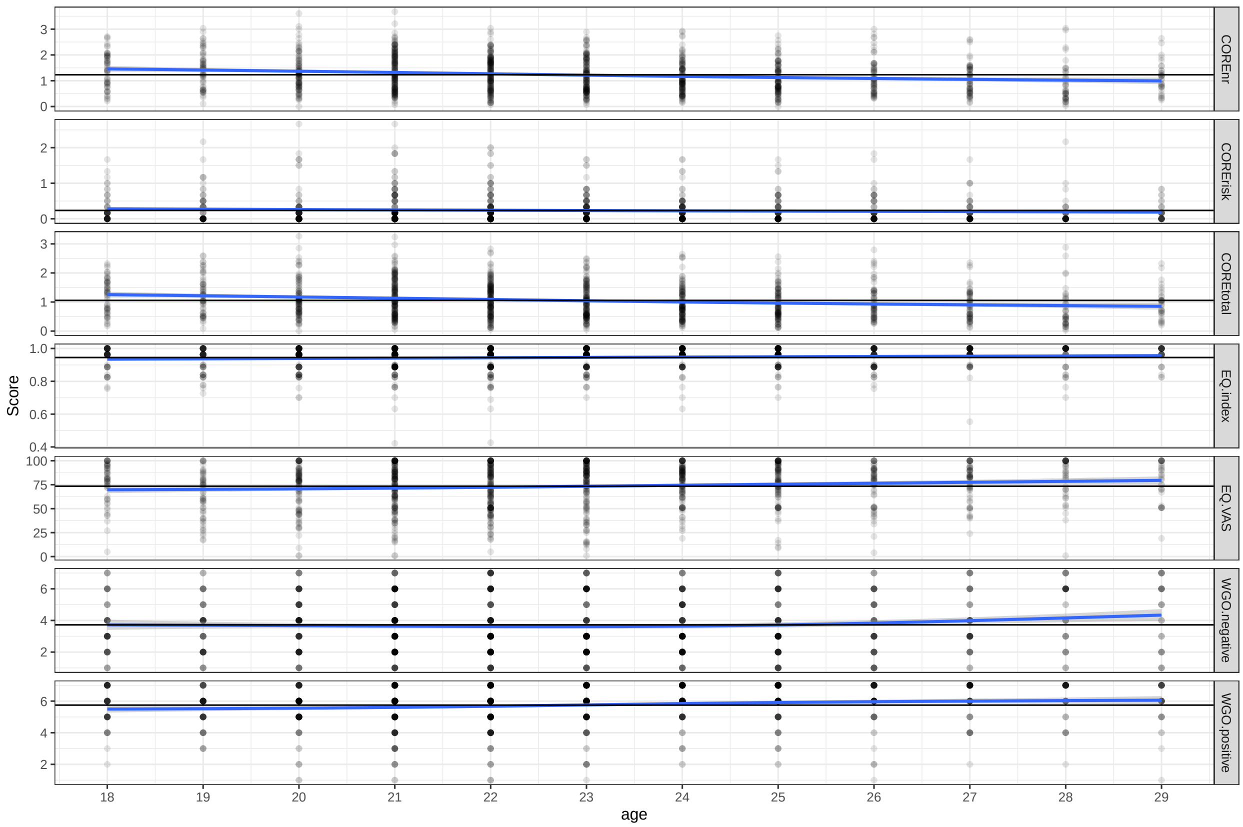

Black reference lines are gender. Now partition by gender.

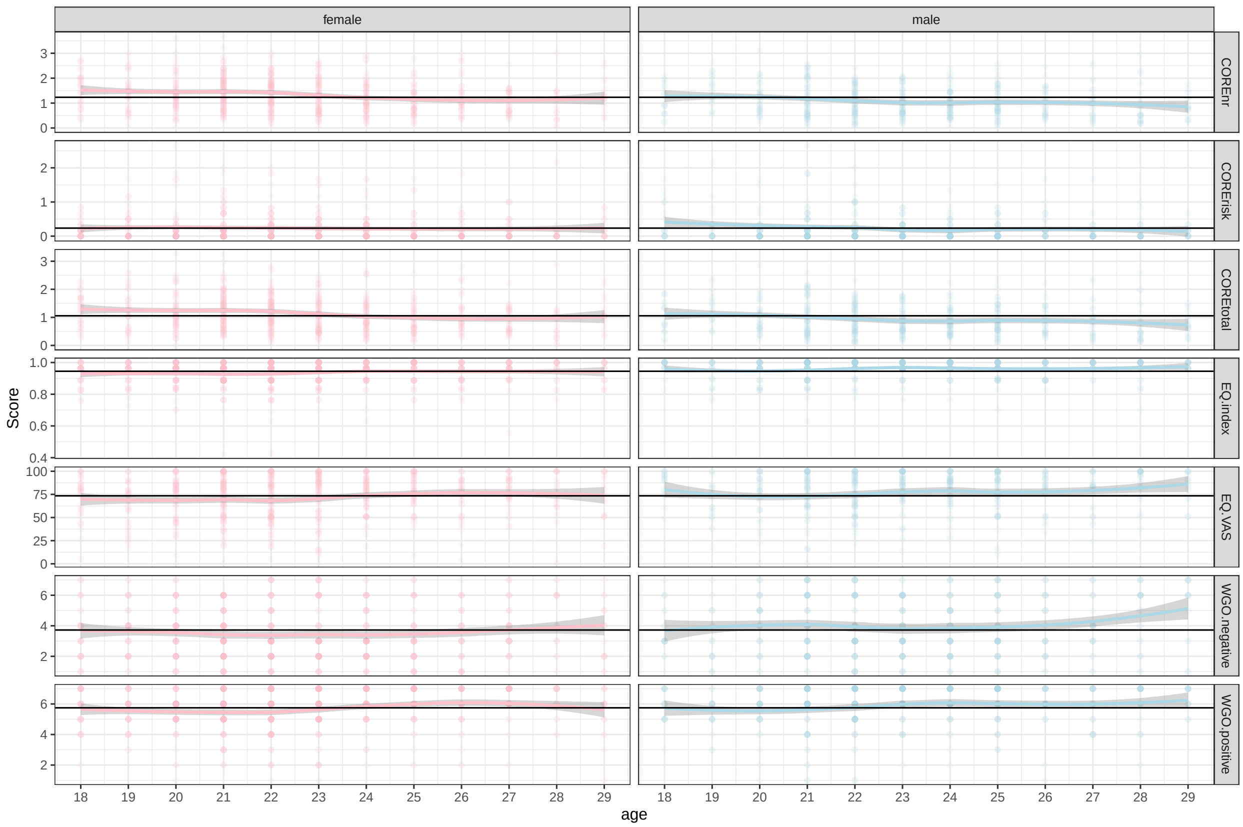

I kept the the black reference lines on the same means there, i.e. ignoring gender. Maybe this is better:

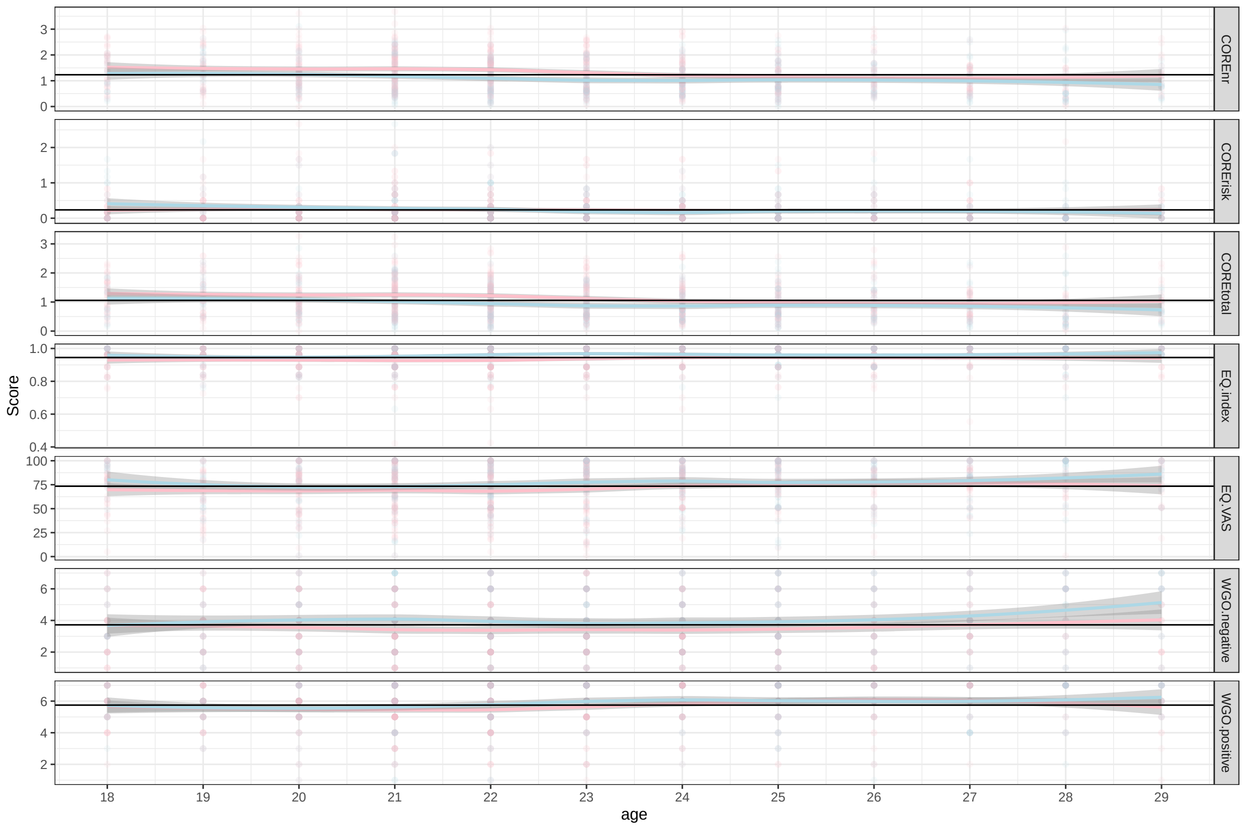

Not really!

By group ignoring gender.

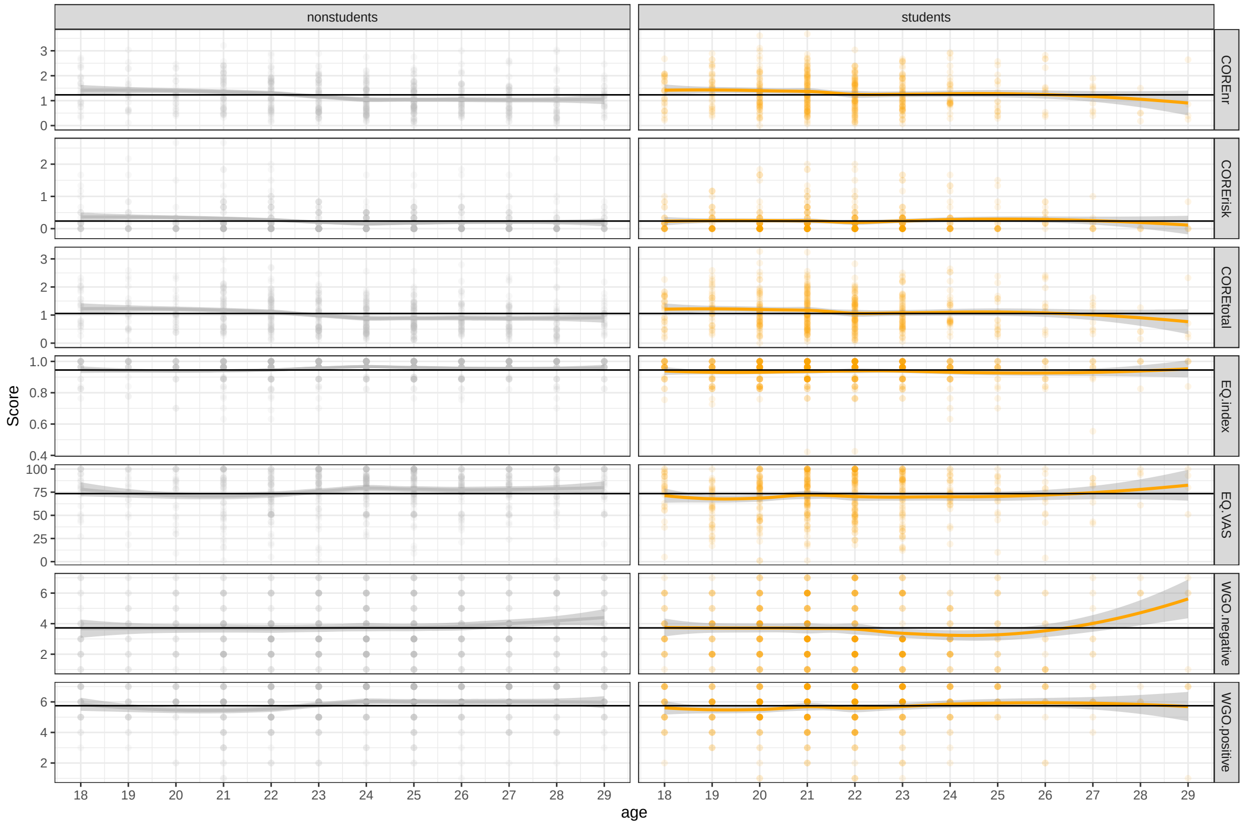

And now by both.

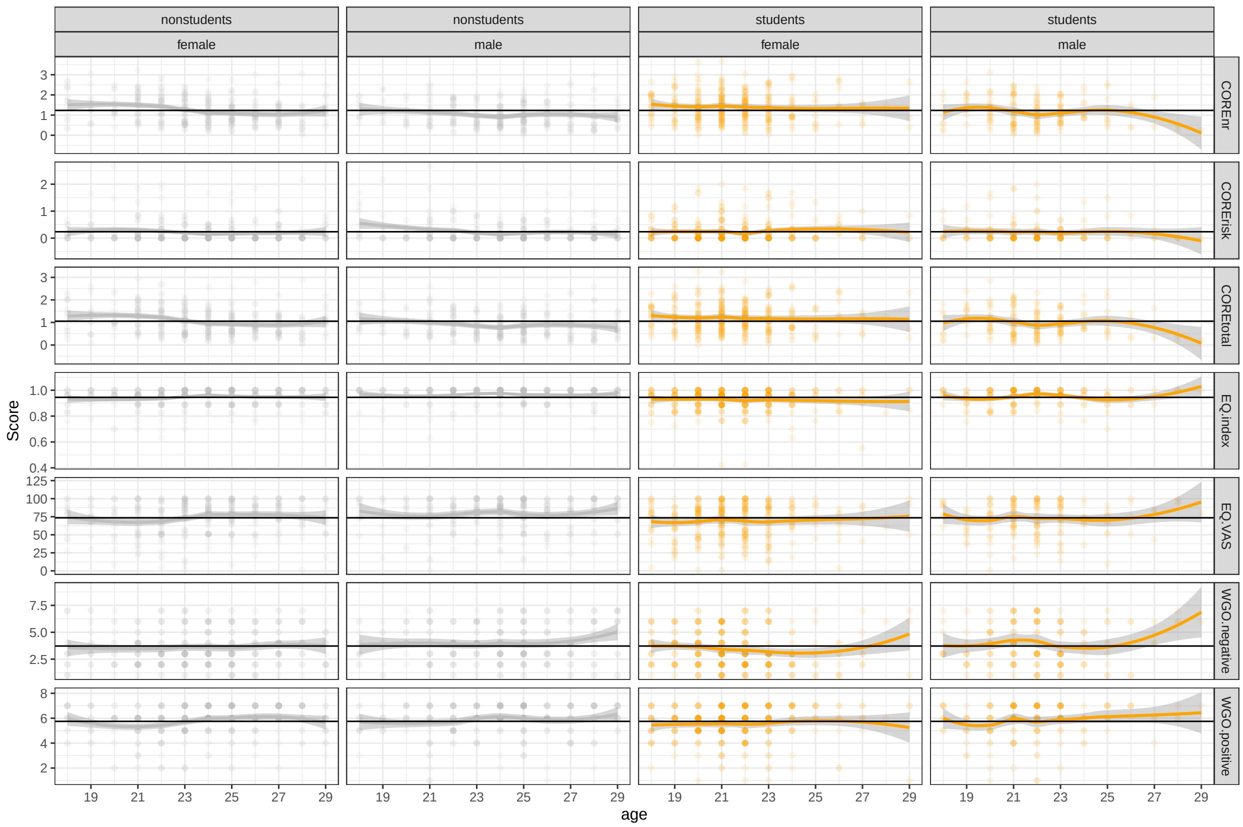

I wouldn't make much of the trumpet bells on the male student's loess smoothed lines. Generally I think we can see small gender differences and that the main feature is a fairly linear decrease in both the *COREnr* and *COREtotal* by age. (Of course, the NR and total scores are going to show almost identical relationships with other things as 28/34 of the total score is the NR score.)

Now to the categorical predictors (including gender again).

This next little table just gives us the mumber of predictor/dependent pairs (84) and how many of those showed a relationship significant at $< .01$.

group	n	nPlt01
nonstudents	84	15
students	84	16

Similar by dependent variable, within the two groups.

group	depVar	n	nPlt01
nonstudents	COREnr	12	2
nonstudents	COREsisk	12	3
nonstudents	COREtotal	12	2
nonstudents	EQ.index	12	3
nonstudents	EQ.VAS	12	2
nonstudents	WGO.negative	12	3
nonstudents	WGO.positive	12	0
students	COREnr	12	3
students	COREsisk	12	2

group	depVar	n	nPlt01
students	COREtotal	12	4
students	EQ.index	12	4
students	EQ.VAS	12	1
students	WGO.negative	12	2
students	WGO.positive	12	0

And the same but by predictor variable.

group	predictorVar	n	nPlt01
nonstudents	care.older.adults	7	1
nonstudents	chronic.disease	7	1
nonstudents	civil.status	7	0
nonstudents	gender	7	5
nonstudents	housing	7	1
nonstudents	infants	7	0
nonstudents	living	7	0
nonstudents	main.caretaker	7	0
nonstudents	mh.diagnosis	7	6
nonstudents	mh.medication	7	1
nonstudents	minors	7	0
nonstudents	paternity	7	0
students	care.older.adults	7	1
students	chronic.disease	7	1
students	civil.status	7	0
students	gender	7	4
students	housing	7	0
students	infants	7	0
students	living	7	0
students	main.caretaker	7	0
students	mh.diagnosis	7	6
students	mh.medication	7	4
students	minors	7	0
students	paternity	7	0

This shows which associations are significant at p < .01 (as "1") within the non-student group.

predictorVar	COREnr	CORErisk	COREtotal	EQ.index	EQ.VAS	WGO.negative	WGO.positive
care.older.adults	0	1	0	0	0	0	0
chronic.disease	0	1	0	0	0	0	0
civil.status	0	0	0	0	0	0	0
gender	1	0	1	1	1	1	0
housing	0	0	0	0	0	1	0
infants	0	0	0	0	0	0	0
living	0	0	0	0	0	0	0
main.caretaker	0	0	0	0	0	0	0
mh.diagnosis	1	1	1	1	1	1	0
mh.medication	0	0	0	1	0	0	0
minors	0	0	0	0	0	0	0
paternity	0	0	0	0	0	0	0

Same within the student group.i

predictorVar	COREnr	COREristk	COREtotal	EQ.index	EQ.VAS	WGO.negative	WGO.positive
care.older.adults	0	0	1	0	0	0	0
chronic.disease	0	0	0	1	0	0	0
civil.status	0	0	0	0	0	0	0
gender	1	0	1	1	0	1	0
housing	0	0	0	0	0	0	0
infants	0	0	0	0	0	0	0
living	0	0	0	0	0	0	0
main.caretaker	0	0	0	0	0	0	0
mh.diagnosis	1	1	1	1	1	1	0
mh.medication	1	1	1	1	0	0	0
minors	0	0	0	0	0	0	0
paternity	0	0	0	0	0	0	0

Summed across the two groups, i.e. 2 means that both groups showed a statistically significant predictor/dependent relationship at p < .01.

predictorVar	COREnr	COREristk	COREtotal	EQ.index	EQ.VAS	WGO.negative	WGO.positive
care.older.adults	0	1	1	0	0	0	0
chronic.disease	0	1	0	1	0	0	0
civil.status	0	0	0	0	0	0	0
gender	2	0	2	2	1	2	0
housing	0	0	0	0	0	1	0
infants	0	0	0	0	0	0	0
living	0	0	0	0	0	0	0
main.caretaker	0	0	0	0	0	0	0
mh.diagnosis	2	2	2	2	2	2	0
mh.medication	1	1	1	2	0	0	0
minors	0	0	0	0	0	0	0
paternity	0	0	0	0	0	0	0

I haven't really tried to summarise that but it is clear that some predictors, e.g. *financial.distress* (it's "distress"!) have relationships with many of the dependents and in both groups. Interesting that *gender* has impacts too and unsurprising (validating?!) that *mh.diagnosis* has a lot of statistically significant associations too.

What about the effect sizes?

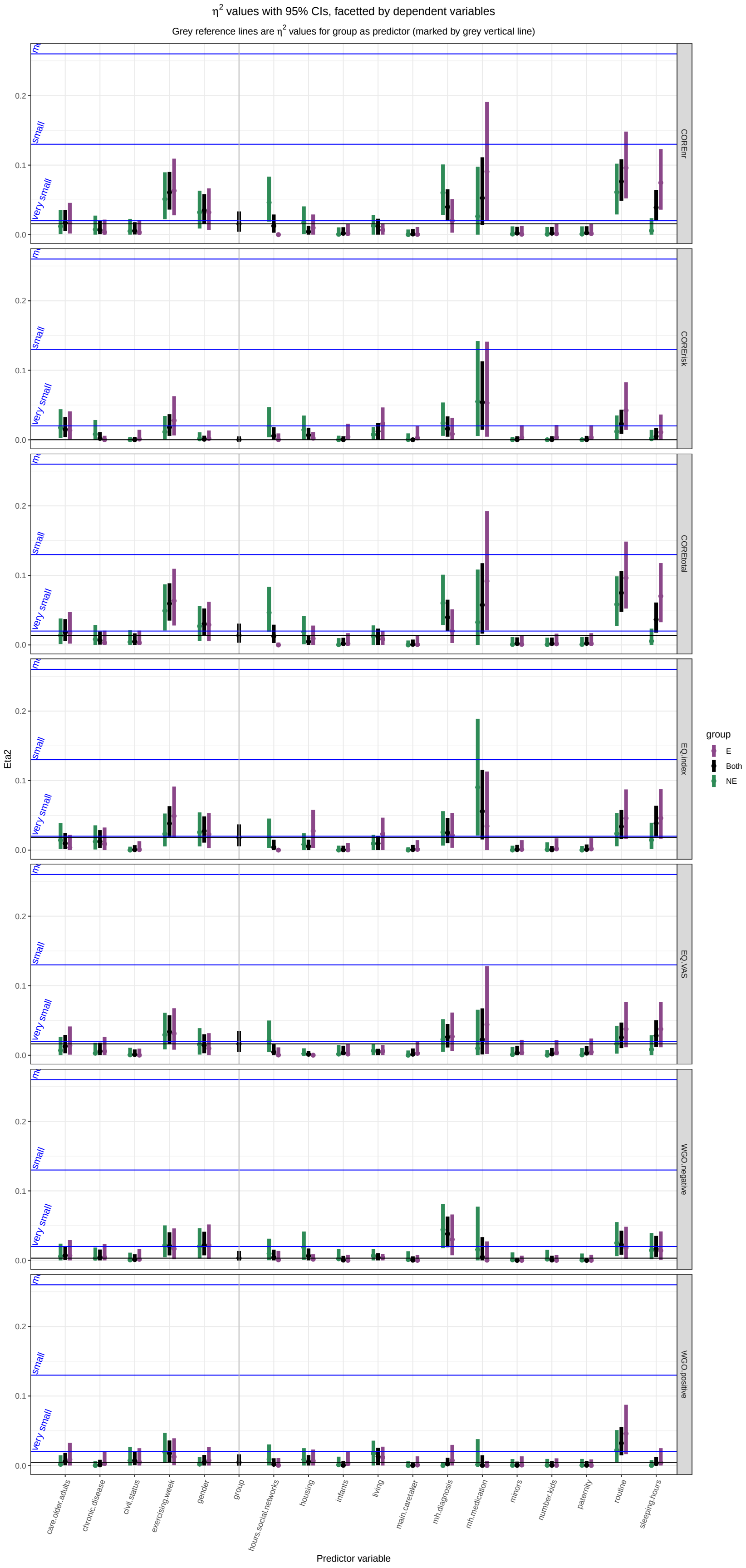
η² values with 95% CIs, facetted by dependent variables
Grey reference lines are η² values for group as predictor (marked by grey vertical line)
small
very small
COREnr
COREtisk
COREtotal
EQ.index
EQ.VAS
WGO.negative
WGO.positive
Eta2
group
E
Both
NE
care.older.adults
chronic.disease
civil.status
exercising.week
gender
group
hours.social.networks
housing
infants
living
main.caretaker
mh.diagnosis
mh.medication
minors
number.kids
paternity
routine
sleeping.hours
Predictor variable

Hm. I will leave that there but it's pretty indigestible so I'll go through the dependent variables one by one. I have recomputed the ordering of the predictors to that of the η^2 for *group* == "both" for the focal dependent variable and that means that the effect of group will be in a different point on the x axis for the different dependent variables.

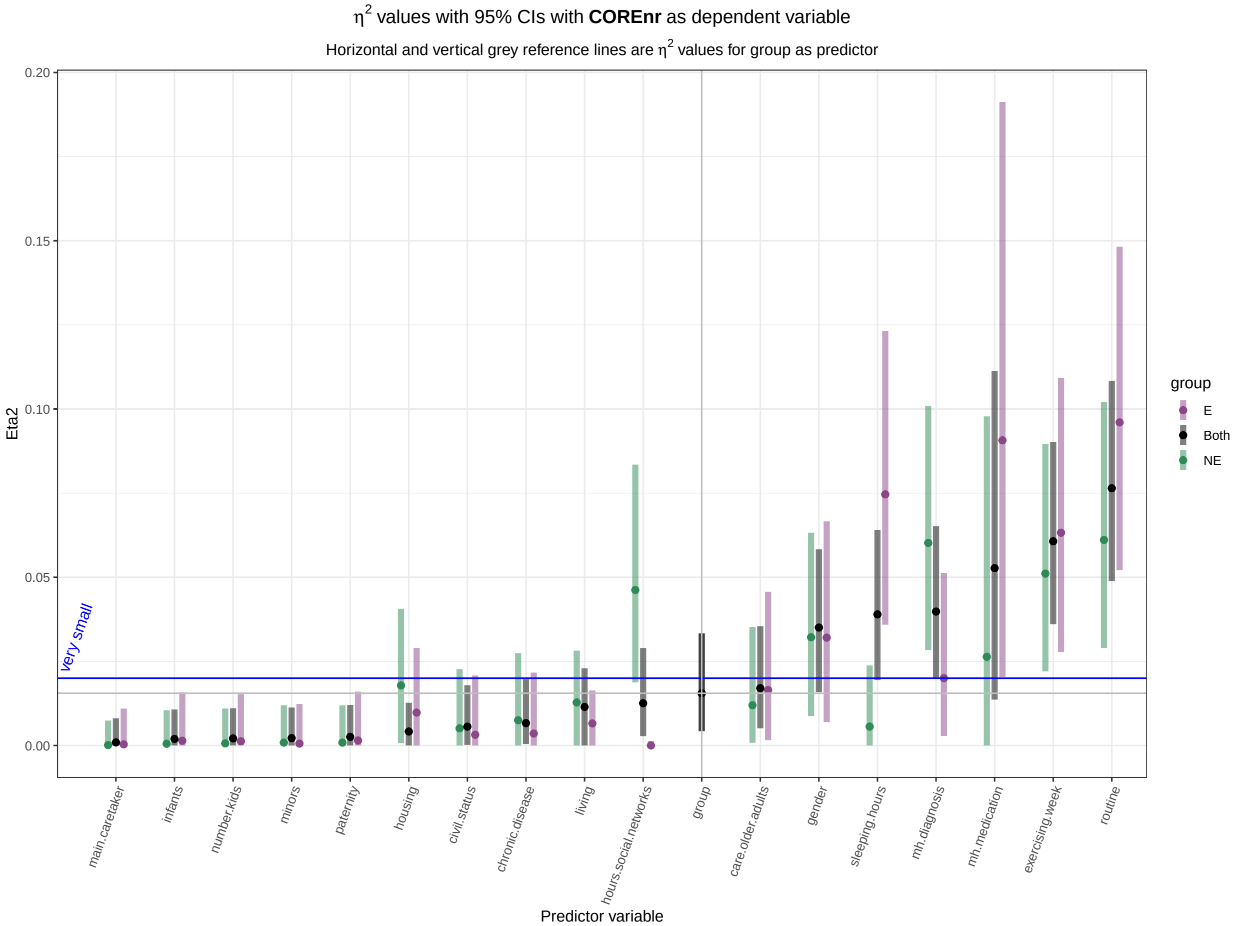

COREnr

So for COREnr as dependent, there are a number of predictors with greater effect than that of *group* and some predictors have rather different effect sizes depending on whether the group is the students, nonstudents or the pooled complete tibBaseline dataset. None of the η^2 values is over into Cohen's "small" effect size range.

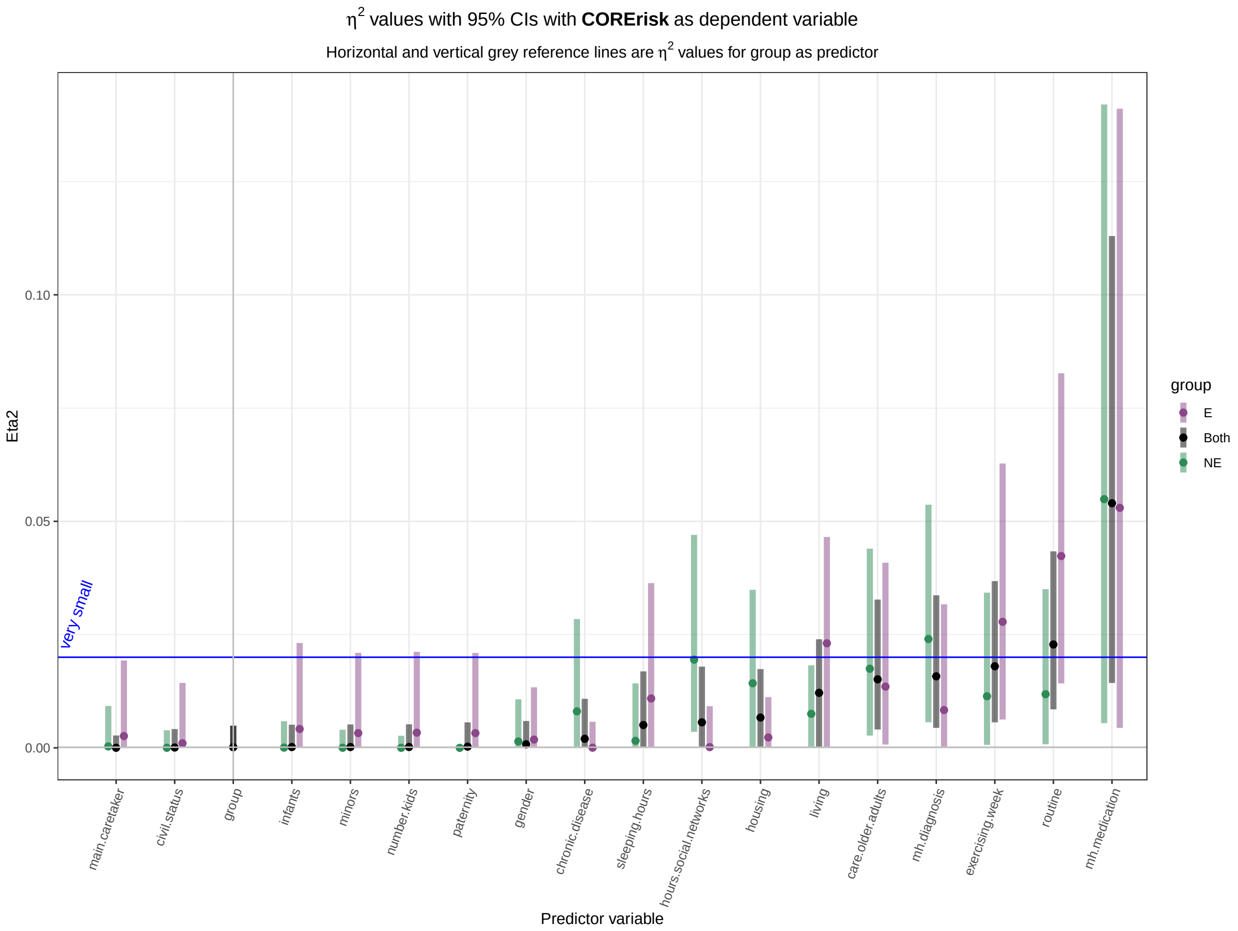

CORErisk

Rather different pattern, smaller effect sizes. None of the η^2 values into the "small" range.

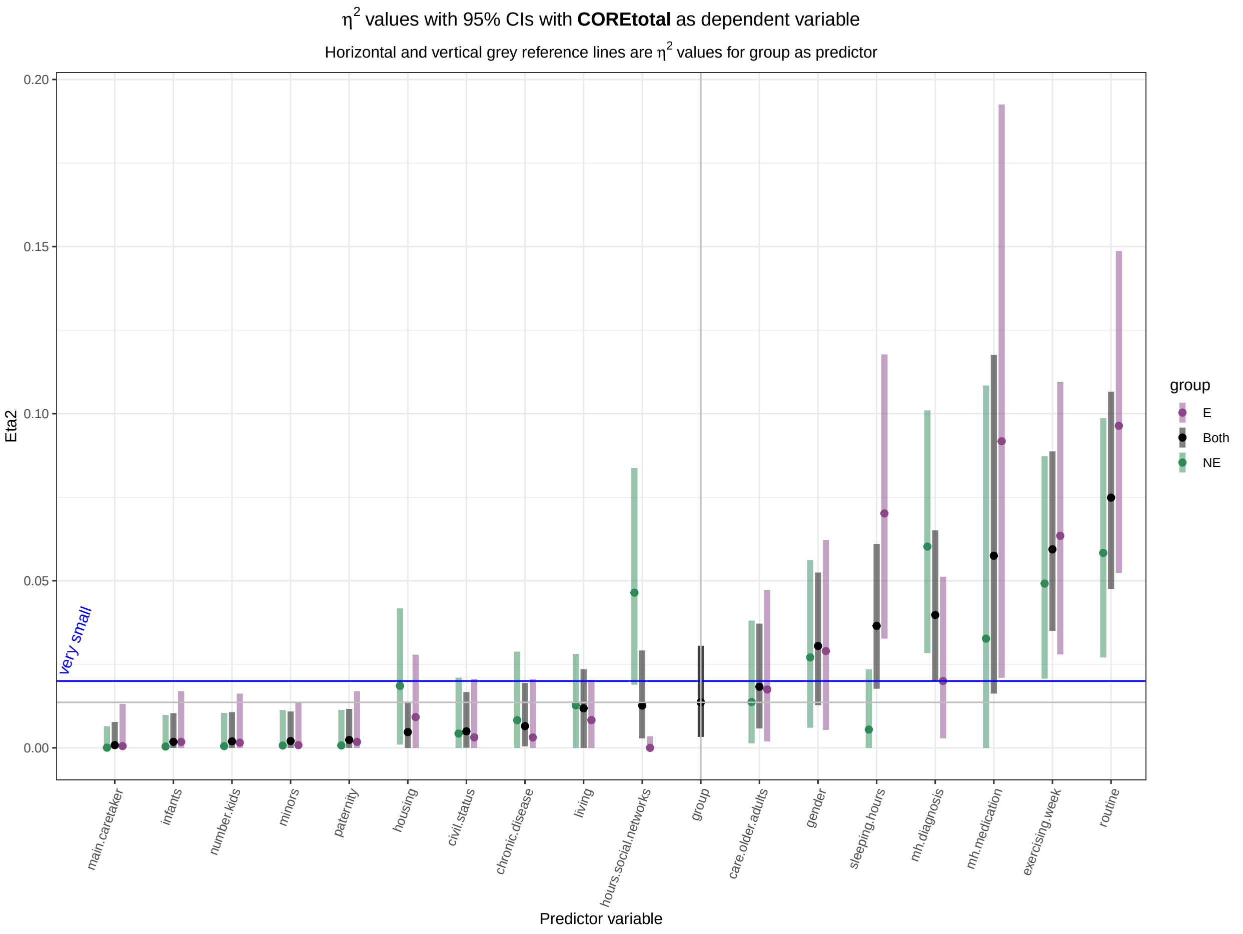

COREtotal

Of course, very similar to the COREnr picture. None η^2 in the “small” range.

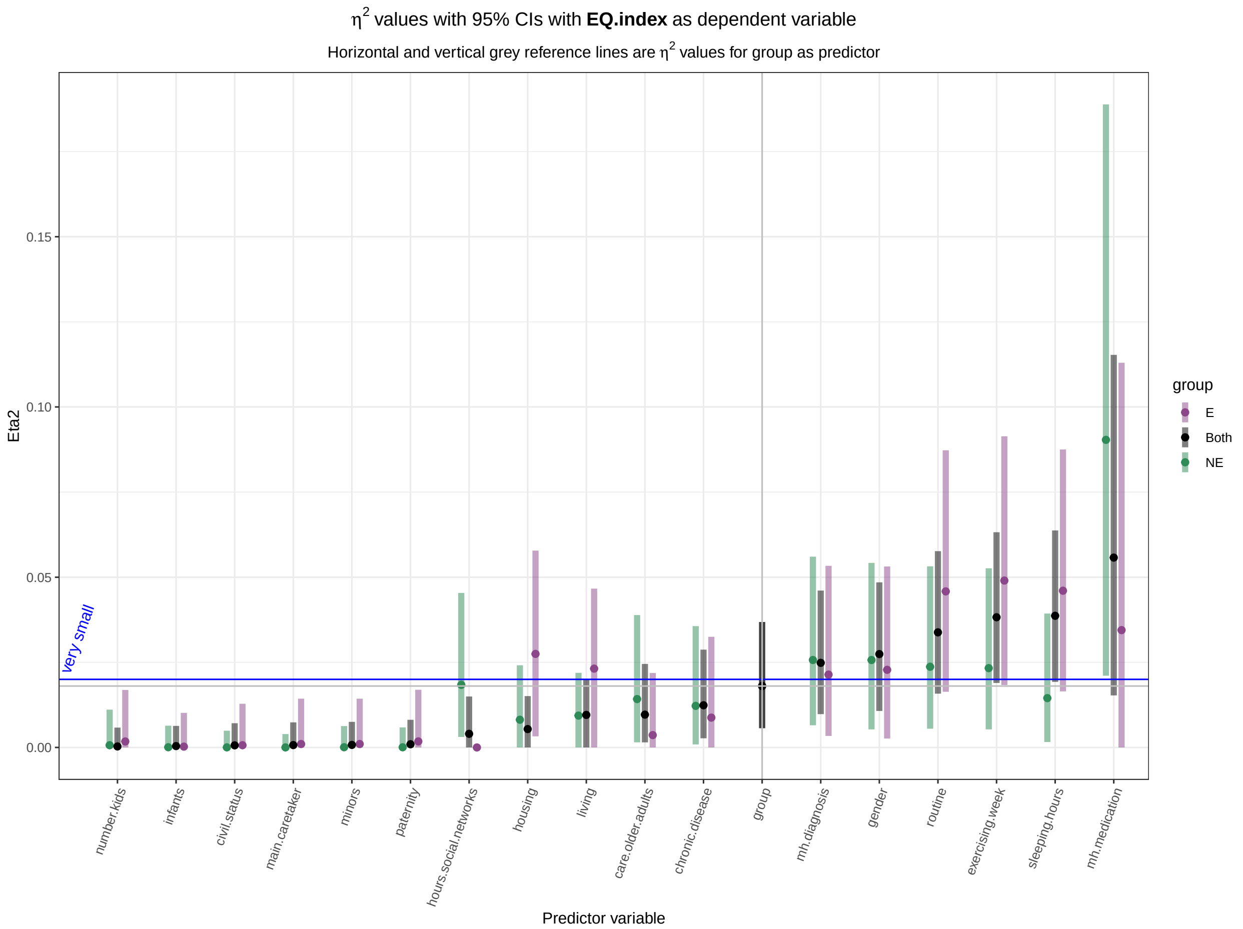

EQ.index

All very small or tiny effects, *civil.status* and, to a lesser extent, *housing* have markedly different, but still tiny, effects in the two groups. *Civil.status* has a markedly different effect on *EQ.index* in the two groups.

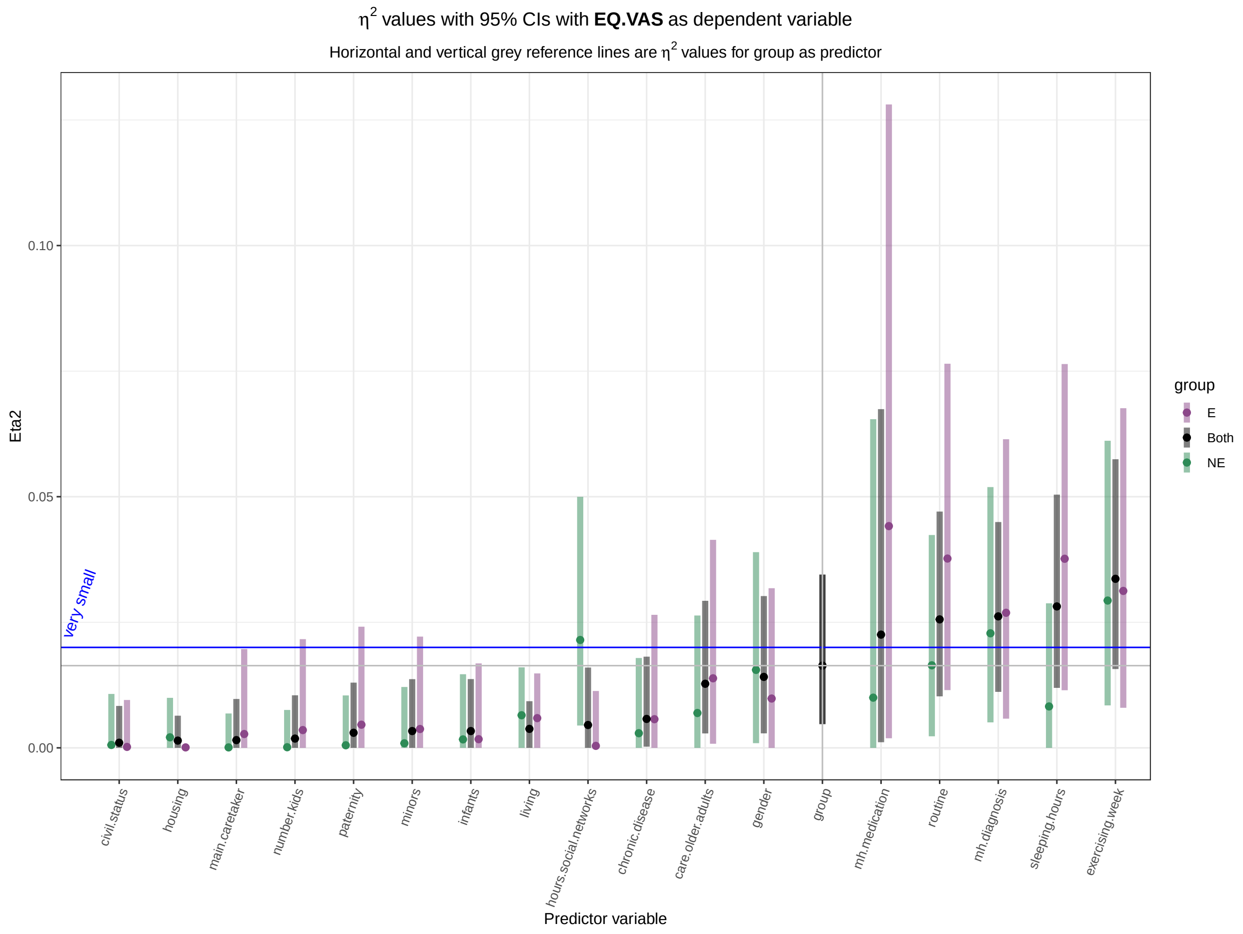

EQ.VAS

Again all very small or tiny effects, but the pattern is rather different from that for *EQ.index*.

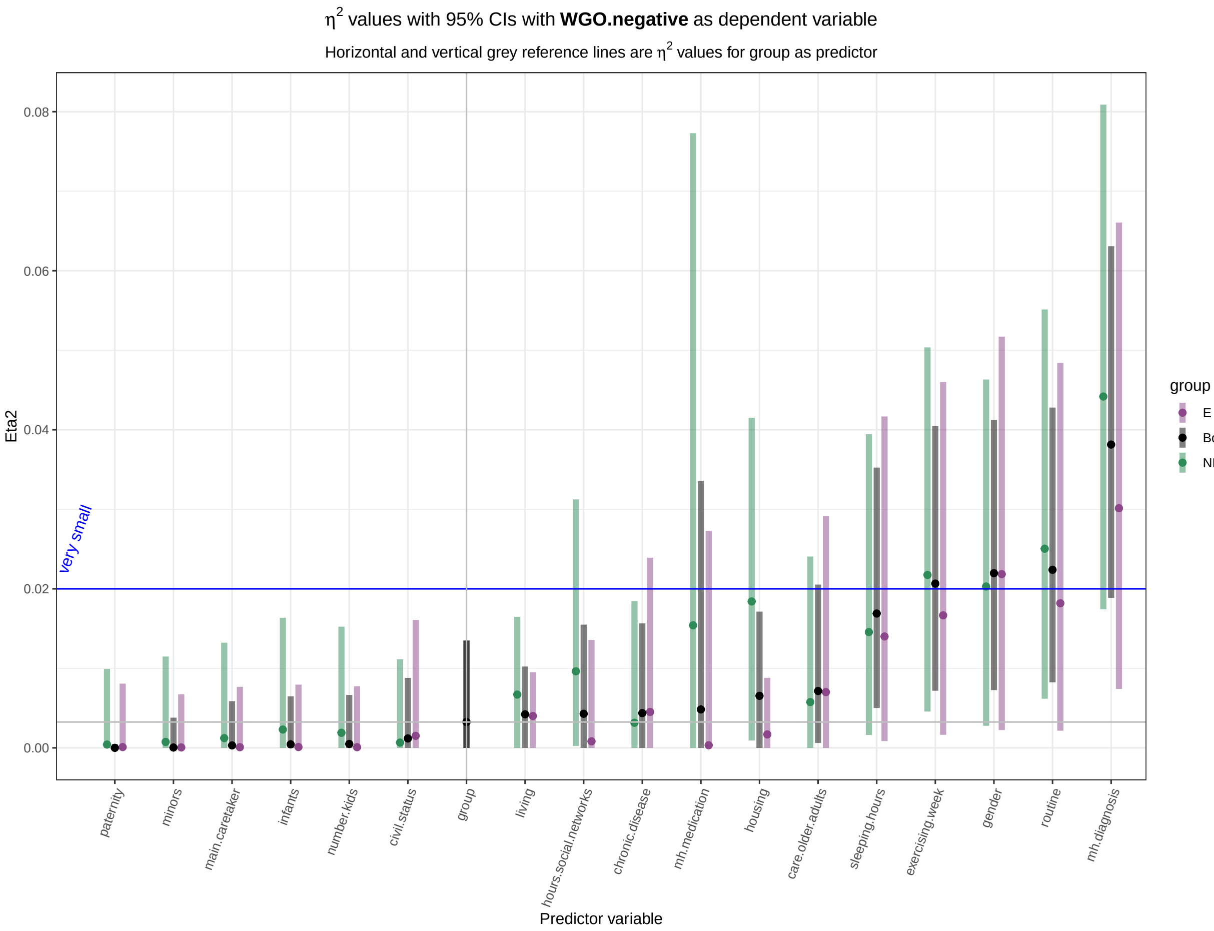

WGO.negative

All tiny effects, *civil.status* and, to a lesser extent, *housing* have markedly different, but still tiny, effects in the two groups.

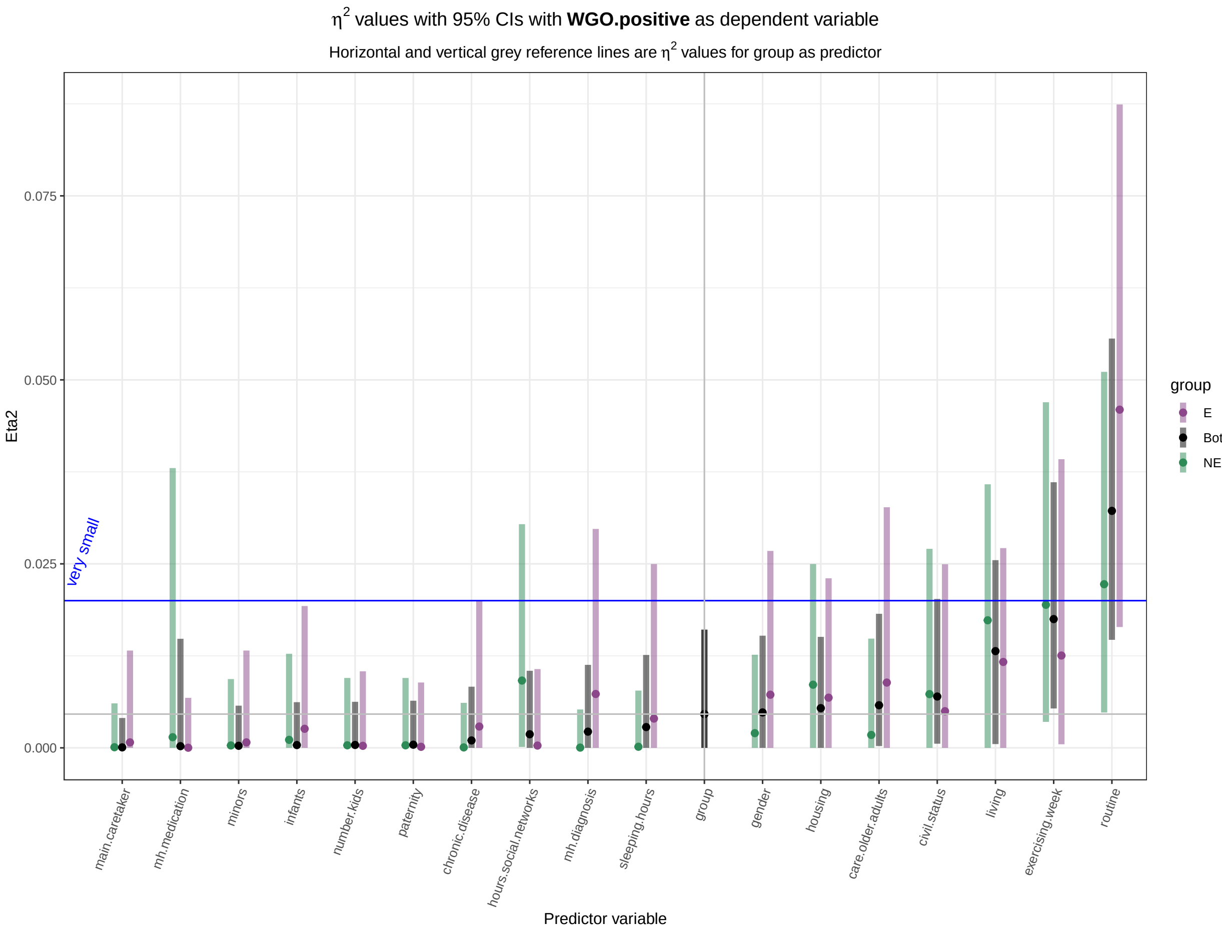

WGO.positive

Again, all tiny or very small effects.

Within group relationships betweeen predictors

This is really only an appendix here but I have included it as it provides contextual information I guess.

Starting with the numeric predictors. I've only kept those with p < .01. First within the non-student group.

var1	var2	cor	statistic	p	conf.low	conf.high	Eta2	Cohen1992
age	financial.dependency	-0.38	-10.05	4.91e-22	-0.4497	-0.3118	0.1444	medium
age	hours.social.networks	-0.16	-3.839	0.000137	-0.2341	-0.07662	0.0256	small
age	sleeping.hours	-0.12	-2.935	0.00347	-0.1989	-0.03982	0.0144	very small
financial.dependency	hours.social.networks	0.19	4.66	3.92e-06	0.1097	0.2654	0.0361	small
financial.dependency	sleeping.hours	0.18	4.38	1.41e-05	0.09843	0.2548	0.0324	small
financial.distress	exercising.week	-0.14	-3.305	0.00101	-0.2134	-0.05493	0.0196	very small
financial.distress	sleeping.hours	-0.12	-2.933	0.00348	-0.1989	-0.03976	0.0144	very small

var1	var2	cor	statistic	p	conf.low	conf.high	Eta2	Cohen1992
routine	exercising.week	0.33	8.574	8.84e-17	0.2596	0.4032	0.1089	small
routine	hours.social.networks	-0.18	-4.42	1.18e-05	-0.2563	-0.1	0.0324	small
sleeping.hours	hours.social.networks	0.15	3.556	0.000406	0.06515	0.2232	0.0225	small

Now the students.

var1	var2	cor	statistic	p	conf.low	conf.high	Eta2	Cohen1992
age	financial.dependency	-0.27	-6.224	1.05e-09	-0.3526	-0.1878	0.0729	small
age	financial.distress	0.16	3.623	0.000322	0.07462	0.2479	0.0256	small
financial.distress	sleeping.hours	-0.18	-3.951	8.94e-05	-0.2616	-0.08922	0.0324	small
financial.distress	routine	-0.14	-3.222	0.00136	-0.2309	-0.05671	0.0196	very small
routine	exercising.week	0.31	7.283	1.34e-12	0.2318	0.3922	0.0961	small
routine	hours.social.networks	-0.12	-2.624	0.00897	-0.2052	-0.0298	0.0144	very small
sleeping.hours	routine	0.24	5.412	9.81e-08	0.1532	0.321	0.0576	small
sleeping.hours	exercising.week	0.12	2.614	0.00923	0.02935	0.2048	0.0144	very small

Hm. OK, makes sense that there would be different associations within each group. Bring both together.

var1	var2	cor	statistic	p	Eta2	Cohen1992	group	inBoth
age	financial.dependency	-0.38	-10.05	4.91e-22	0.1444	medium	nonstudents	Y
age	financial.dependency	-0.27	-6.224	1.05e-09	0.0729	small	students	Y
age	financial.distress	0.16	3.623	0.000322	0.0256	small	students	N
age	hours.social.networks	-0.16	-3.839	0.000137	0.0256	small	nonstudents	N
age	sleeping.hours	-0.12	-2.935	0.00347	0.0144	very small	nonstudents	N
financial.dependency	hours.social.networks	0.19	4.66	3.92e-06	0.0361	small	nonstudents	N
financial.dependency	sleeping.hours	0.18	4.38	1.41e-05	0.0324	small	nonstudents	N
financial.distress	exercising.week	-0.14	-3.305	0.00101	0.0196	very small	nonstudents	N
financial.distress	routine	-0.14	-3.222	0.00136	0.0196	very small	students	N
financial.distress	sleeping.hours	-0.12	-2.933	0.00348	0.0144	very small	nonstudents	Y
financial.distress	sleeping.hours	-0.18	-3.951	8.94e-05	0.0324	small	students	Y

var1	var2	cor	statistic	p	Eta2	Cohen1992	group	inBoth
routine	exercising.week	0.33	8.574	8.84e-17	0.1089	small	nonstudents	Y
routine	exercising.week	0.31	7.283	1.34e-12	0.0961	small	students	Y
routine	hours.social.networks	-0.18	-4.42	1.18e-05	0.0324	small	nonstudents	Y
routine	hours.social.networks	-0.12	-2.624	0.00897	0.0144	very small	students	Y
sleeping.hours	exercising.week	0.12	2.614	0.00923	0.0144	very small	students	N
sleeping.hours	hours.social.networks	0.15	3.556	0.000406	0.0225	small	nonstudents	N
sleeping.hours	routine	0.24	5.412	9.81e-08	0.0576	small	students	N

So we have 14 pairs of numeric predictors with a correlation significant at p < .01 in at least one of the groups. The correlation between *age* and *financial.dependency* is the strongest at -0.38 with a medium effect size in nonstudents, all the other effect sizes are small or very small even though they are statistically significant.

Now the character variables.

First the nonstudent group.

V1	V2	n	statistic	p	df
living	civil.status	590	225.2	1.16e-46	5
living	minors	590	161.4	4.88e-33	5
living	paternity	590	158	2.64e-32	5
living	infants	590	140.8	1.18e-28	5
living	main.caretaker	590	139	2.89e-28	5
living	housing	590	150.1	2.34e-24	15
housing	civil.status	590	55.93	4.35e-12	3
housing	minors	590	54.97	6.97e-12	3
housing	paternity	590	53.13	1.72e-11	3
housing	infants	590	50.75	5.54e-11	3
housing	main.caretaker	590	38.21	2.55e-08	3
employment	living	590	45.51	1.76e-06	10
gender	living	590	38.35	3.29e-05	10
employment	main.caretaker	590	19.63	5.47e-05	2
gender	main.caretaker	590	18.44	9.93e-05	2
employment	minors	590	13.36	0.00126	2
employment	civil.status	590	12.17	0.00228	2
employment	paternity	590	12.07	0.00239	2
employment	chronic.disease	590	12.05	0.00241	2
employment	infants	590	10.5	0.00524	2

V1	V2	n	statistic	p	df
living	civil.status	486	133.5	4.32e-27	5
living	main.caretaker	486	81.37	4.34e-16	5
living	minors	486	81.37	4.34e-16	5
living	paternity	486	73.36	2.04e-14	5
living	infants	486	64.68	1.31e-12	5

V1	V2	n	statistic	p	df
living	housing	486	61.58	1.34e-07	15
employment	living	486	33.93	2.45e-06	5
housing	mh.diagnosis	486	16.85	0.000758	3
living	care.older.adults	486	15.31	0.00912	5

OK, combine findings from each group.

var1	var2	n	statistic	p	df	group	inBoth
employment	chronic.disease	590	12.05	0.00241	2	nonstudents	N
employment	civil.status	590	12.17	0.00228	2	nonstudents	N
employment	infants	590	10.5	0.00524	2	nonstudents	N
employment	living	590	45.51	1.76e-06	10	nonstudents	Y
employment	living	486	33.93	2.45e-06	5	students	Y
employment	main.caretaker	590	19.63	5.47e-05	2	nonstudents	N
employment	minors	590	13.36	0.00126	2	nonstudents	N
employment	paternity	590	12.07	0.00239	2	nonstudents	N
gender	living	590	38.35	3.29e-05	10	nonstudents	N
gender	main.caretaker	590	18.44	9.93e-05	2	nonstudents	N
housing	civil.status	590	55.93	4.35e-12	3	nonstudents	N
housing	infants	590	50.75	5.54e-11	3	nonstudents	N
housing	main.caretaker	590	38.21	2.55e-08	3	nonstudents	N
housing	mh.diagnosis	486	16.85	0.000758	3	students	N
housing	minors	590	54.97	6.97e-12	3	nonstudents	N
housing	paternity	590	53.13	1.72e-11	3	nonstudents	N
living	care.older.adults	486	15.31	0.00912	5	students	N
living	civil.status	590	225.2	1.16e-46	5	nonstudents	Y
living	civil.status	486	133.5	4.32e-27	5	students	Y
living	housing	590	150.1	2.34e-24	15	nonstudents	Y
living	housing	486	61.58	1.34e-07	15	students	Y
living	infants	590	140.8	1.18e-28	5	nonstudents	Y
living	infants	486	64.68	1.31e-12	5	students	Y
living	main.caretaker	590	139	2.89e-28	5	nonstudents	Y
living	main.caretaker	486	81.37	4.34e-16	5	students	Y
living	minors	590	161.4	4.88e-33	5	nonstudents	Y
living	minors	486	81.37	4.34e-16	5	students	Y
living	paternity	590	158	2.64e-32	5	nonstudents	Y
living	paternity	486	73.36	2.04e-14	5	students	Y

Wow, we have 22 pairs of categorical predictor variables that are statistically significantly associated at $p < .01$ and a total of 36 pairs of variables across the categorical and numeric. Aargh, what about the numeric with the categorical?!

group	catPred	numPred	n	statistic	df	p
nonstudents	civil.status	number.kids	590	92.92	1	5.43e-22
nonstudents	civil.status	age	590	31.47	1	2.02e-08
nonstudents	civil.status	financial.dependency	590	12.65	1	0.000375
nonstudents	civil.status	financial.distress	590	7.774	1	0.0053
nonstudents	gender	exercising.week	590	19.06	2	7.26e-05
nonstudents	gender	financial.distress	590	16.21	2	0.000302

group	catPred	numPred	n	statistic	df	p
nonstudents	gender	financial.dependency	590	10.61	2	0.00497
nonstudents	housing	number.kids	590	53.13	3	1.72e-11
nonstudents	housing	financial.dependency	590	21.53	3	8.18e-05
nonstudents	living	number.kids	590	158.5	5	2.05e-32
nonstudents	living	financial.dependency	590	66.84	5	4.65e-13
nonstudents	living	age	590	40.04	5	1.47e-07
nonstudents	living	financial.distress	590	19.13	5	0.00182
nonstudents	mh.diagnosis	hours.social.networks	590	7.475	1	0.00626
students	care.older.adults	financial.distress	486	10.74	1	0.00105
students	care.older.adults	age	486	7.214	1	0.00723
students	chronic.disease	financial.distress	486	7.93	1	0.00486
students	civil.status	number.kids	486	16.4	1	5.12e-05
students	civil.status	age	486	11.58	1	0.000665
students	gender	financial.distress	486	9.422	2	0.009
students	housing	financial.distress	486	22.39	3	5.41e-05
students	living	number.kids	486	72.89	5	2.56e-14
students	living	financial.dependency	486	37.48	5	4.8e-07
students	living	age	486	21.76	5	0.000583
students	mh.diagnosis	financial.distress	486	13.93	1	0.00019
students	mh.diagnosis	age	486	12.02	1	0.000527
students	mh.medication	routine	486	7.333	1	0.00677

And we have a further 21 effects of categorical predictors on numerical dependents significant at $p < .01$ in one or both groups.

Cumulative summary of what we have so far

Bearing in mind that the pertinent clause in the DAP is this:

> where bivariate associations between tibBaseline variables (including students/non-student group) are statistically significant at $p < .01$, the joint relationship of both variables and the dependent variables will be reported with the interaction terms as an exploration of these complexities

- Group differences on dependent variables:
  - CORE total and NR scores have significant relationships with group at $p < .01$ but not the risk score
  - EQ.VAS and EQ.index both have significant relationshipp with group at $p < .01$
  - neither WGO score is significantly related to group (though WGO.positive nearly is!)
  - all effect sizes are very small.
- Associations between predictors and group:
  - Ten categorical variables, *minors*, *paternity*, *living*, *infants*, *main.caretaker*, *housing*, *care.older.adults*, *civil.status*, *gender* and *chronic.disease* are associated with group with $p < .01$. Only the two mental health questions are *not* associated with group at $p < .01$.
  - Four numeric predictor variables are associated with group: *financial.dependency*, *age*, *sleeping.hours* and *exercising.week*
- Statistically significant ($p < .01$) associations between predictors and dependents:

- Six categorical predictors have an association with at least one dependent variable in at least one of the two groups that is statistically significant at p < .01. These variables are *care.older.adults*, *chronic.disease*, *gender*, *housing*, *mh.diagnosis* and *mh.medication*.
- Seven numerical predictors have an association with at least one dependent variable in at least one of the two groups that is statistically significant at p < .01. These variables are *age*, *exercising.week*, *financial.dependency*, *financial.distress*, *hours.social.networks*, *routine* and *sleeping.hours*.
- However, none of these relationships has an effect size that is more than small.

* Statistically significant associations between predictors other than group:
  - There are 22 of pairs of categorical predictors significant at p < .01 in one or both groups.
  - There are 14 statistically significant correlations (at p < .01) between numeric predictor variables in one or both groups.
  - There are 21 pairs of a categorical predictor with a statistically significantly effect on a numeric predictor at p < .01.

That is really the "interconnectedness of all things" (to quote Dirk Gently from Adams, D. (1987). Dirk Gently's Holistic Detective Agency. Heinemann.) and I think the DAP committed us to look at all that. What matters now is how these associations impact on group differences on the dependent variables:
+ all ten categorical variables: *minors*, *paternity*, *living*, *infants*, *main.caretaker*, *housing*, *care.older.adults*, *civil.status*, *gender* and *chronic.disease* that are associated with group with p < .01. + Four numeric predictor variables are associated with group: *financial.dependency*, *age*, *sleeping.hours* and *exercising.week*

Do categorical predictors associated with group affect how group affects scores on dependent variables?

catPred	depVar	r.squared	adj.r.squared	p.valueFit	nobs	term	estimate	std.error	statistic	p.value
housing	COREtotal	0.027	0.021	1e-04	1076	groupstudents:predVarRenting	0.36	0.16	2.2	0.029
housing	COREnr	0.029	0.023	4.5e-05	1076	groupstudents:predVarRenting	0.41	0.19	2.2	0.028

I think what that is telling us is that though we tested out all ten categorical predictors: *minors*, *paternity*, *living*, *infants*, *main.caretaker*, *housing*, *care.older.adults*, *civil.status*, *gender* and *chronic.disease* for interactions with group in their relationship with all seven (*EQ.VAS*, *EQ.index*, *COREtotal*, *CORErisk*, *COREnr*, *WGO.positive* and *WGO.negative*), i.e. 70 possible interactions, only 2 showed a statistically significant (at p < .05 this time) interaction with group in impact on any of the dependent variables and the specifics were that it was just one variable housing which had two statistically significant interactions with group in impacts on *COREtotal* and *COREnr*.

Here's a plot of the effects.

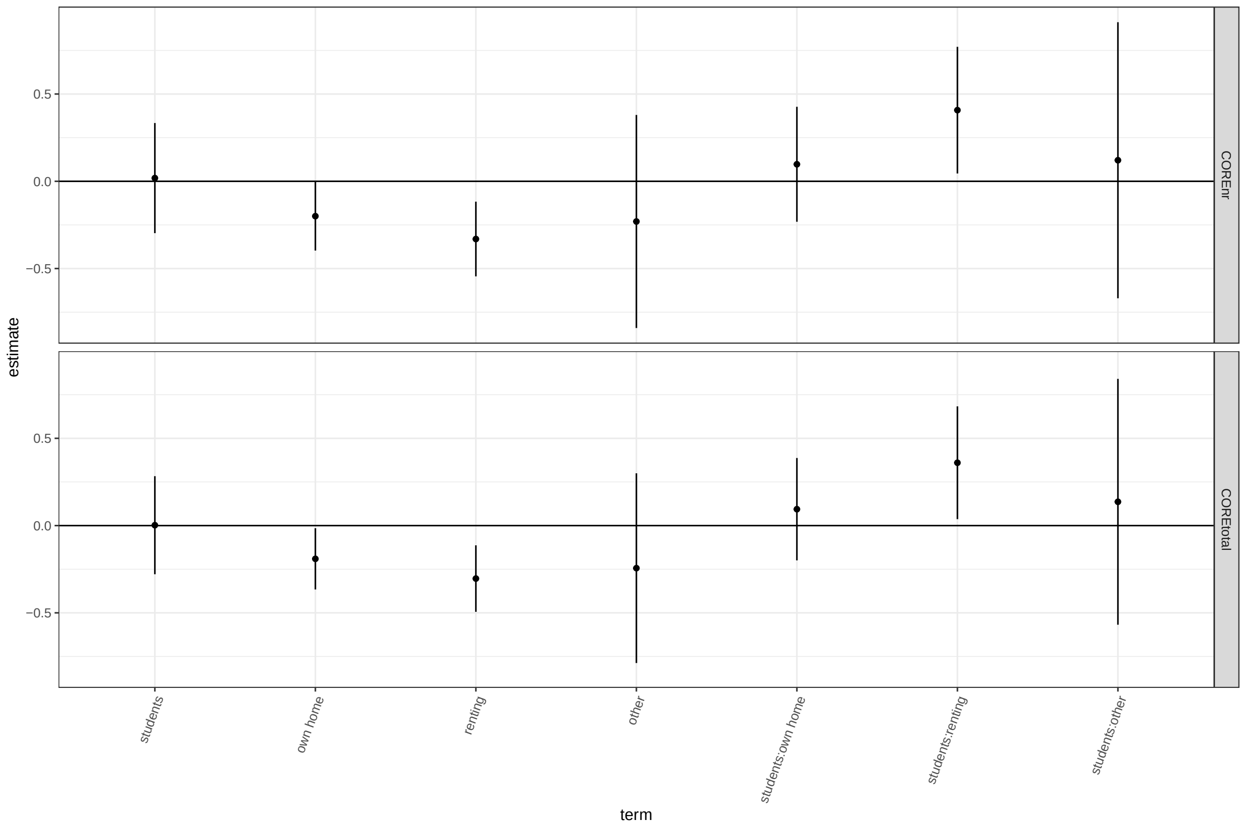

I've omitted the intercept, i.e. the mean for the reference class, the non-students, so that is represented by the horizontal zero reference line. It looks as if there is a tiny (< .5 on the CORE NR and CORE total scores) interaction effect that means that the students who are renting their accommodation score higher, worse, on those CORE scores. Trivial effect. I think we can summarise this by saying that renting students have a statistically significantly but utterly trivially higher score than the other cells on CORE total and CORE NR.

Do numeric predictors correlated with group affect how group impacts on dependent variables?

numPred	depVar	r.squared	adj.r.squared	p.valueFit	nobs	term	estimate	std.error	statistic	p.value
financial.dependency	EQ.index	0.025	0.022	5.5e-06	1076	groupstudents:predVal	0.016	0.0065	2.4	0.017
financial.dependency	WGO.positive	0.021	0.018	4.5e-05	1076	groupstudents:predVal	0.27	0.12	2.2	0.027
age	CORErisk	0.0092	0.0064	0.019	1076	groupstudents:predVal	0.021	0.01	2	0.042
sleeping.hours	COREtotal	0.05	0.047	6.7e-12	1076	groupstudents:predVal	-0.088	0.028	-3.1	0.0018
sleeping.hours	COREnr	0.054	0.052	6.8e-13	1076	groupstudents:predVal	-0.1	0.031	-3.3	0.0011

So again, though we tested out all four numeric predictors: *financial.dependency*, *age*, *sleeping.hours* and *exercising.week* for interactions with group in their relationship with all seven (*EQ.VAS*, *EQ.index*, *COREtotal*, *CORErisk*, *COREnr*, *WGO.positive* and *WGO.negative*), i.e. 28 possible interactions, only 5 showed

a statistically significant (at $p < .05$ this time) interaction with group in impact on any of the dependent variables and the specifics were that it was just three variable financial.dependency, age, sleeping.hours which had five statistically significant interactions with group in impacts on *EQ.index*, *WGO.positive*, *COREsrisk*, *COREtotal* and *COREnr*.

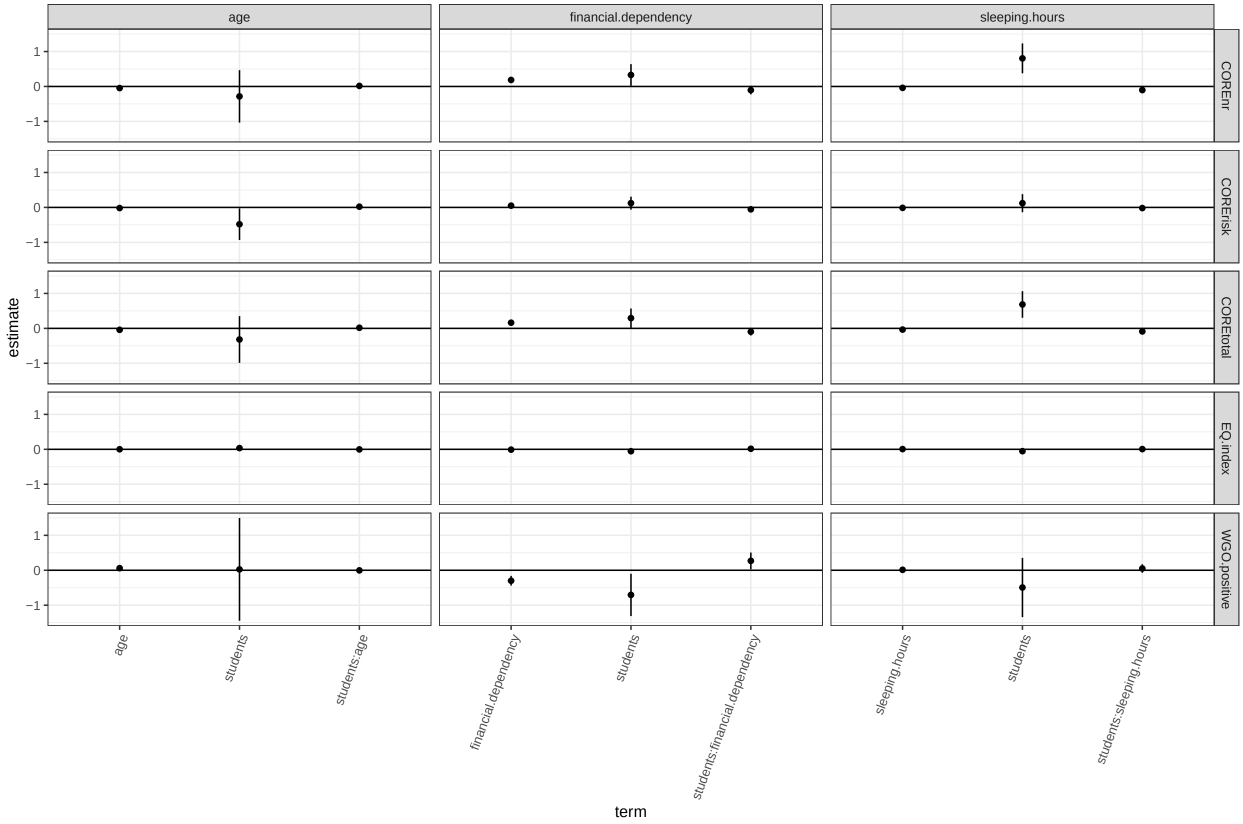

So this shows us we have three covariates *financial.dependency*, *age* and *sleeping.hours* with statistically significant impacts (at $p < .05$) on five: *EQ.index*, *WGO.positive*, *COREsrisk*, *COREtotal* and *COREnr*. The plot shows the impact of a unit increment in the covariate values on the dependent variables. I would say that inspecting those plots the summary is again that the impacts on the group differences are tiny if statistically significant.

(9) Distributions

It seems highly desirable to summarise the distributions of the measure scores in these two groups and combined to provide referential data for Quito.

We know that distributions are highly unlikely to be Gaussian. Let's look at the qqplots against a Gaussian model.

CORE-OM total score

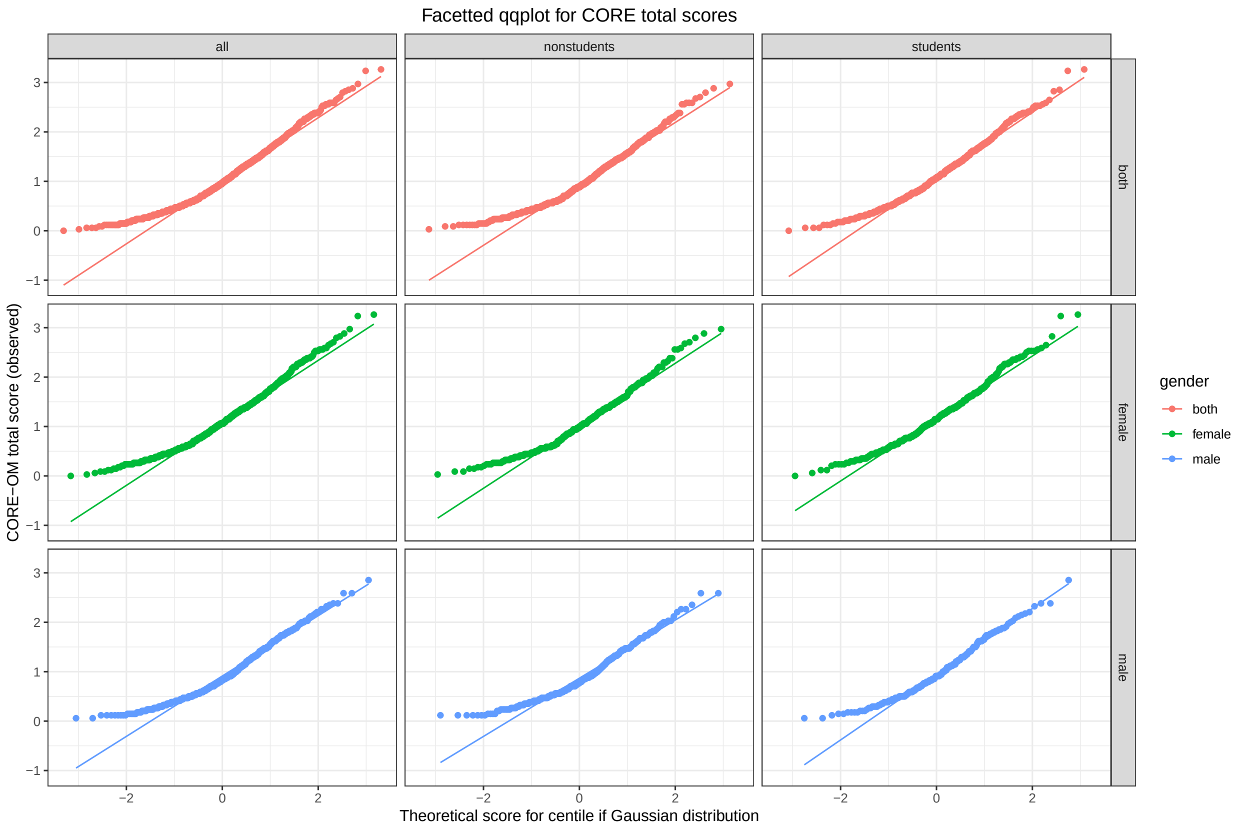

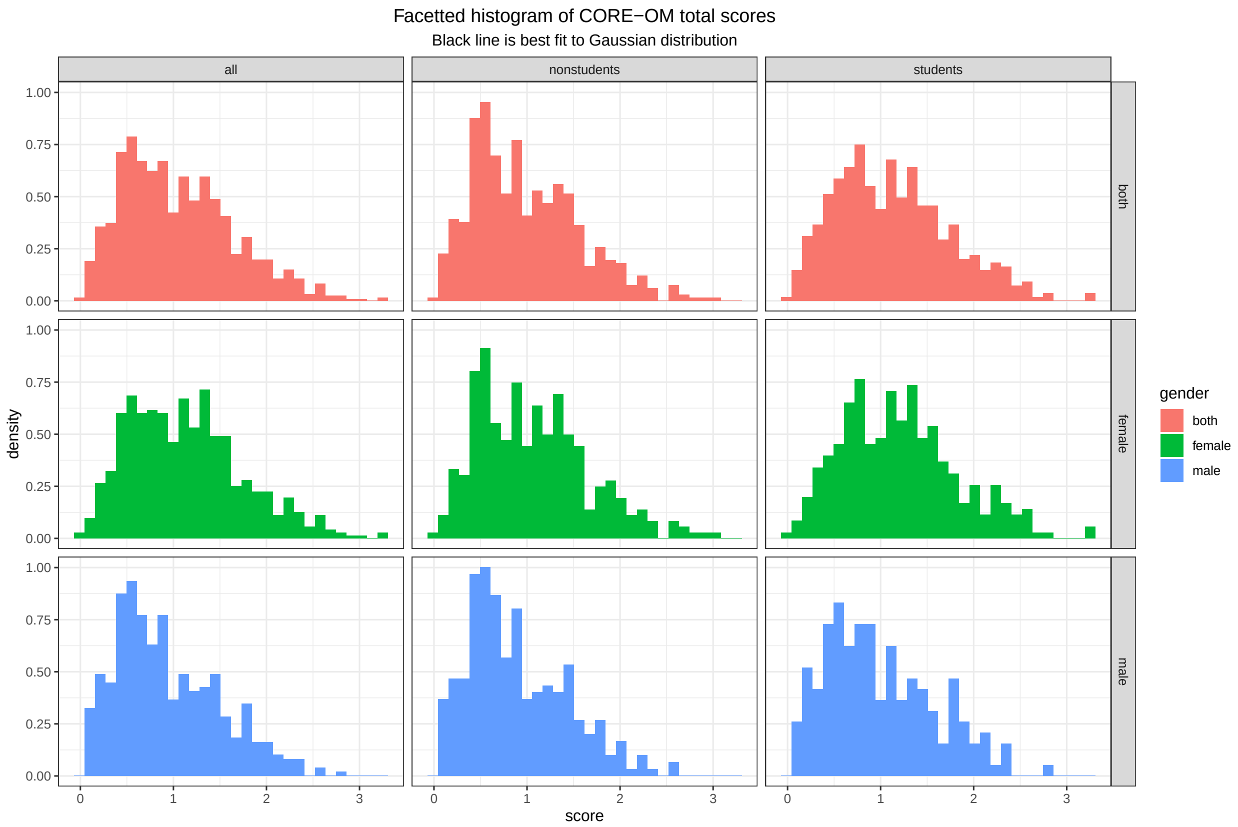

Clearly non-Gaussian with positive skew.

CORE-OM non-risk score

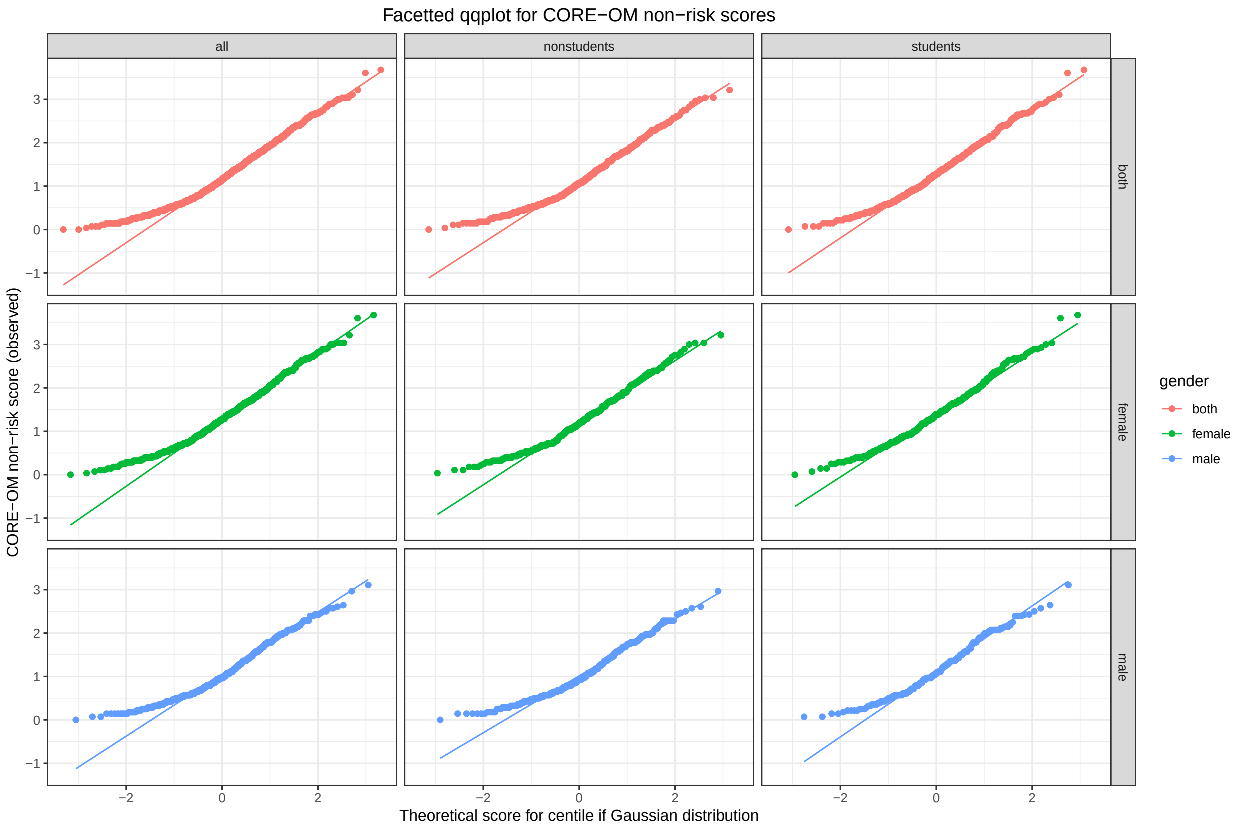

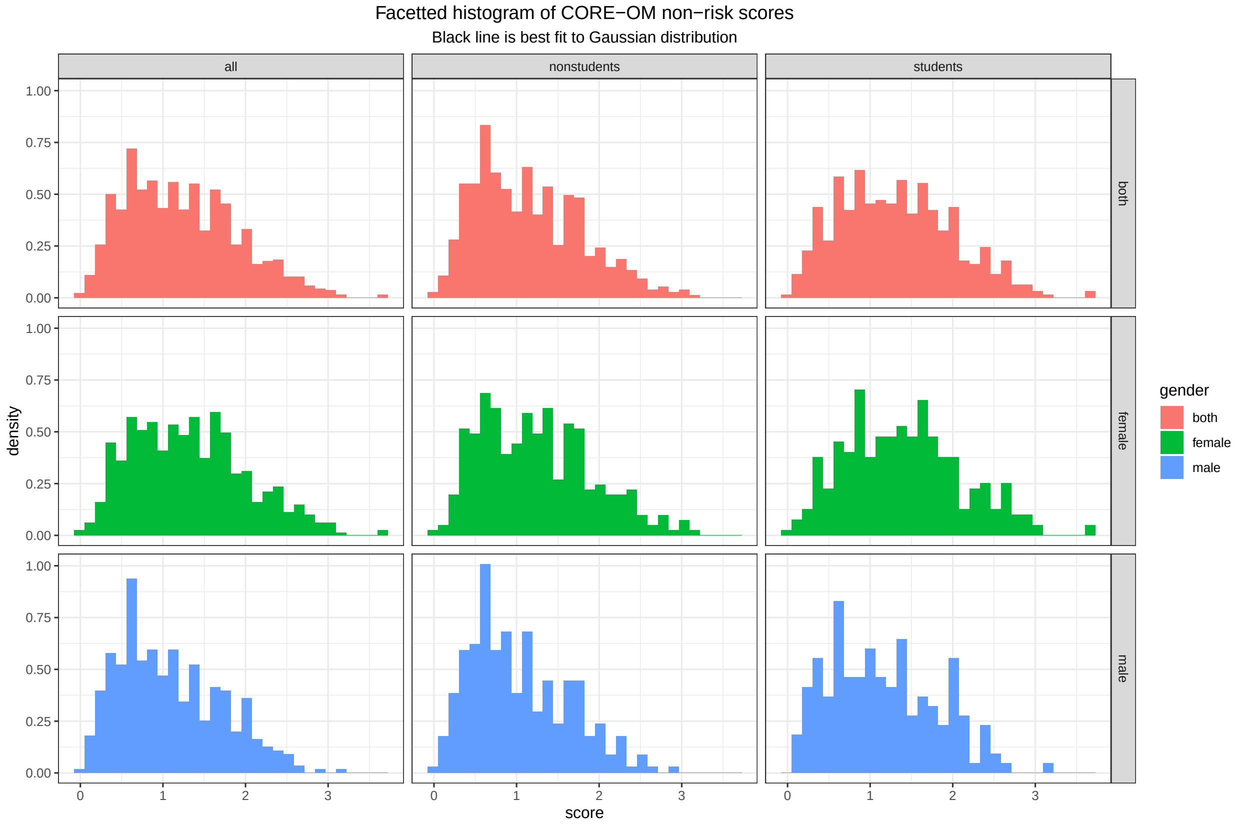

Similar: marked positive skew.

CORE-OM risk score

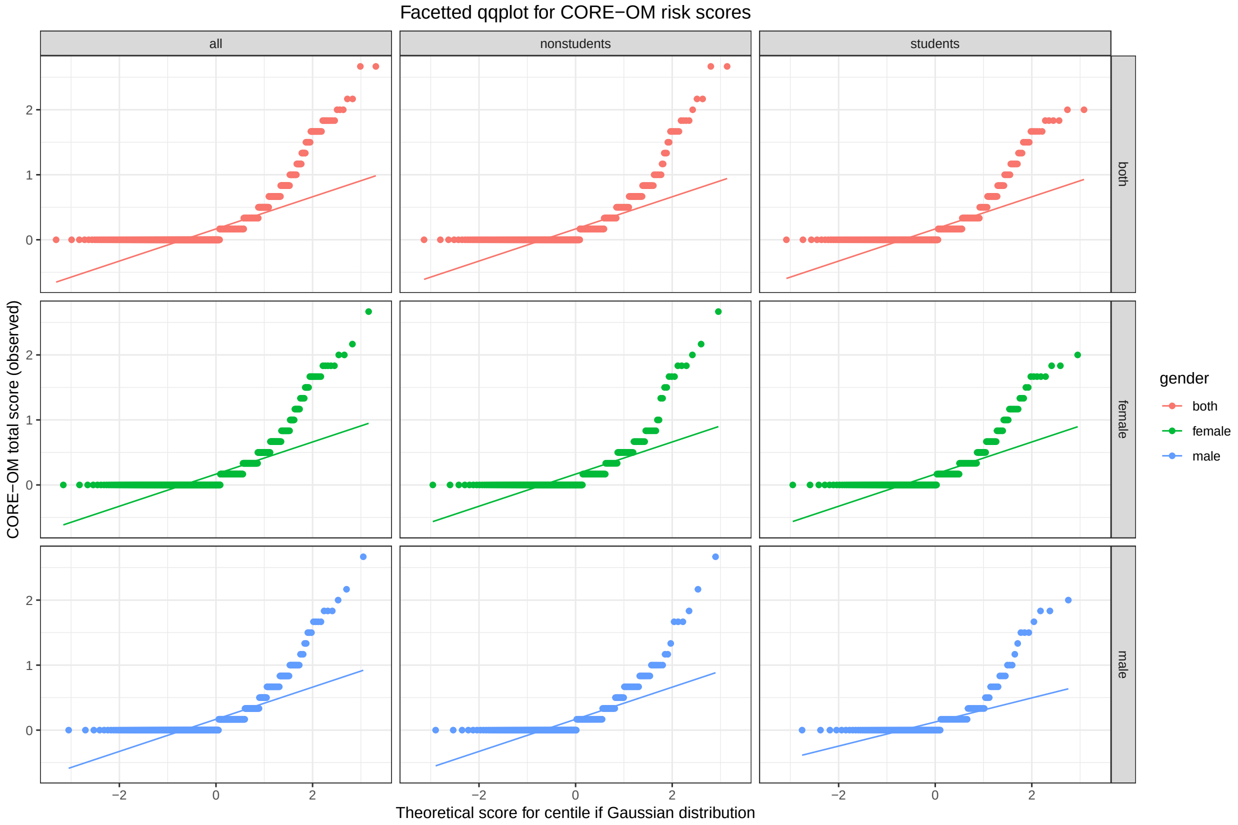

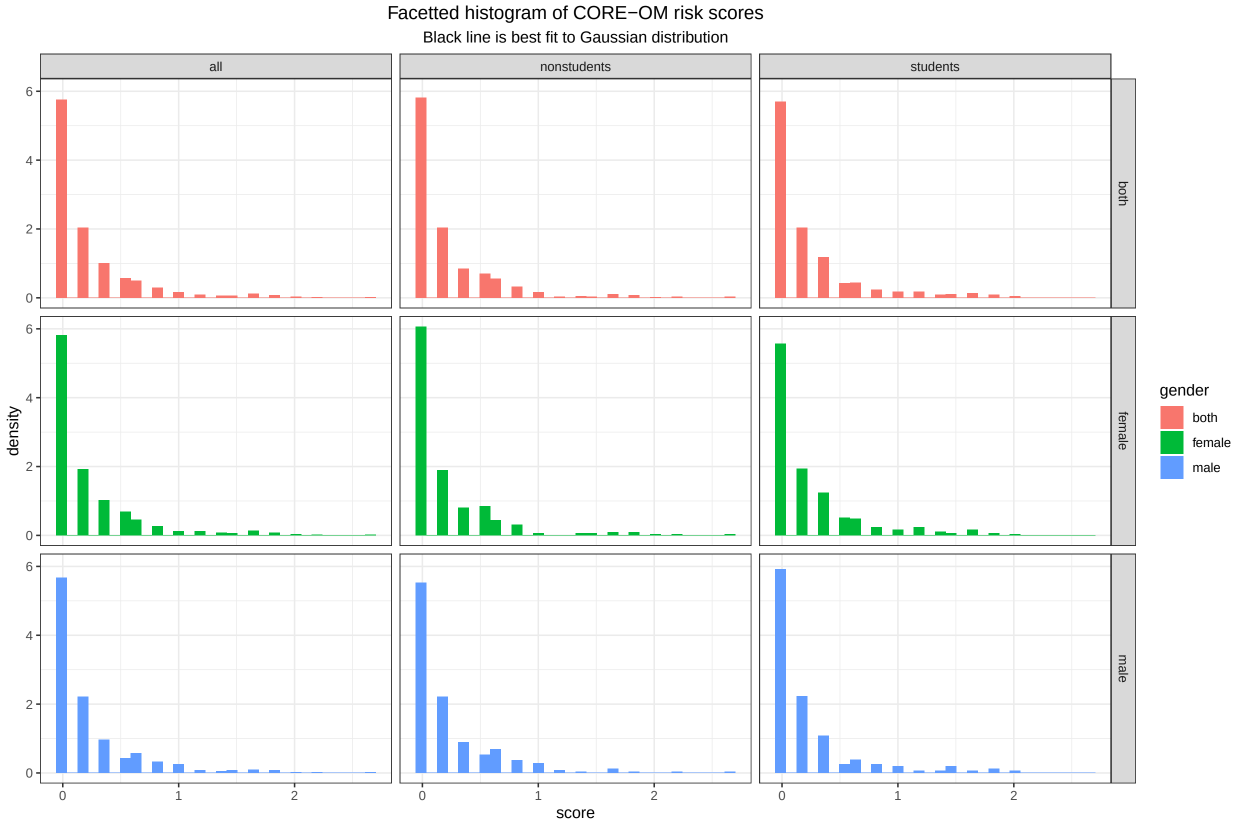

A particularly clearly non-Gaussian distribution with many zero scores.

EQ index score

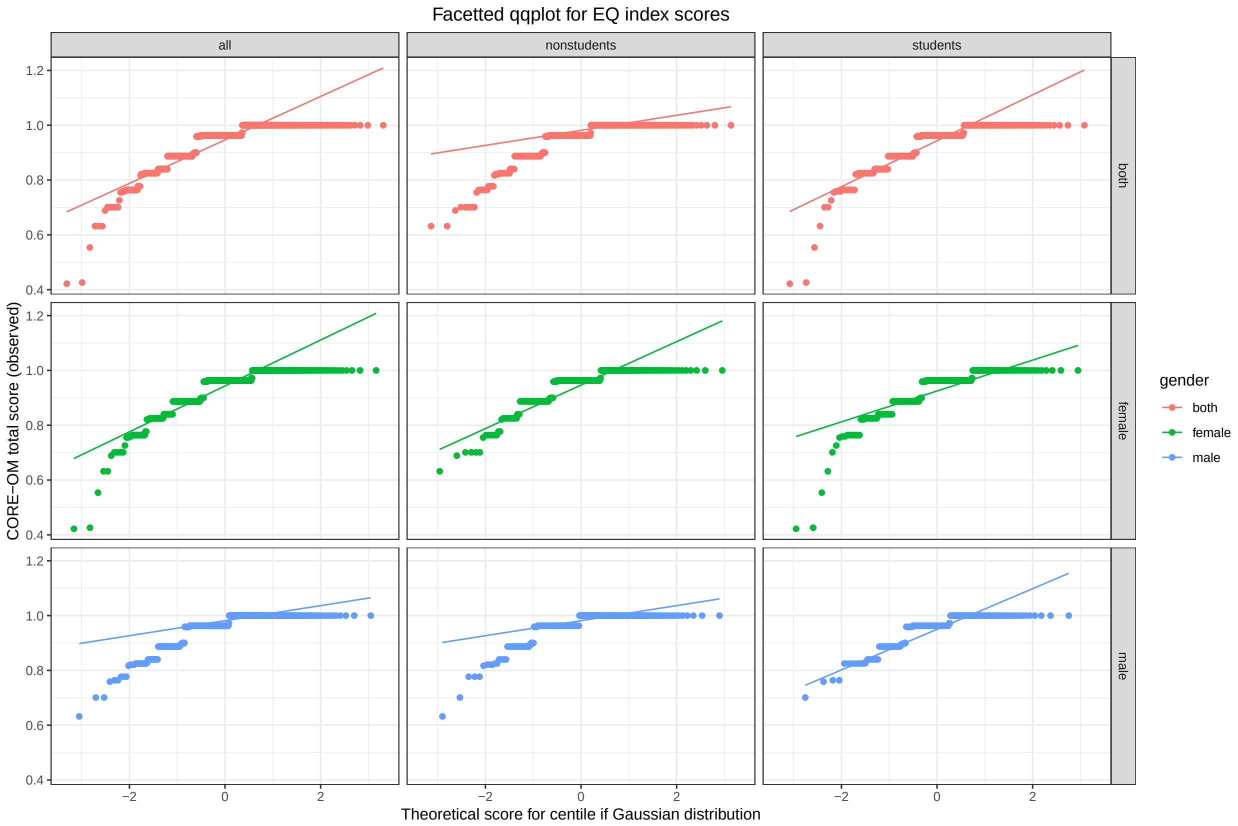

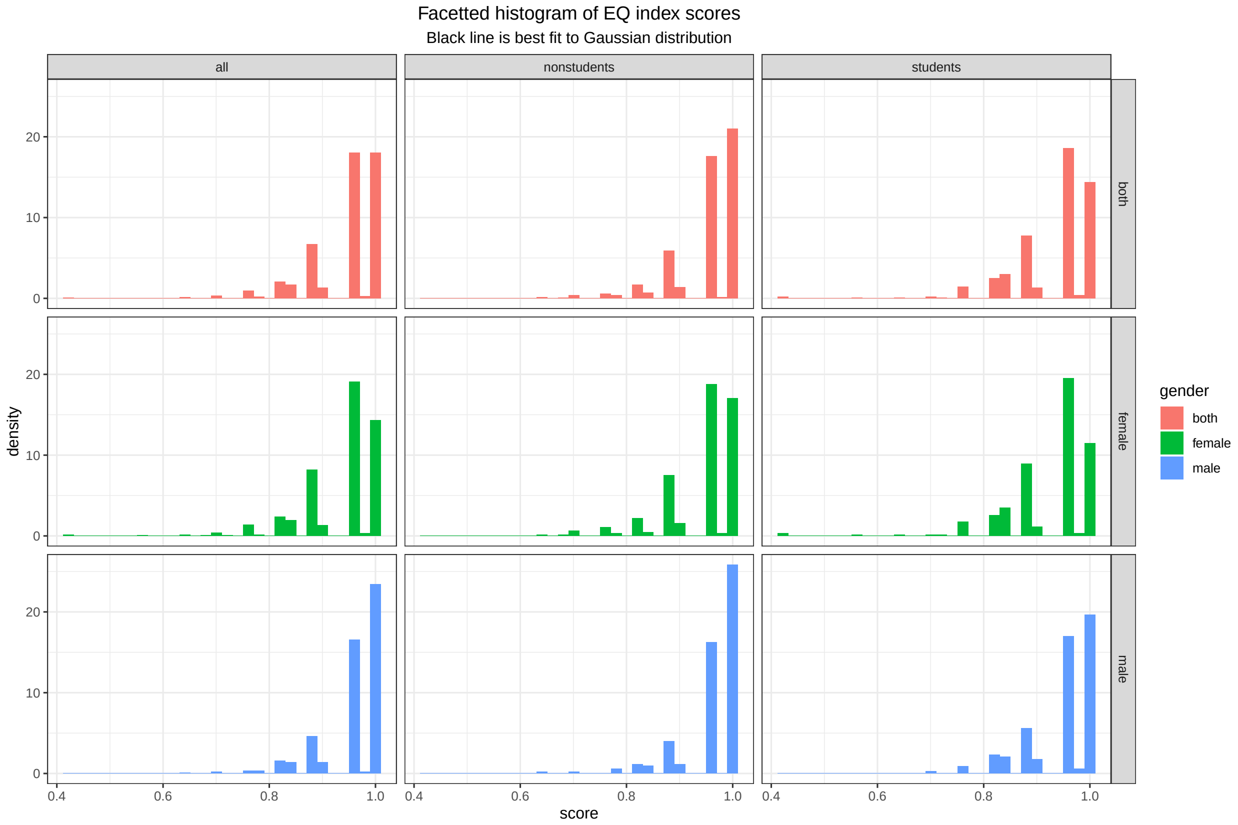

Inverse of the CORE risk: many high scorers (i.e. with good quality of life).

EQ VAS

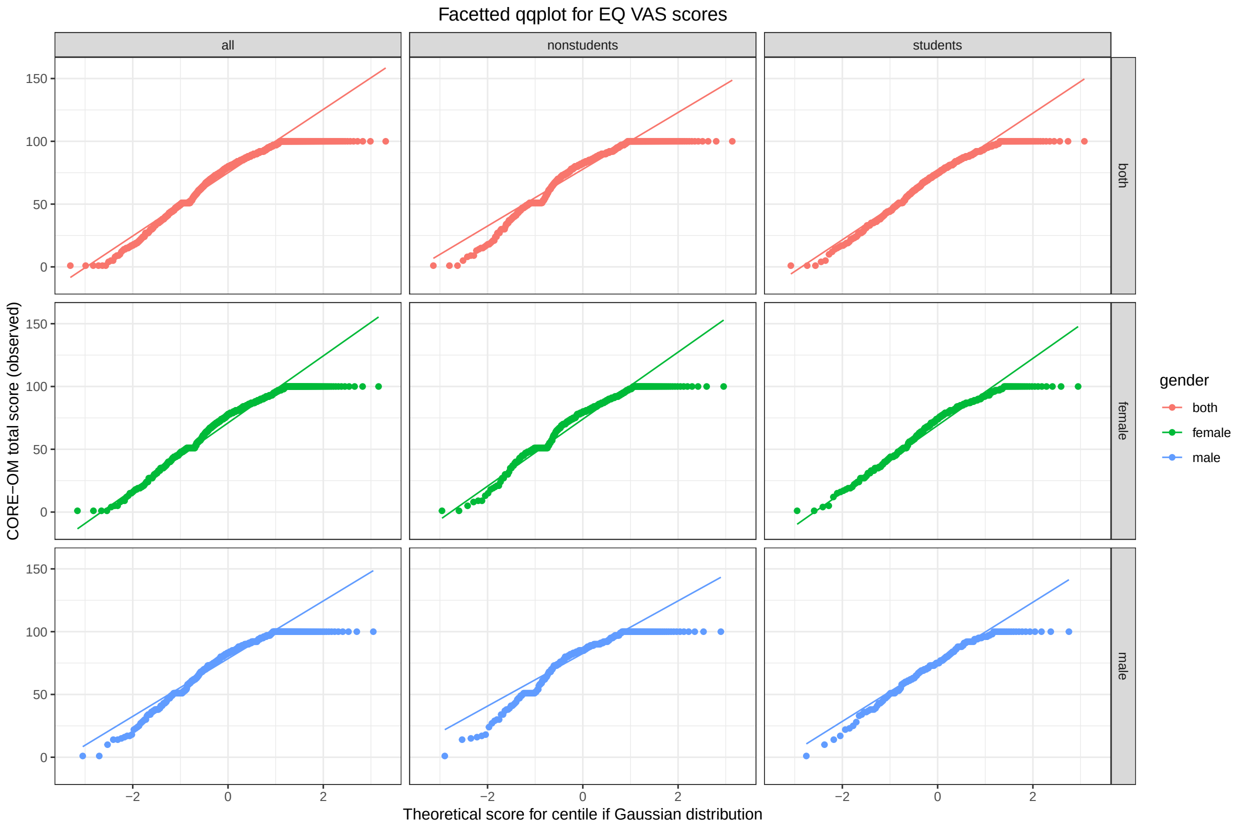
Facetted qqplot for EQ VAS scores
all
nonstudents
students
both
female
male
CORE−OM total score (observed)
Theoretical score for centile if Gaussian distribution
gender
both
female
male

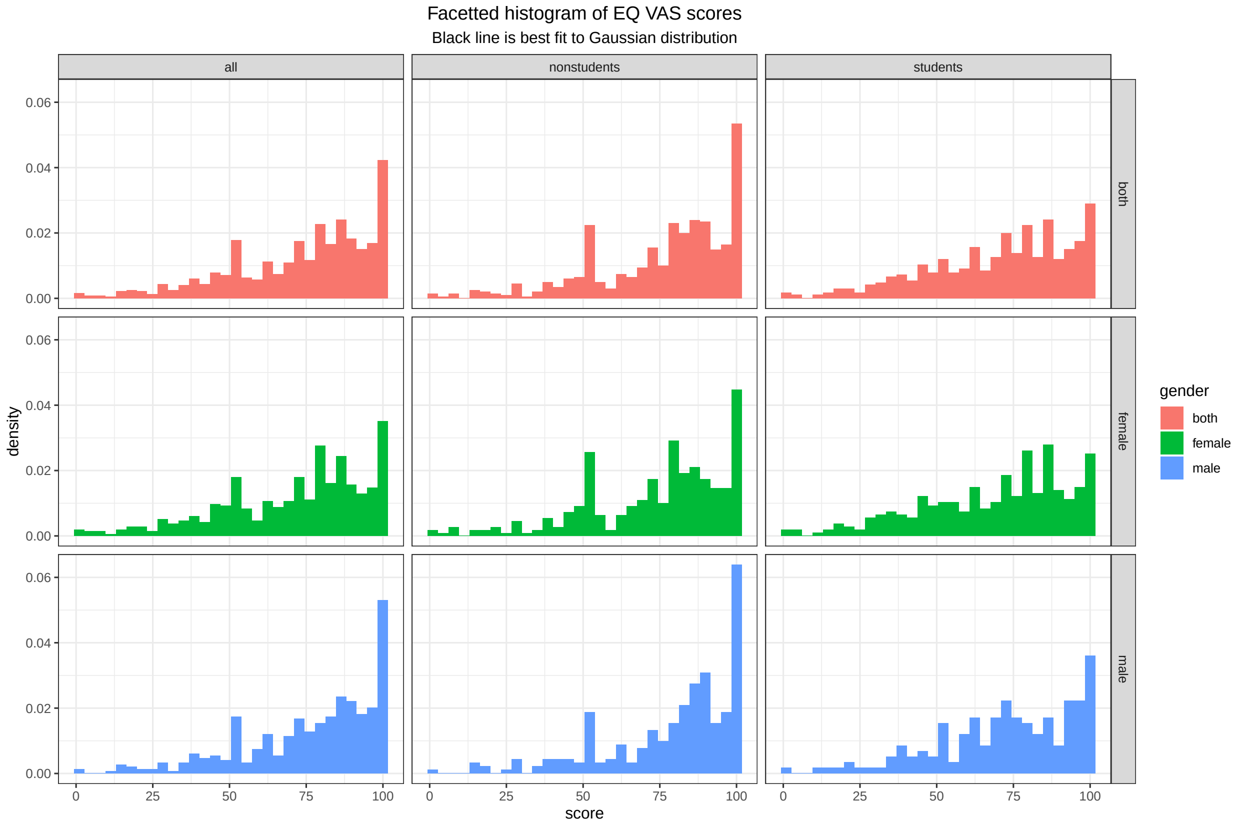
Facetted histogram of EQ VAS scores
Black line is best fit to Gaussian distribution
all
nonstudents
students
both
female
male
density
score
gender
both
female
male

Measured precisely by formr so much more continuous set of scores than for the EQ index, again, a good number of high QoL self-ratings.

WGO negative

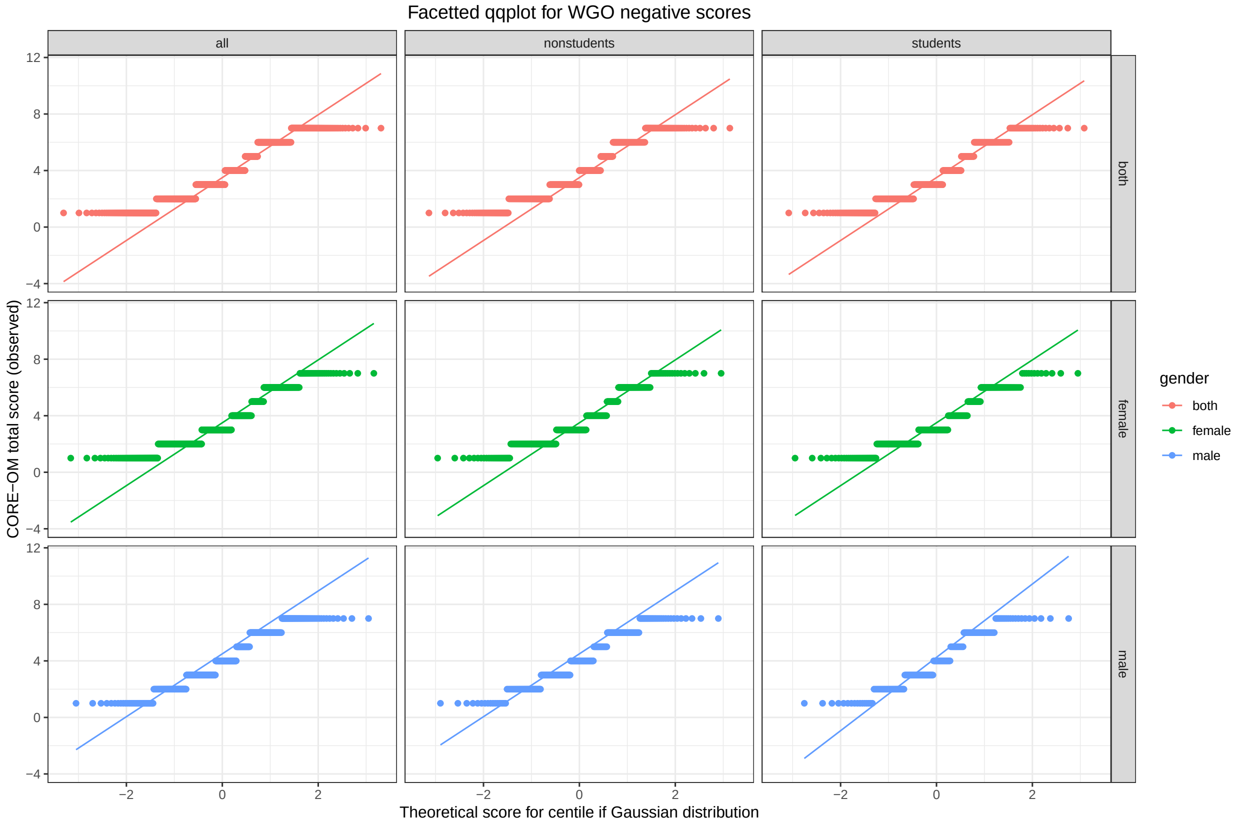

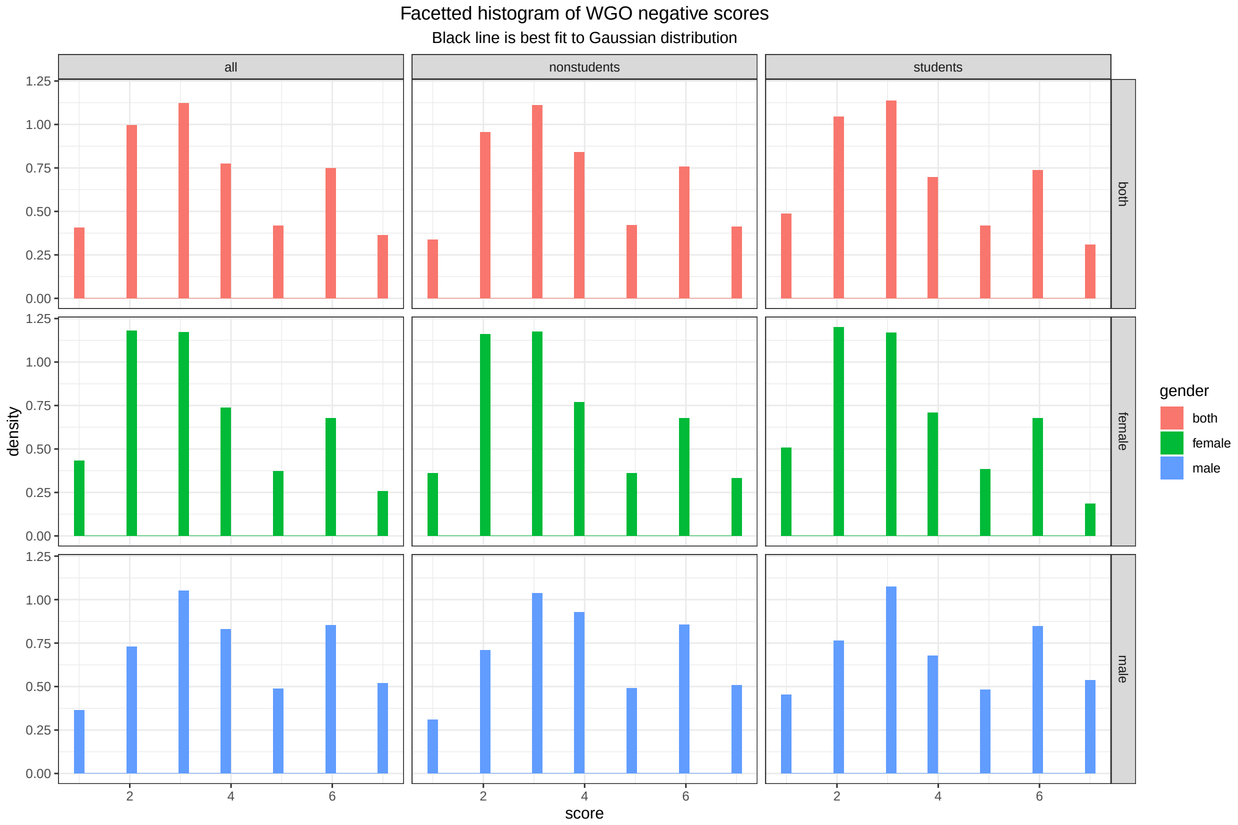

Very limited scaling hence highly discretised, limited step distribution.

This next is just experimenting with qqconf to get “Equal Local Levels” confidence intervals around the expected distribution for the entire sample, including both genders.

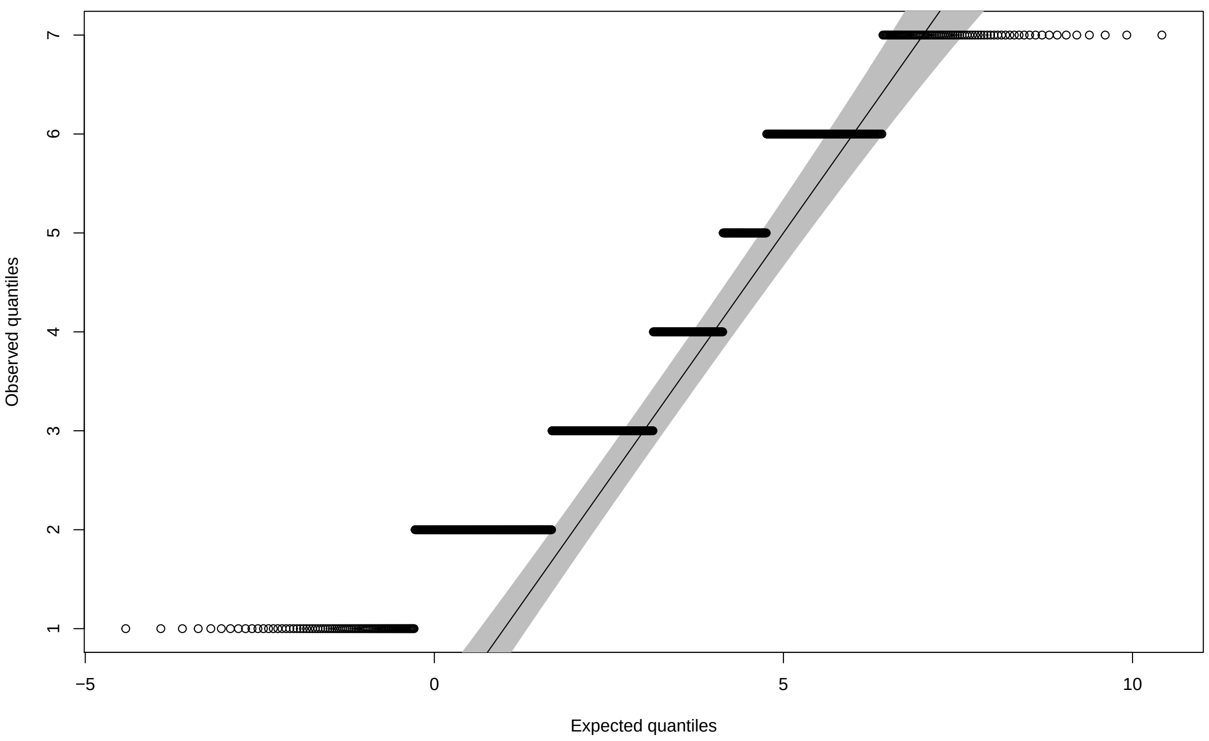

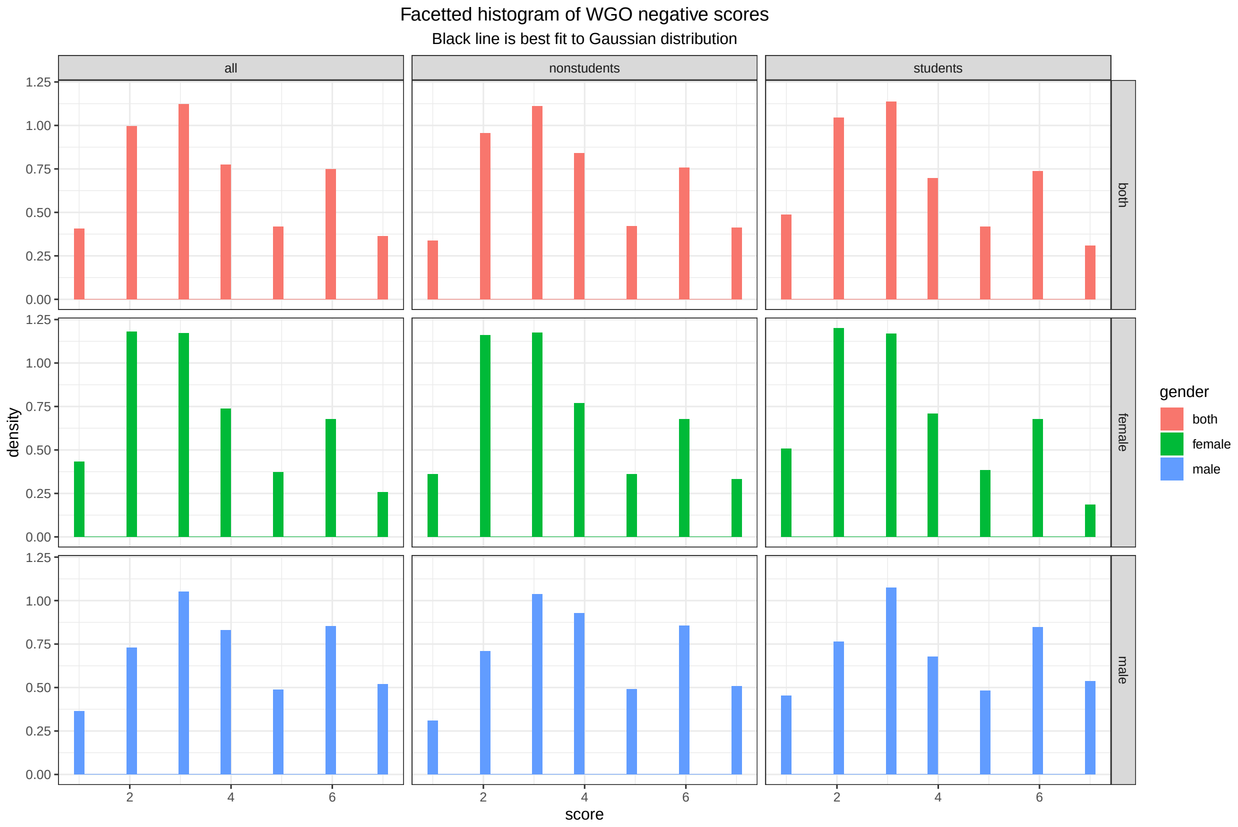

Clear that the distribution is heavy tailed, particularly for the low scores.

WGO positive

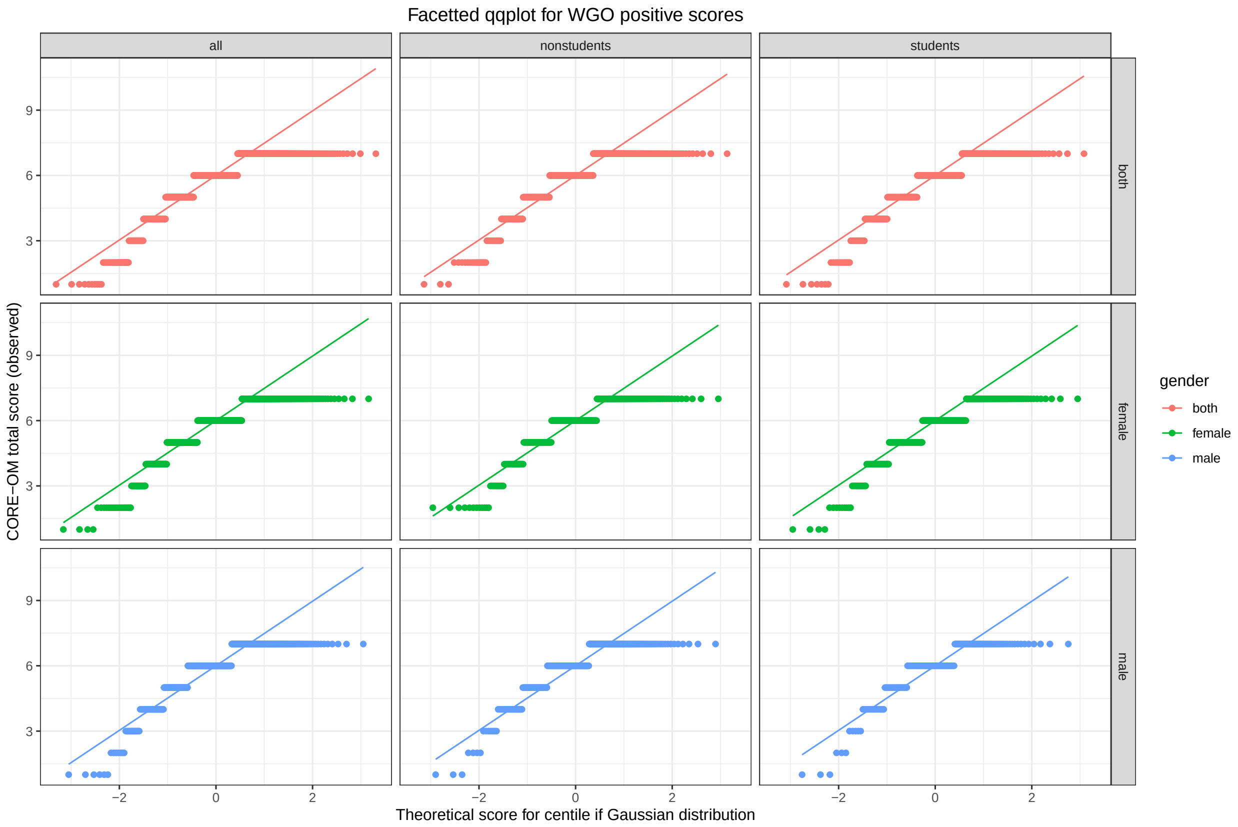

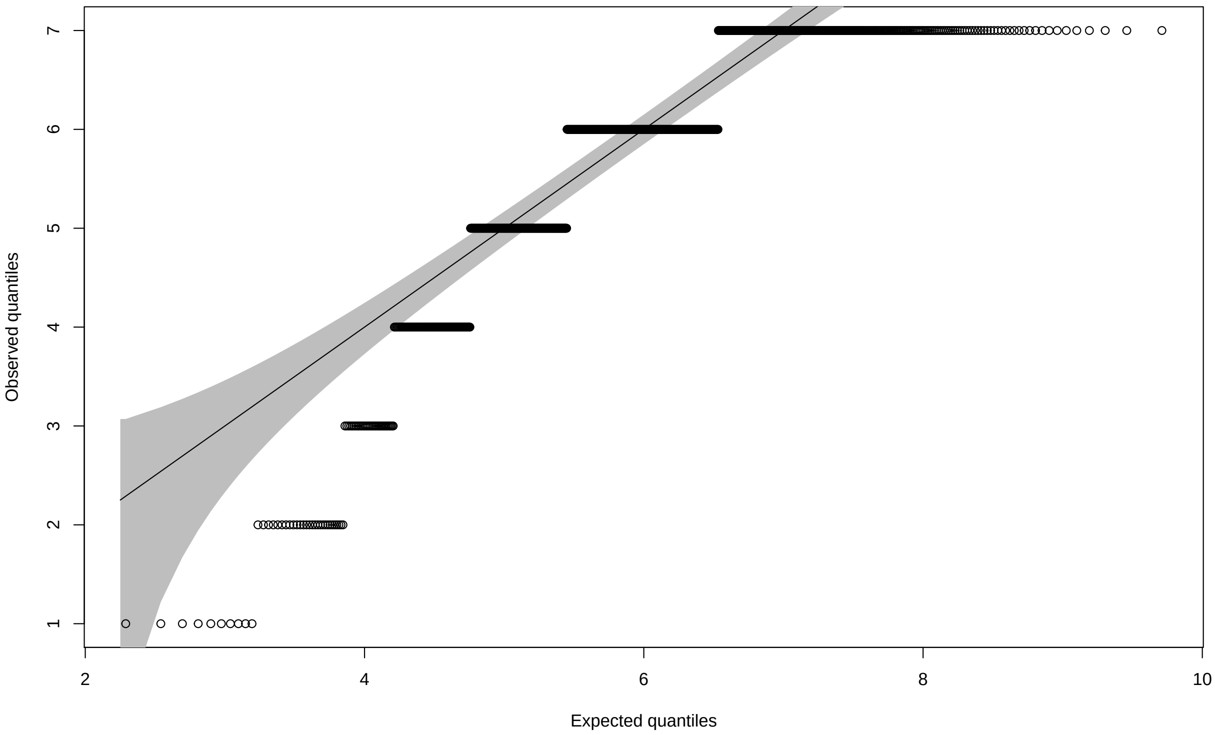

Interestingly different from the WGO negative but clearly not Gaussian.

Conventional summary statistics

We know from the exploration of distributions of the scores that they are extremely unlikely to have come from Gaussian population distributions so the mean and SD are imperfect summary statistics to understand the distribution. However, here are the conventional summary statistics by group and by gender, for pooled group by gender and for pooled gender by group and for the total sample.

measure	group	gender	minScore	obsmean	LCLmean	UCLmean	medianScore	LCLmedian	UCLmedian	maxScore
COREtotal	all	both	0.00	1.05	1.02	1.09	0.97	0.91	1.03	3.26
COREtotal	all	female	0.00	1.13	1.09	1.18	1.06	1.00	1.15	3.26
COREtotal	all	male	0.06	0.94	0.88	0.99	0.82	0.76	0.90	2.85
COREtotal	nonstudents	both	0.03	0.99	0.94	1.04	0.88	0.85	0.94	2.97
COREtotal	nonstudents	female	0.03	1.07	1.00	1.13	1.00	0.91	1.06	2.97
COREtotal	nonstudents	male	0.12	0.90	0.83	0.96	0.78	0.71	0.88	2.59
COREtotal	students	both	0.00	1.13	1.08	1.19	1.06	1.00	1.15	3.26
COREtotal	students	female	0.00	1.20	1.14	1.28	1.15	1.06	1.26	3.26
COREtotal	students	male	0.06	1.00	0.91	1.09	0.91	0.79	1.03	2.85
COREirisk	all	both	0.00	0.23	0.21	0.26	0.00	0.00	0.09	2.67
COREirisk	all	female	0.00	0.23	0.21	0.27	0.00	0.00	0.17	2.67
COREirisk	all	male	0.00	0.23	0.20	0.27	0.00	0.00	0.17	2.67
COREirisk	nonstudents	both	0.00	0.23	0.20	0.26	0.00	0.00	0.17	2.67
COREirisk	nonstudents	female	0.00	0.22	0.18	0.27	0.00	0.00	0.00	2.67
COREirisk	nonstudents	male	0.00	0.24	0.20	0.29	0.00	0.00	0.17	2.67
COREirisk	students	both	0.00	0.24	0.21	0.27	0.00	0.00	0.17	2.00

measure	group	gender	minScore	obsmean	LCLmean	UCLmean	medianScore	LCLmedian	UCLmedian	maxScore
COREsrisk	students	female	0.00	0.25	0.21	0.30	0.00	0.00	0.17	2.00
COREsrisk	students	male	0.00	0.22	0.16	0.29	0.00	0.00	0.17	2.00
COREnr	all	both	0.00	1.23	1.19	1.27	1.14	1.07	1.21	3.68
COREnr	all	female	0.00	1.33	1.28	1.38	1.29	1.21	1.36	3.68
COREnr	all	male	0.00	1.09	1.03	1.15	0.96	0.91	1.07	3.11
COREnr	nonstudents	both	0.00	1.15	1.10	1.20	1.07	0.97	1.11	3.21
COREnr	nonstudents	female	0.04	1.25	1.17	1.31	1.18	1.07	1.29	3.21
COREnr	nonstudents	male	0.00	1.04	0.96	1.11	0.93	0.82	1.02	2.96
COREnr	students	both	0.00	1.32	1.26	1.39	1.29	1.18	1.36	3.68
COREnr	students	female	0.00	1.41	1.33	1.49	1.39	1.25	1.46	3.68
COREnr	students	male	0.07	1.16	1.06	1.27	1.07	0.96	1.25	3.11
EQ.index	all	both	0.42	0.95	0.94	0.95	0.96	0.96	0.96	1.00
EQ.index	all	female	0.42	0.94	0.93	0.94	0.96	0.96	0.96	1.00
EQ.index	all	male	0.63	0.96	0.95	0.96	0.96	0.96	1.00	1.00
EQ.index	nonstudents	both	0.63	0.95	0.95	0.96	0.96	0.96	0.96	1.00
EQ.index	nonstudents	female	0.63	0.94	0.94	0.95	0.96	0.96	0.96	1.00
EQ.index	nonstudents	male	0.63	0.96	0.96	0.97	1.00	0.96	1.00	1.00
EQ.index	students	both	0.42	0.93	0.93	0.94	0.96	0.96	0.96	1.00
EQ.index	students	female	0.42	0.93	0.92	0.94	0.96	0.96	0.96	1.00
EQ.index	students	male	0.70	0.95	0.94	0.96	0.96	0.96	0.96	1.00
EQ.VAS	all	both	1.00	73.50	72.08	74.84	79.50	78.00	81.00	100.00
EQ.VAS	all	female	1.00	71.35	69.46	73.21	78.00	74.33	79.00	100.00
EQ.VAS	all	male	1.00	76.62	74.33	78.74	83.00	80.00	85.00	100.00
EQ.VAS	nonstudents	both	1.00	76.15	74.32	77.92	82.00	80.28	84.00	100.00
EQ.VAS	nonstudents	female	1.00	73.70	71.17	76.29	80.00	76.48	82.00	100.00
EQ.VAS	nonstudents	male	1.00	79.12	76.32	81.41	85.00	83.00	88.44	100.00
EQ.VAS	students	both	1.00	70.29	68.15	72.21	75.00	72.00	77.00	100.00
EQ.VAS	students	female	1.00	68.96	66.40	71.46	74.00	71.00	78.00	100.00
EQ.VAS	students	male	1.00	72.73	69.21	76.13	75.00	71.23	80.00	100.00
WGO.positive	all	both	1.00	5.75	5.66	5.82	6.00	6.00	6.00	7.00
WGO.positive	all	female	1.00	5.67	5.57	5.77	6.00	6.00	6.00	7.00
WGO.positive	all	male	1.00	5.85	5.73	5.97	6.00	6.00	6.00	7.00
WGO.positive	nonstudents	both	1.00	5.83	5.72	5.93	6.00	6.00	6.00	7.00
WGO.positive	nonstudents	female	2.00	5.78	5.63	5.91	6.00	6.00	6.00	7.00
WGO.positive	nonstudents	male	1.00	5.89	5.73	6.03	6.00	6.00	6.00	7.00
WGO.positive	students	both	1.00	5.65	5.53	5.77	6.00	6.00	6.00	7.00
WGO.positive	students	female	1.00	5.57	5.41	5.71	6.00	6.00	6.00	7.00
WGO.positive	students	male	1.00	5.80	5.59	5.99	6.00	6.00	6.00	7.00
WGO.negative	all	both	1.00	3.73	3.62	3.83	3.00	3.00	4.00	7.00
WGO.negative	all	female	1.00	3.52	3.37	3.66	3.00	3.00	3.00	7.00
WGO.negative	all	male	1.00	4.03	3.86	4.18	4.00	4.00	4.00	7.00
WGO.negative	nonstudents	both	1.00	3.82	3.68	3.96	4.00	3.00	4.00	7.00
WGO.negative	nonstudents	female	1.00	3.61	3.43	3.79	3.00	3.00	3.00	7.00
WGO.negative	nonstudents	male	1.00	4.07	3.86	4.27	4.00	4.00	4.00	7.00
WGO.negative	students	both	1.00	3.61	3.47	3.77	3.00	3.00	3.00	7.00
WGO.negative	students	female	1.00	3.42	3.23	3.61	3.00	3.00	3.00	7.00
WGO.negative	students	male	1.00	3.96	3.69	4.25	4.00	3.00	4.00	7.00

Centiles for scores

Given the non-Gaussian distributions, mean and SD values cannot be used to map a score to a probability (the "a score of x is over the 90th percentile, i.e. higher than that of 90% of emerging adults in Quito"). This means that empirical centiles should be used for that purpose. We report 5th, 10th, 20th, 30th, 40th, 50th, 60th, 70th, 80th, 90th and 95th centiles for the whole sample and by group and gender. Centiles are reported with 95% confidence intervals computed using the quantileCI R package which implements the Nyblom 1991 method to create the CI.

Centiles for CORE-OM total score

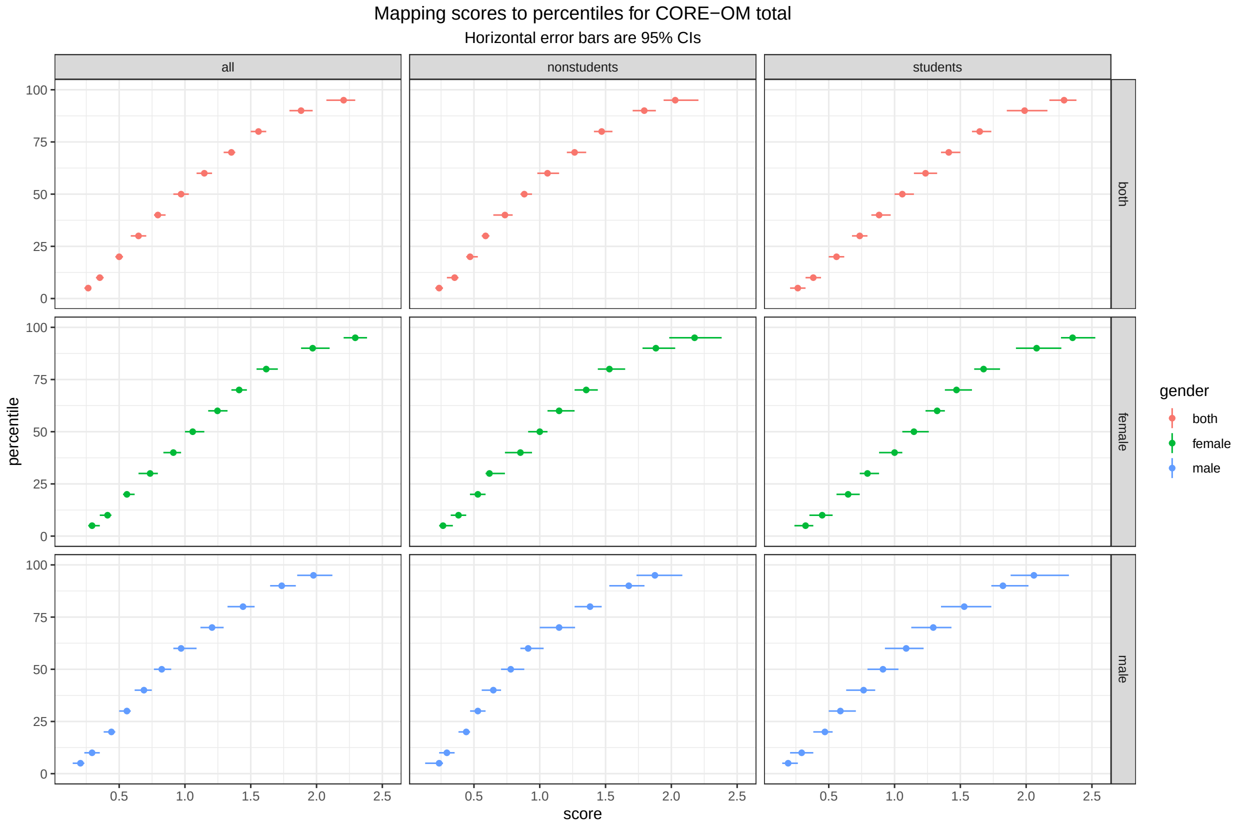

Clearly some gender and probably group differences in the locations. OK, look at gender.

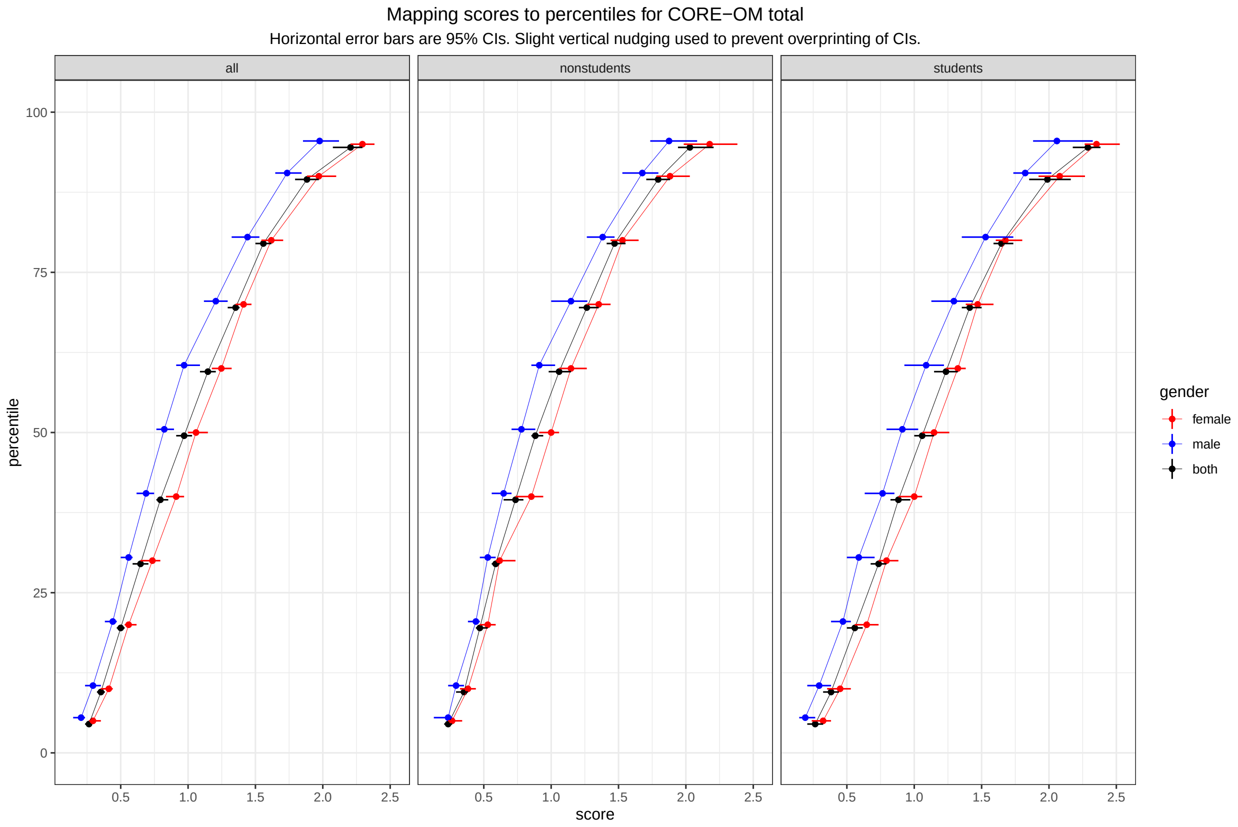

So clear differences by gender in the total sample and within groups and the confidence intervals of the scores for most of the centiles show gender differences are highly unlikely to have arisen by sampling vagaries.

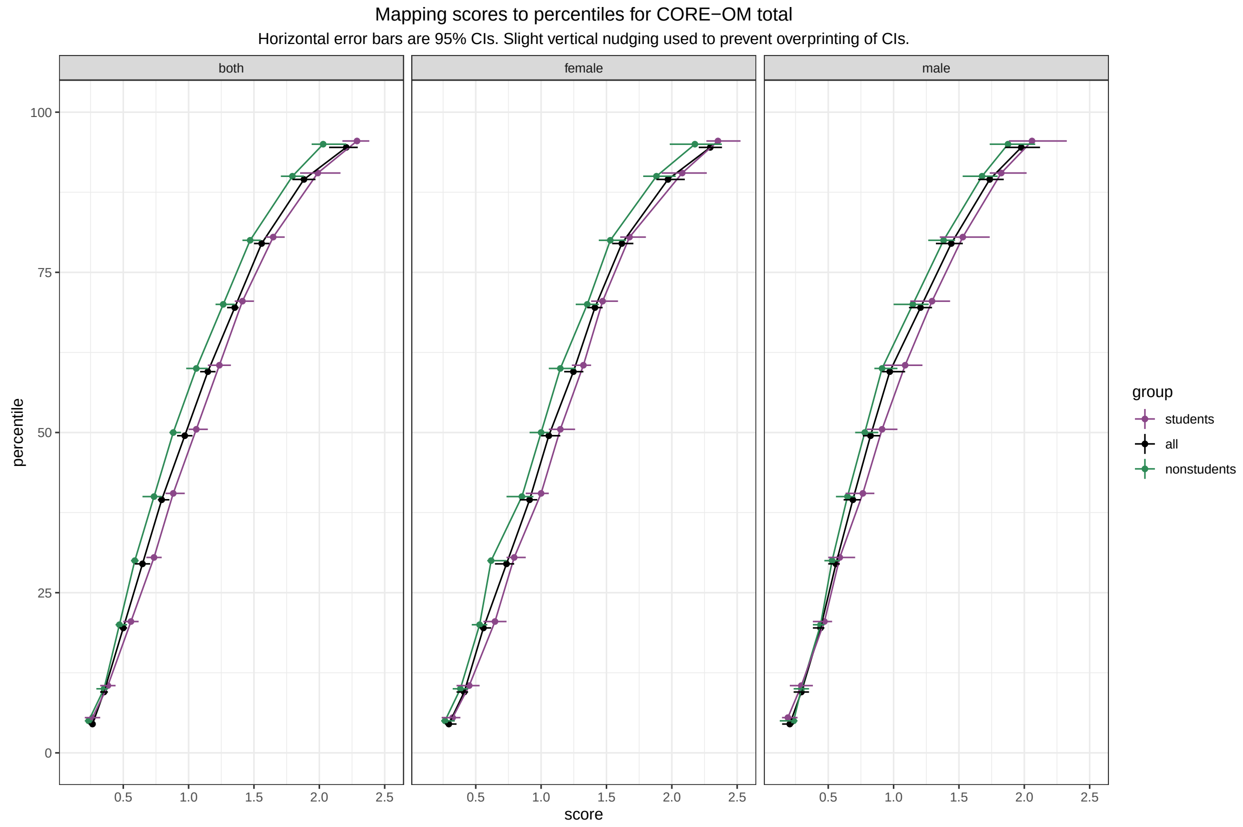

There are systematic differences with the students generally having the same or slightly higher scores than the non-students for the same centiles but for many of the centiles the differences are within, or not much different from, the CIs of the centile estimates.

Here is the table for these CORE-OM total centiles.

group	gender	5th	10th	20th	30th	40th	50th	60th	70th	80th	90th	95th
all	both	0.26[0.24, 0.28]	0.35[0.32, 0.38]	0.50[0.47, 0.53]	0.65[0.59, 0.71]	0.79[0.76, 0.85]	0.97[0.91, 1.03]	1.15[1.09, 1.21]	1.35[1.29, 1.38]	1.56[1.50, 1.62]	1.88[1.79, 1.97]	2.21[2.07, 2.29]
all	female	0.29[0.26, 0.35]	0.41[0.35, 0.44]	0.56[0.53, 0.62]	0.74[0.65, 0.79]	0.91[0.84, 0.97]	1.06[1.00, 1.15]	1.25[1.18, 1.32]	1.41[1.35, 1.47]	1.62[1.54, 1.71]	1.97[1.88, 2.10]	2.29[2.21, 2.38]
all	male	0.21[0.15, 0.24]	0.29[0.24, 0.35]	0.44[0.38, 0.47]	0.56[0.50, 0.59]	0.69[0.62, 0.75]	0.82[0.76, 0.90]	0.97[0.91, 1.09]	1.21[1.12, 1.29]	1.44[1.32, 1.53]	1.74[1.65, 1.84]	1.98[1.85, 2.12]
nonstudents	both	0.24[0.21, 0.26]	0.35[0.29, 0.38]	0.47[0.44, 0.53]	0.59[0.56, 0.62]	0.74[0.65, 0.79]	0.88[0.85, 0.94]	1.06[0.98, 1.15]	1.26[1.21, 1.35]	1.47[1.41, 1.55]	1.79[1.71, 1.88]	2.03[1.94, 2.21]
nonstudents	female	0.26[0.24, 0.34]	0.38[0.32, 0.44]	0.53[0.47, 0.59]	0.62[0.59, 0.74]	0.85[0.74, 0.94]	1.00[0.91, 1.06]	1.15[1.06, 1.26]	1.35[1.26, 1.44]	1.53[1.44, 1.65]	1.88[1.78, 2.03]	2.18[1.98, 2.38]
nonstudents	male	0.24[0.13, 0.26]	0.29[0.24, 0.35]	0.44[0.38, 0.47]	0.53[0.47, 0.59]	0.65[0.56, 0.71]	0.78[0.71, 0.88]	0.91[0.85, 1.03]	1.15[1.00, 1.27]	1.38[1.26, 1.47]	1.68[1.53, 1.80]	1.88[1.74, 2.08]
students	both	0.26[0.21, 0.32]	0.38[0.32, 0.44]	0.56[0.50, 0.62]	0.74[0.68, 0.79]	0.88[0.82, 0.97]	1.06[1.00, 1.15]	1.24[1.15, 1.32]	1.41[1.35, 1.50]	1.65[1.59, 1.74]	1.99[1.85, 2.16]	2.29[2.18, 2.38]
students	female	0.32[0.24, 0.38]	0.45[0.35, 0.53]	0.65[0.56, 0.74]	0.79[0.74, 0.88]	1.00[0.88, 1.06]	1.15[1.06, 1.26]	1.32[1.24, 1.38]	1.47[1.38, 1.59]	1.68[1.61, 1.80]	2.08[1.92, 2.27]	2.35[2.26, 2.53]
students	male	0.19[0.15, 0.27]	0.29[0.21, 0.38]	0.47[0.38, 0.53]	0.59[0.50, 0.71]	0.76[0.63, 0.85]	0.91[0.79, 1.03]	1.09[0.93, 1.22]	1.29[1.13, 1.43]	1.53[1.35, 1.74]	1.82[1.74, 2.02]	2.06[1.88, 2.33]

Centiles for CORE-OM non-risk score

This is bound to be very similar to the total score as all 28 non-risk items are in the 34 items in the full measure.

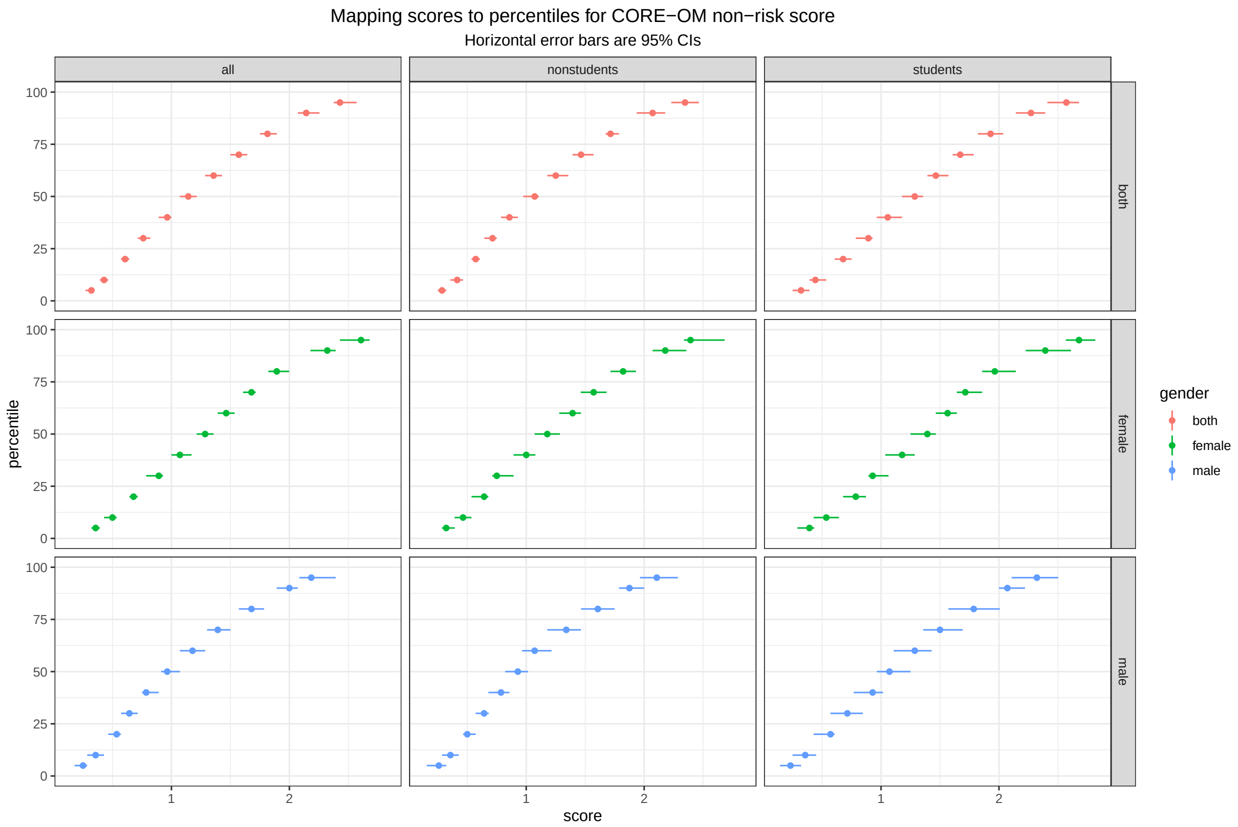

Indeed, very similar to the total score!

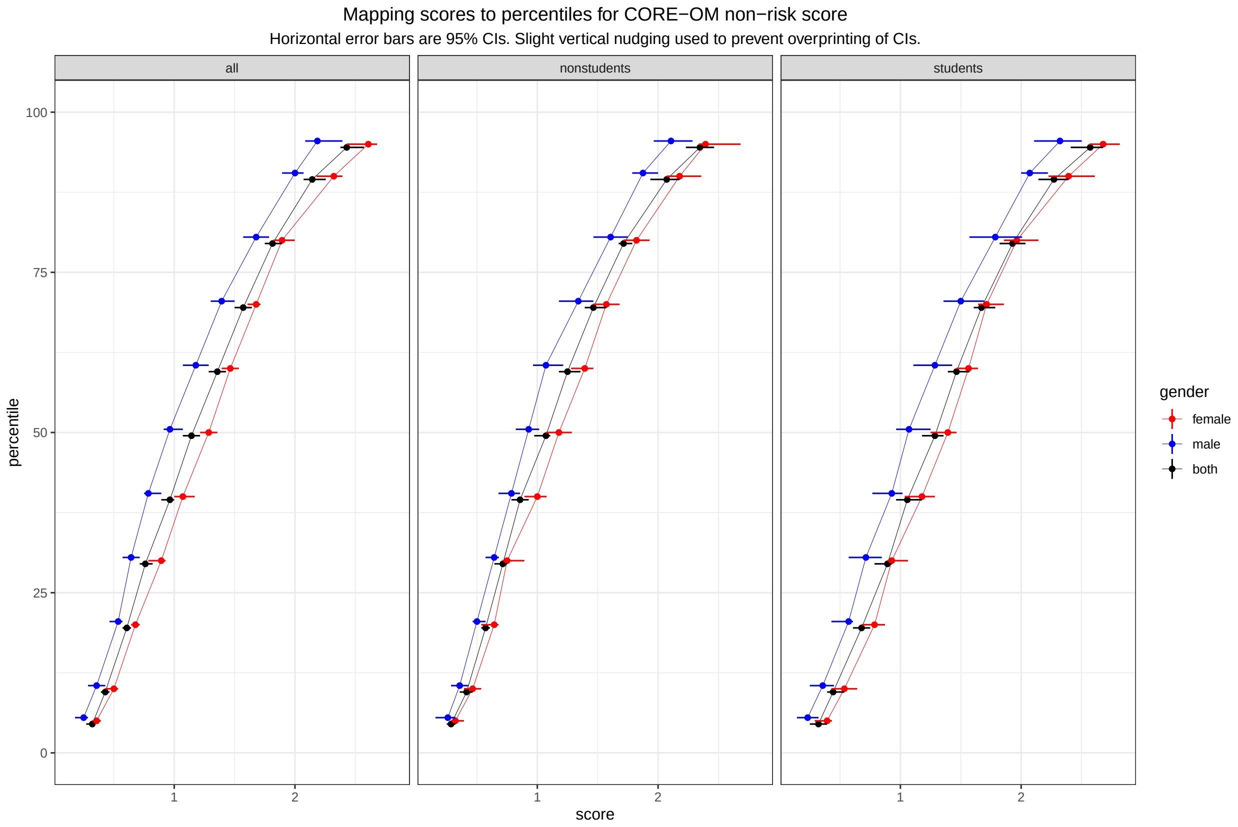

Again clear differences by gender in the total sample and within groups and the confidence intervals of the scores for most of the centiles show gender differences are highly unlikely to have arisen by sampling vagaries (except for 5th centile in the non-student group).

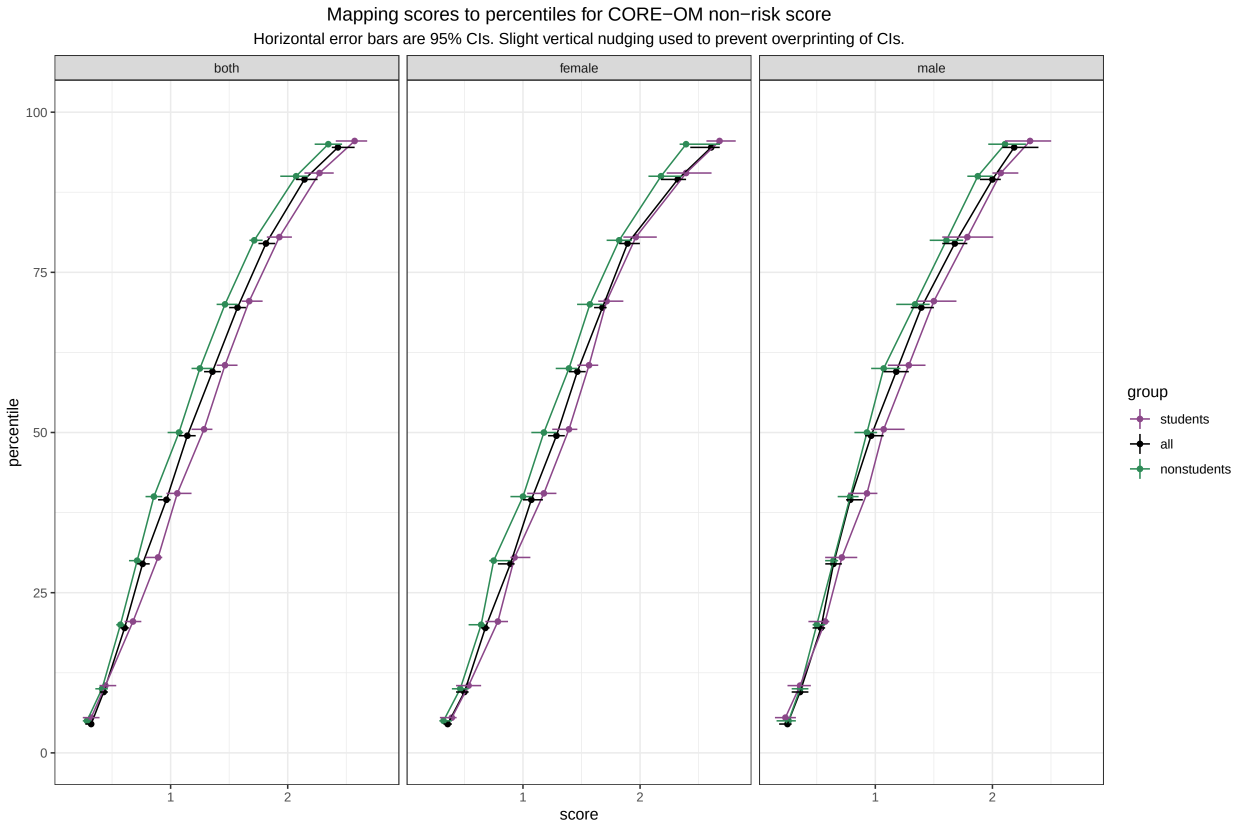

Again, essentially the same as for the CORE-OM total score: systematic differences with the students generally having the same or slightly higher scores than the non-students for the same centiles but for many of the centiles the differences are within, or not much different from, the CIs of the centile estimates.

Here is the table for these CORE-OM non-risk centiles.

group	gender	5th	10th	20th	30th	40th	50th	60th	70th	80th	90th	95th
all	both	0.32[0.27, 0.32]	0.43[0.39, 0.46]	0.61[0.57, 0.64]	0.76[0.71, 0.82]	0.96[0.89, 1.00]	1.14[1.07, 1.21]	1.36[1.29, 1.43]	1.57[1.50, 1.64]	1.81[1.75, 1.89]	2.14[2.07, 2.26]	2.43[2.38, 2.57]
all	female	0.36[0.32, 0.39]	0.50[0.43, 0.54]	0.68[0.64, 0.71]	0.89[0.79, 0.93]	1.07[1.00, 1.17]	1.29[1.21, 1.36]	1.46[1.39, 1.54]	1.68[1.61, 1.71]	1.89[1.82, 2.00]	2.32[2.18, 2.39]	2.61[2.43, 2.68]
all	male	0.25[0.18, 0.29]	0.36[0.29, 0.43]	0.54[0.46, 0.57]	0.64[0.57, 0.71]	0.79[0.75, 0.89]	0.96[0.91, 1.07]	1.18[1.07, 1.29]	1.39[1.30, 1.50]	1.68[1.57, 1.79]	2.00[1.89, 2.07]	2.19[2.08, 2.39]
nonstudents	both	0.29[0.25, 0.32]	0.41[0.36, 0.46]	0.57[0.54, 0.61]	0.71[0.64, 0.75]	0.86[0.79, 0.93]	1.07[0.97, 1.11]	1.25[1.18, 1.36]	1.46[1.39, 1.57]	1.71[1.67, 1.79]	2.07[1.94, 2.18]	2.35[2.23, 2.46]
nonstudents	female	0.32[0.29, 0.39]	0.46[0.39, 0.54]	0.64[0.54, 0.68]	0.75[0.71, 0.89]	1.00[0.89, 1.08]	1.18[1.07, 1.29]	1.39[1.28, 1.46]	1.57[1.46, 1.68]	1.82[1.71, 1.93]	2.18[2.07, 2.36]	2.39[2.34, 2.68]
nonstudents	male	0.26[0.16, 0.32]	0.36[0.29, 0.43]	0.50[0.46, 0.57]	0.64[0.57, 0.68]	0.79[0.68, 0.86]	0.93[0.82, 1.02]	1.07[0.96, 1.21]	1.34[1.18, 1.46]	1.61[1.46, 1.75]	1.88[1.79, 2.00]	2.11[1.96, 2.29]
students	both	0.32[0.25, 0.39]	0.44[0.39, 0.54]	0.68[0.61, 0.75]	0.89[0.79, 0.93]	1.06[0.96, 1.18]	1.29[1.18, 1.36]	1.46[1.39, 1.57]	1.67[1.61, 1.79]	1.93[1.82, 2.04]	2.27[2.14, 2.39]	2.57[2.41, 2.68]
students	female	0.39[0.29, 0.43]	0.54[0.43, 0.64]	0.79[0.68, 0.87]	0.93[0.89, 1.06]	1.18[1.04, 1.29]	1.39[1.25, 1.46]	1.56[1.46, 1.64]	1.71[1.64, 1.86]	1.96[1.86, 2.14]	2.39[2.23, 2.61]	2.68[2.57, 2.82]
students	male	0.23[0.14, 0.32]	0.36[0.25, 0.45]	0.57[0.43, 0.61]	0.71[0.57, 0.85]	0.93[0.77, 1.02]	1.07[0.96, 1.25]	1.29[1.11, 1.43]	1.50[1.36, 1.69]	1.79[1.57, 2.01]	2.07[2.00, 2.22]	2.32[2.11, 2.50]

Centiles for CORE-OM risk score

Should be very different from the CORE-OM total and non-risk scores given the reduced number of possible (and observed) scores and the large proportions of zero scorers

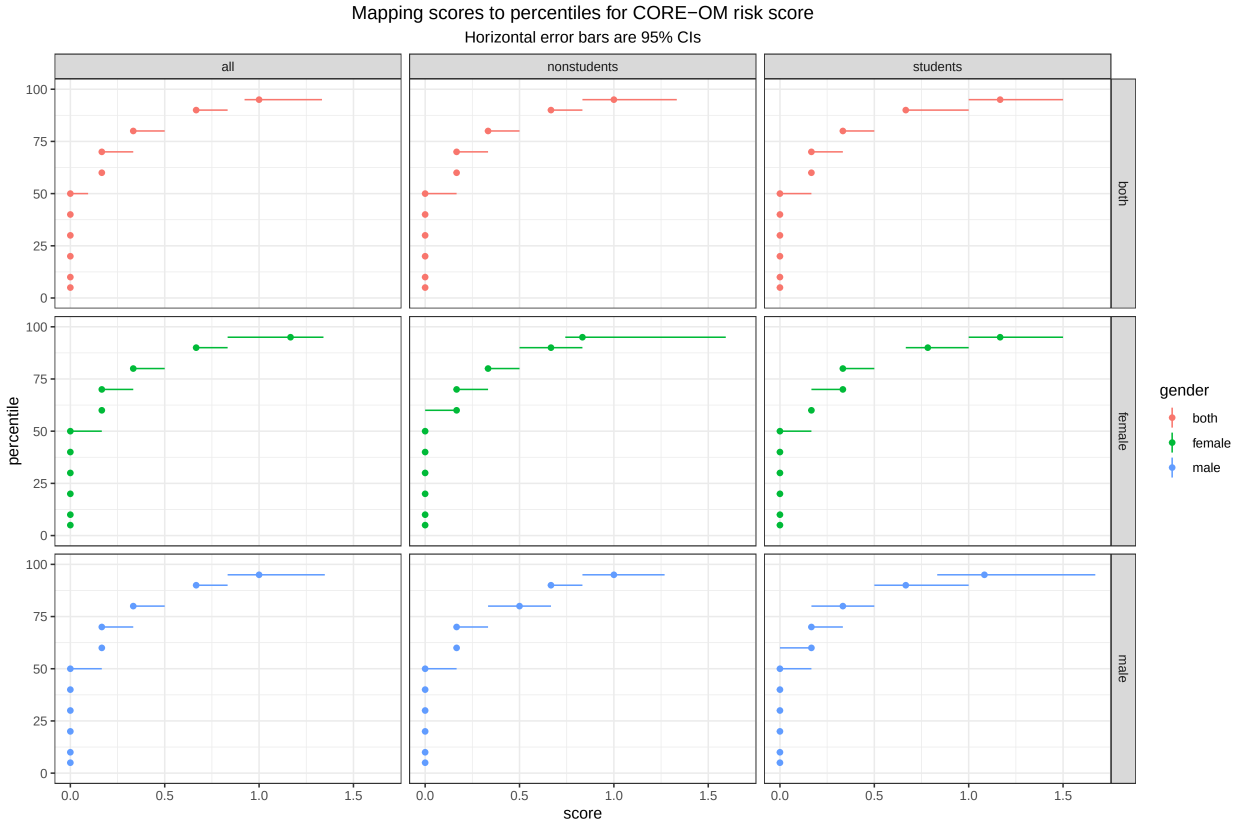

Indeed, radically different with about half the sample scoring zero (actually 52.8%) the centiles up to the median/50th are all zero in all group/gender combinations.

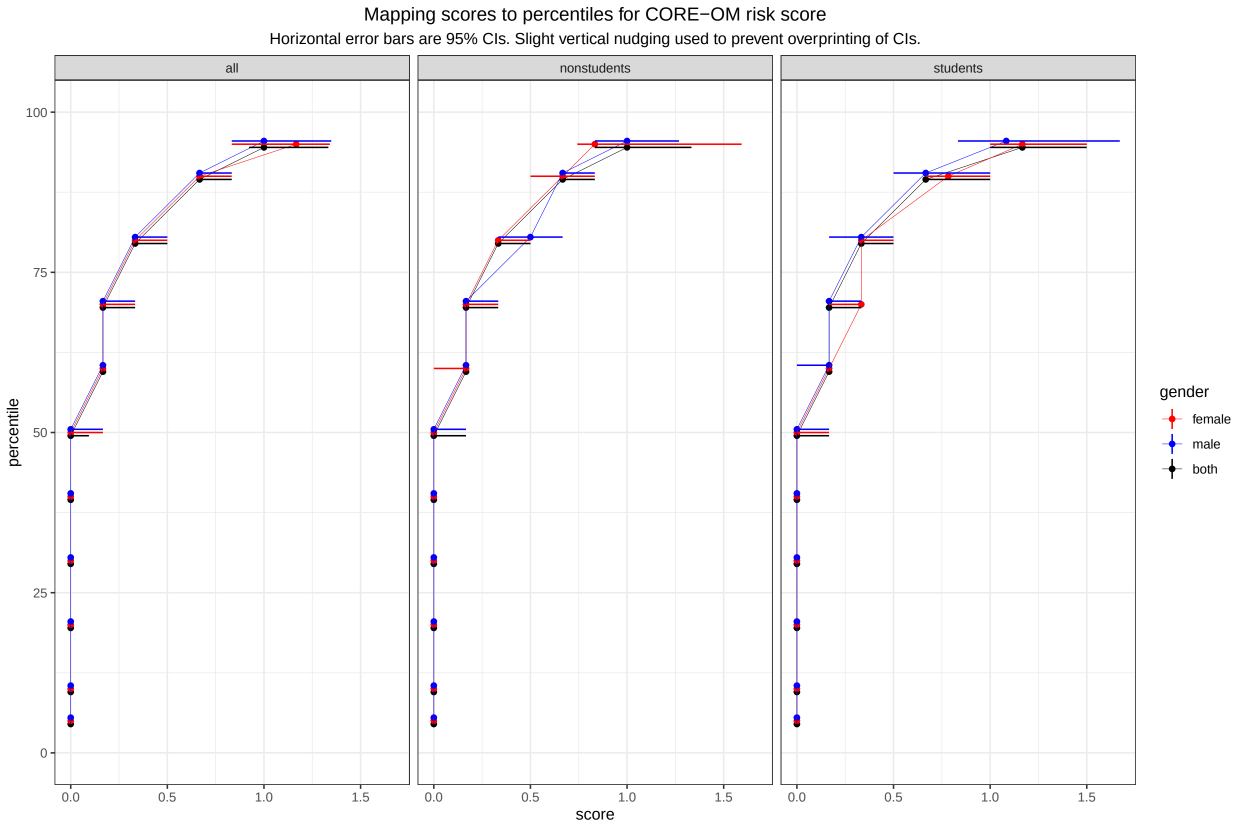

Interestingly no marked gender differences for any centiles.

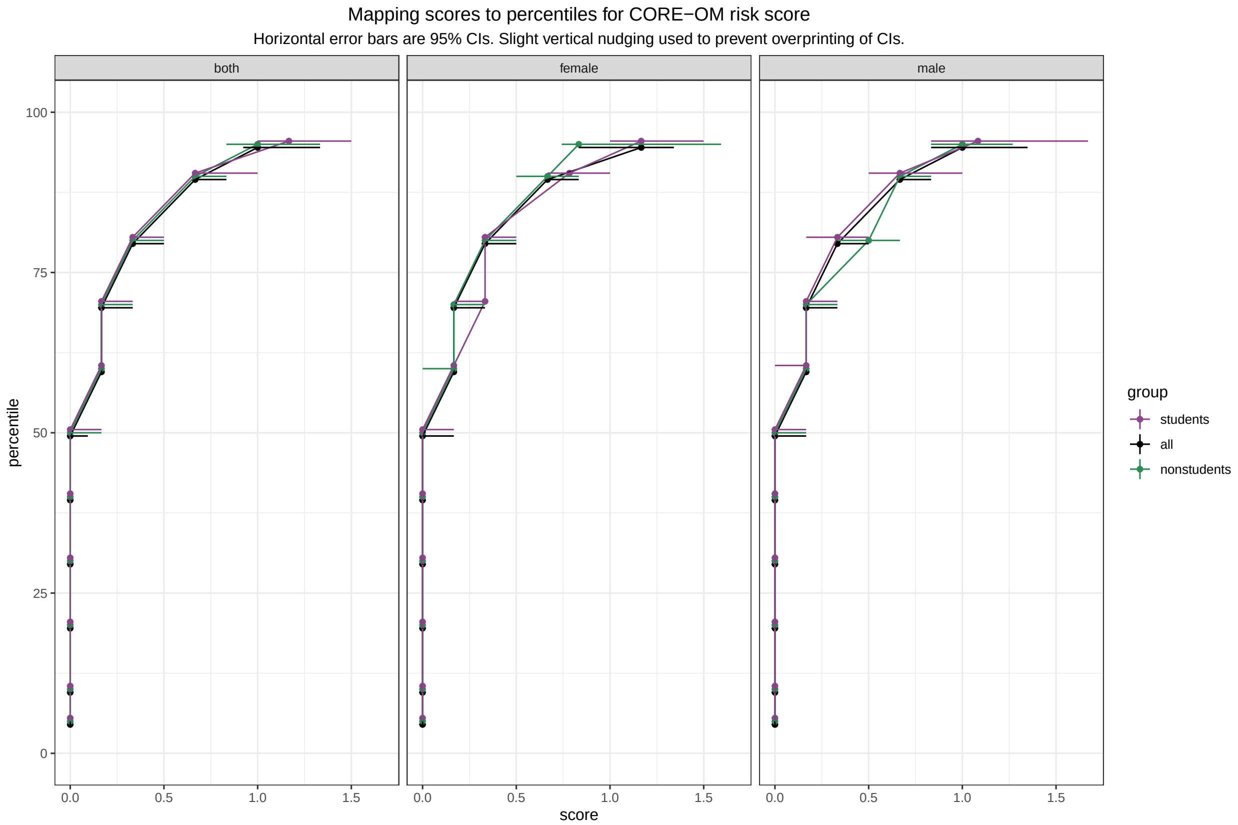

Here is the table for these CORE-OM risk centiles.

group	gender	5th	10th	20th	30th	40th	50th	60th	70th	80th	90th	95th
all	both	0.00[0.00, 0.00]	0.00[0.00, 0.00]	0.00[0.00, 0.00]	0.00[0.00, 0.00]	0.00[0.00, 0.00]	0.00[0.00, 0.09]	0.17[0.17, 0.17]	0.17[0.17, 0.33]	0.33[0.33, 0.50]	0.67[0.67, 0.83]	1.00[0.92, 1.33]
all	female	0.00[0.00, 0.00]	0.00[0.00, 0.00]	0.00[0.00, 0.00]	0.00[0.00, 0.00]	0.00[0.00, 0.00]	0.00[0.00, 0.17]	0.17[0.17, 0.17]	0.17[0.17, 0.33]	0.33[0.33, 0.50]	0.67[0.67, 0.83]	1.17[0.83, 1.34]
all	male	0.00[0.00, 0.00]	0.00[0.00, 0.00]	0.00[0.00, 0.00]	0.00[0.00, 0.00]	0.00[0.00, 0.00]	0.00[0.00, 0.17]	0.17[0.17, 0.17]	0.17[0.17, 0.33]	0.33[0.33, 0.50]	0.67[0.67, 0.83]	1.00[0.83, 1.35]
nonstudents	both	0.00[0.00, 0.00]	0.00[0.00, 0.00]	0.00[0.00, 0.00]	0.00[0.00, 0.00]	0.00[0.00, 0.00]	0.00[0.00, 0.17]	0.17[0.17, 0.17]	0.17[0.17, 0.33]	0.33[0.33, 0.50]	0.67[0.67, 0.83]	1.00[0.83, 1.33]
nonstudents	female	0.00[0.00, 0.00]	0.00[0.00, 0.00]	0.00[0.00, 0.00]	0.00[0.00, 0.00]	0.00[0.00, 0.00]	0.00[0.00, 0.00]	0.17[0.00, 0.17]	0.17[0.17, 0.33]	0.33[0.33, 0.50]	0.67[0.50, 0.83]	0.83[0.74, 1.59]
nonstudents	male	0.00[0.00, 0.00]	0.00[0.00, 0.00]	0.00[0.00, 0.00]	0.00[0.00, 0.00]	0.00[0.00, 0.00]	0.00[0.00, 0.17]	0.17[0.17, 0.17]	0.17[0.17, 0.33]	0.50[0.33, 0.67]	0.67[0.67, 0.83]	1.00[0.83, 1.27]
students	both	0.00[0.00, 0.00]	0.00[0.00, 0.00]	0.00[0.00, 0.00]	0.00[0.00, 0.00]	0.00[0.00, 0.00]	0.00[0.00, 0.17]	0.17[0.17, 0.17]	0.17[0.17, 0.33]	0.33[0.33, 0.50]	0.67[0.67, 1.00]	1.17[1.00, 1.50]
students	female	0.00[0.00, 0.00]	0.00[0.00, 0.00]	0.00[0.00, 0.00]	0.00[0.00, 0.00]	0.00[0.00, 0.00]	0.00[0.00, 0.17]	0.17[0.17, 0.17]	0.33[0.17, 0.33]	0.33[0.33, 0.50]	0.78[0.67, 1.00]	1.17[1.00, 1.50]
students	male	0.00[0.00, 0.00]	0.00[0.00, 0.00]	0.00[0.00, 0.00]	0.00[0.00, 0.00]	0.00[0.00, 0.00]	0.00[0.00, 0.17]	0.17[0.00, 0.17]	0.17[0.17, 0.33]	0.33[0.17, 0.50]	0.67[0.50, 1.00]	1.08[0.83, 1.67]

Centiles for EQ index score

Should be different again. Mainly high scorers.

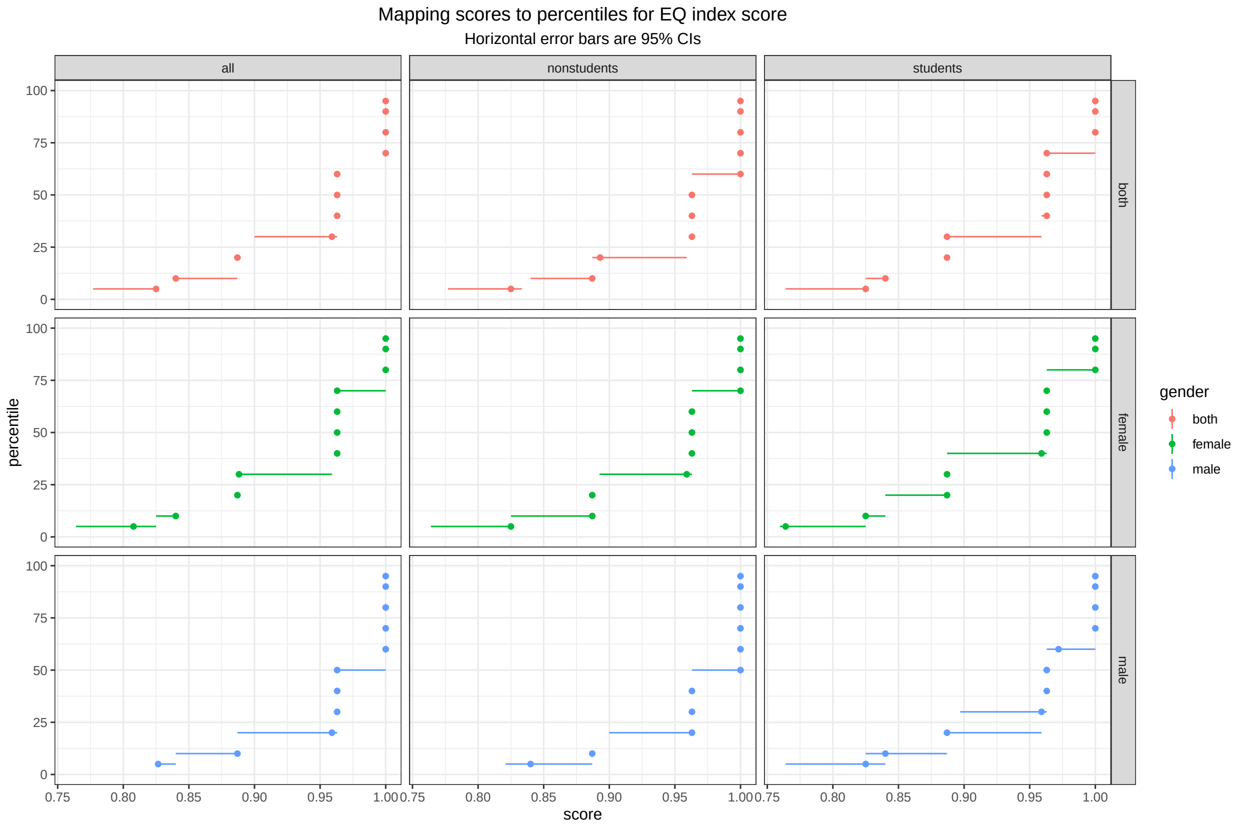

Indeed, radically different: overall 35.9% score the “fully OK” 1.0.

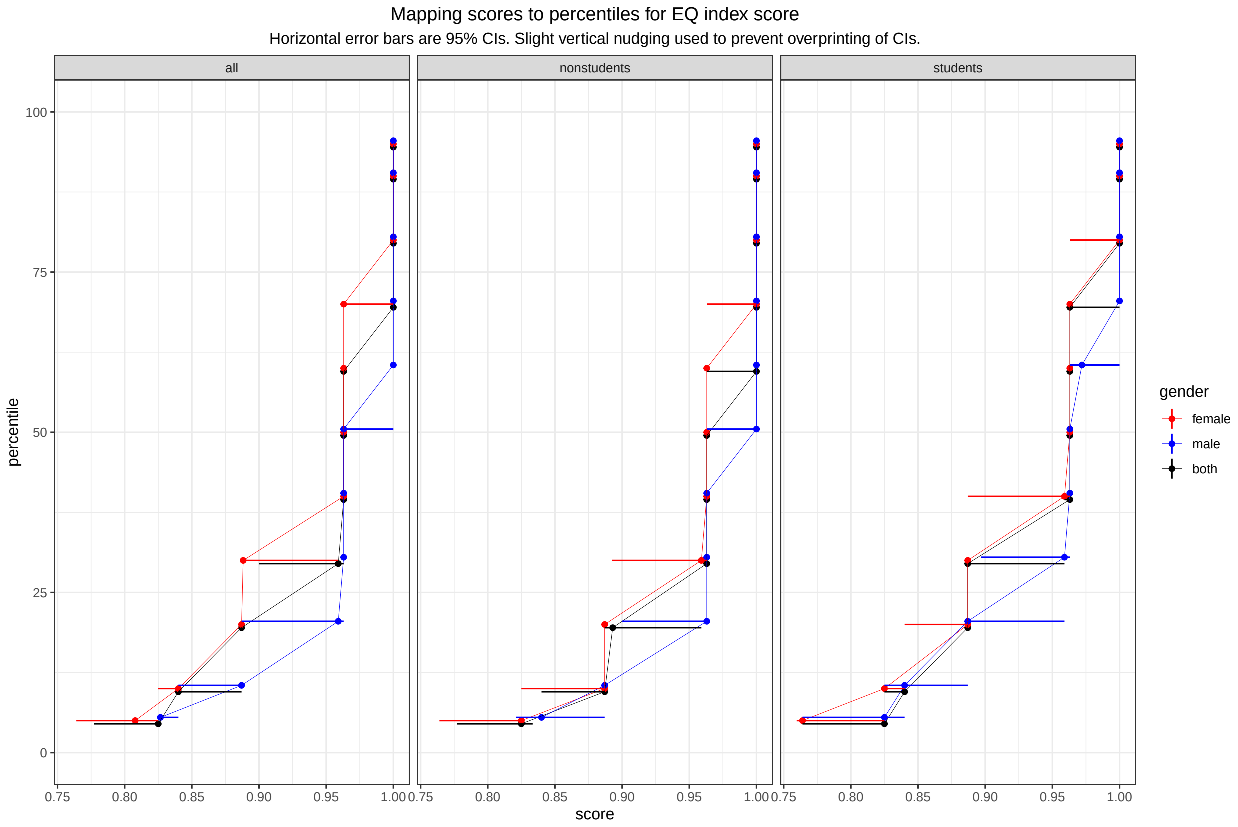

No clear gender differences but a real mix of wide and essentially zero width CIs partly because there only 25 distinct values are observed across the whole dataset.

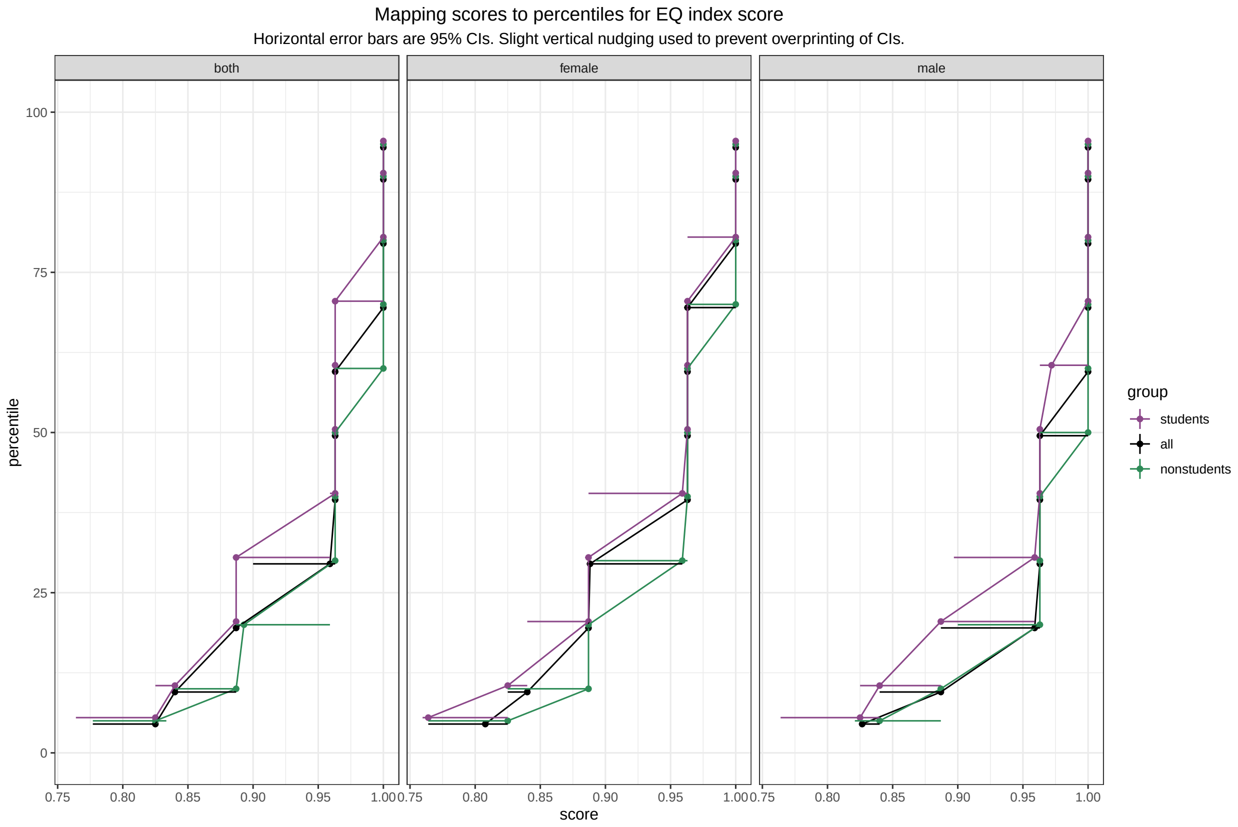

Similar issue. Here is the table for these (EQ index) centiles.

group	gender	5th	10th	20th	30th	40th	50th	60th	70th	80th	90th	95th
all	both	0.82[0.78, 0.82]	0.84[0.84, 0.89]	0.89[0.89, 0.89]	0.96[0.90, 0.96]	0.96[0.96, 0.96]	0.96[0.96, 0.96]	0.96[0.96, 0.96]	1.00[1.00, 1.00]	1.00[1.00, 1.00]	1.00[1.00, 1.00]	1.00[1.00, 1.00]
all	female	0.81[0.76, 0.82]	0.84[0.82, 0.84]	0.89[0.89, 0.89]	0.89[0.89, 0.96]	0.96[0.96, 0.96]	0.96[0.96, 0.96]	0.96[0.96, 0.96]	0.96[0.96, 1.00]	1.00[1.00, 1.00]	1.00[1.00, 1.00]	1.00[1.00, 1.00]
all	male	0.83[0.82, 0.84]	0.89[0.84, 0.89]	0.96[0.89, 0.96]	0.96[0.96, 0.96]	0.96[0.96, 0.96]	0.96[0.96, 1.00]	1.00[1.00, 1.00]	1.00[1.00, 1.00]	1.00[1.00, 1.00]	1.00[1.00, 1.00]	1.00[1.00, 1.00]
nonstudents	both	0.82[0.78, 0.83]	0.89[0.84, 0.89]	0.89[0.89, 0.96]	0.96[0.96, 0.96]	0.96[0.96, 0.96]	0.96[0.96, 0.96]	1.00[0.96, 1.00]	1.00[1.00, 1.00]	1.00[1.00, 1.00]	1.00[1.00, 1.00]	1.00[1.00, 1.00]
nonstudents	female	0.82[0.76, 0.82]	0.89[0.82, 0.89]	0.89[0.89, 0.89]	0.96[0.89, 0.96]	0.96[0.96, 0.96]	0.96[0.96, 0.96]	0.96[0.96, 0.96]	1.00[0.96, 1.00]	1.00[1.00, 1.00]	1.00[1.00, 1.00]	1.00[1.00, 1.00]
nonstudents	male	0.84[0.82, 0.89]	0.89[0.89, 0.89]	0.96[0.90, 0.96]	0.96[0.96, 0.96]	0.96[0.96, 0.96]	1.00[0.96, 1.00]	1.00[1.00, 1.00]	1.00[1.00, 1.00]	1.00[1.00, 1.00]	1.00[1.00, 1.00]	1.00[1.00, 1.00]
students	both	0.82[0.76, 0.82]	0.84[0.82, 0.84]	0.89[0.89, 0.89]	0.89[0.89, 0.96]	0.96[0.96, 0.96]	0.96[0.96, 0.96]	0.96[0.96, 0.96]	0.96[0.96, 1.00]	1.00[1.00, 1.00]	1.00[1.00, 1.00]	1.00[1.00, 1.00]
students	female	0.76[0.76, 0.82]	0.82[0.82, 0.84]	0.89[0.84, 0.89]	0.89[0.89, 0.89]	0.96[0.89, 0.96]	0.96[0.96, 0.96]	0.96[0.96, 0.96]	0.96[0.96, 0.96]	1.00[0.96, 1.00]	1.00[1.00, 1.00]	1.00[1.00, 1.00]
students	male	0.82[0.76, 0.84]	0.84[0.82, 0.89]	0.89[0.89, 0.96]	0.96[0.90, 0.96]	0.96[0.96, 0.96]	0.96[0.96, 0.96]	0.97[0.96, 1.00]	1.00[1.00, 1.00]	1.00[1.00, 1.00]	1.00[1.00, 1.00]	1.00[1.00, 1.00]

Centiles for EQ VAS

Should be different again.

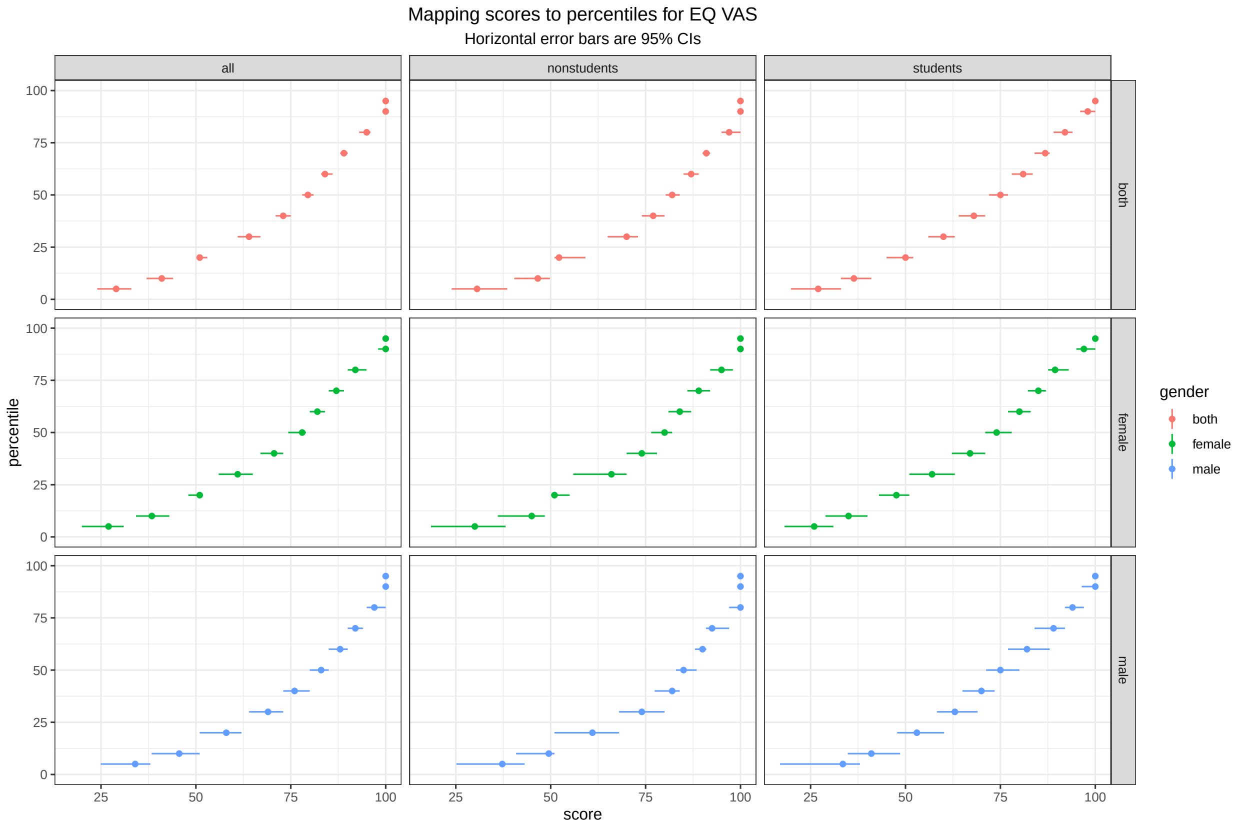

Indeed, different again: overall 13.8% score 100% and 94 distinct values.

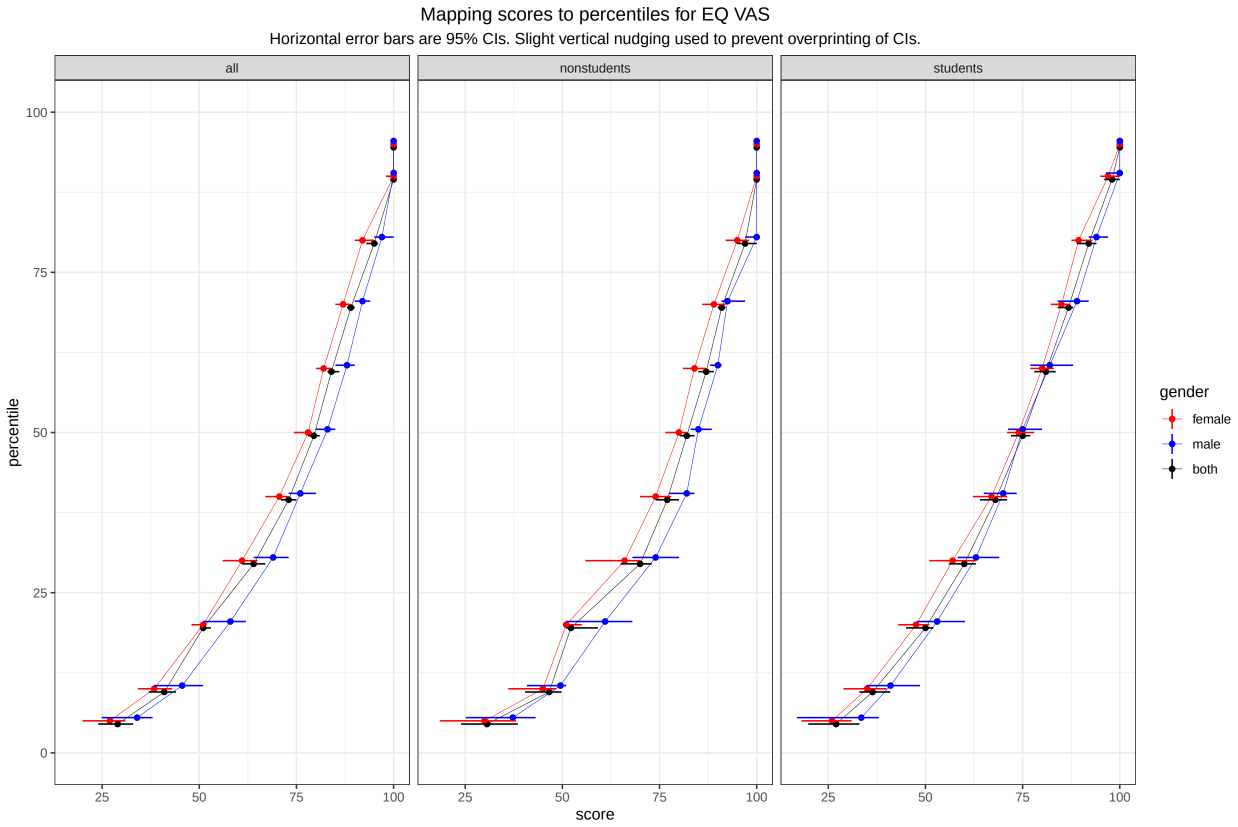

Interestingly, in the student group gender differences look to be within sampling vagaries judging by the CIs (except for the 30th centile where there is a clear differnce with the score for the centile higher for the men than the women). However, there is clear separation by gender in the non-student group and, though less strong, in the total sample.

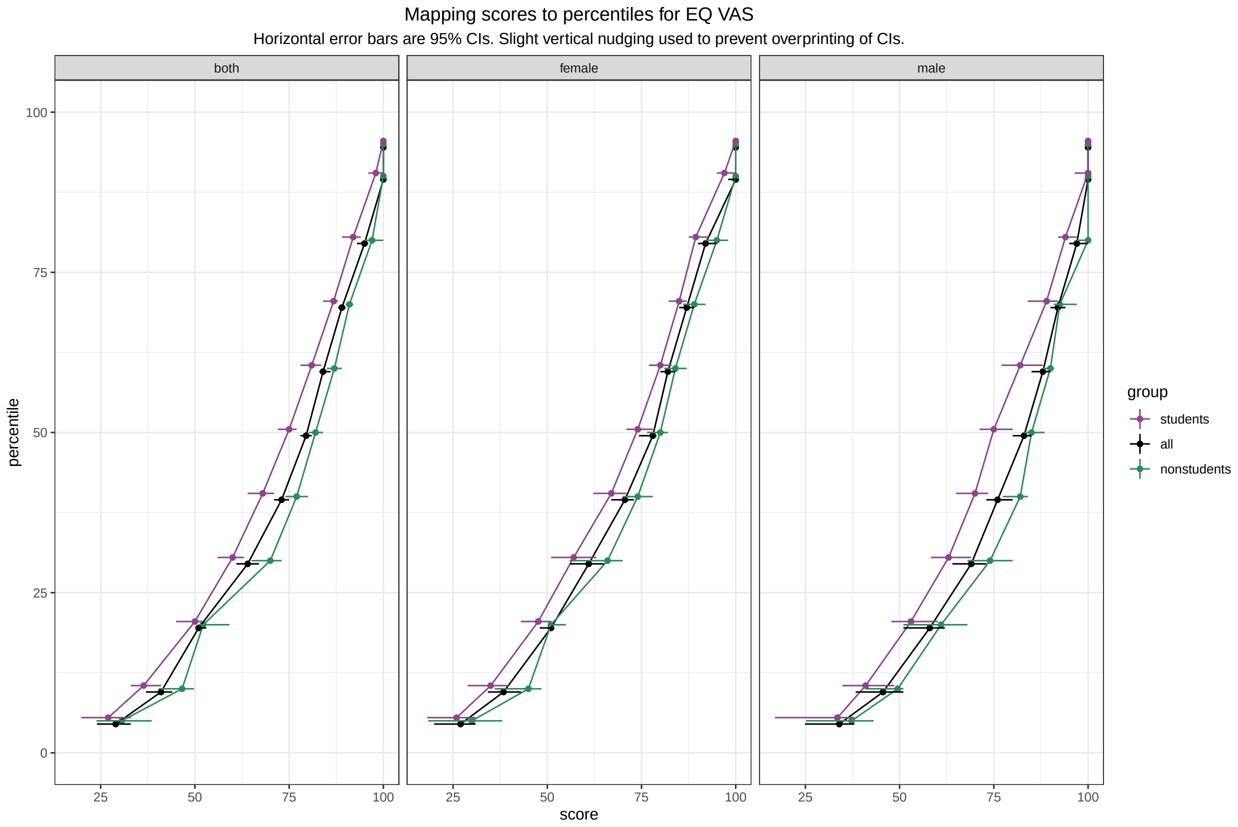

Above the 5th centile there is clear separation of the centiles by group in each gender and even more clearly statistically significantly in the total sample. Here is the table for these (EQ VAS) centiles.

group	gender	5th	10th	20th	30th	40th	50th	60th	70th	80th	90th	95th
all	both	29.00[24.[illegible], 33.00]	[illegible][37.[illegible], 44.00]	[illegible][51.[illegible], 53.00]	[illegible][61.[illegible], 67.00]	[illegible][71.[illegible], 75.00]	[illegible][78.[illegible], 81.00]	[illegible][83.[illegible], 86.00]	[illegible][88.[illegible], 90.00]	[illegible][93.[illegible], 96.00]	100.00[100.[illegible], 100.00]	100.00[100.00, 100.00]
all	female	27.00[19.[illegible], 31.00]	[illegible][34.[illegible], 43.00]	[illegible][48.[illegible], 51.00]	[illegible][56.[illegible], 65.00]	[illegible][67.[illegible], 73.00]	[illegible][74.[illegible], 79.00]	[illegible][80.[illegible], 84.00]	[illegible][85.[illegible], 89.00]	[illegible][90.[illegible], 94.95]	100.00[98.[illegible], 100.00]	100.00[100.00, 100.00]
all	male	34.00[24.[illegible], 38.00]	[illegible][38.[illegible], 51.00]	[illegible][51.[illegible], 62.00]	[illegible][64.[illegible], 73.00]	[illegible][73.[illegible], 80.00]	[illegible][80.[illegible], 85.00]	[illegible][85.[illegible], 90.00]	[illegible][90.[illegible], 94.00]	[illegible][95.[illegible], 100.00]	100.00[100.[illegible], 100.00]	100.00[100.00, 100.00]
nonstudents	both	30.60[23.[illegible], 38.56]	[illegible][40.[illegible], 49.79]	[illegible][51.[illegible], 59.15]	[illegible][65.[illegible], 73.00]	[illegible][74.[illegible], 80.00]	[illegible][80.[illegible], 84.00]	[illegible][85.[illegible], 89.00]	[illegible][90.[illegible], 92.00]	[illegible][95.[illegible], 100.00]	100.00[100.[illegible], 100.00]	100.00[100.00, 100.00]
nonstudents	female	30.00[18.[illegible], 38.09]	[illegible][36.[illegible], 48.45]	[illegible][51.[illegible], 55.00]	[illegible][55.[illegible], 70.00]	[illegible][70.[illegible], 78.00]	[illegible][76.[illegible], 82.00]	[illegible][81.[illegible], 87.00]	[illegible][86.[illegible], 92.00]	[illegible][92.[illegible], 98.00]	100.00[100.[illegible], 100.00]	100.00[100.00, 100.00]
nonstudents	male	37.25[25.[illegible], 43.13]	[illegible][40.[illegible], 51.00]	[illegible][51.[illegible], 68.00]	[illegible][68.[illegible], 80.00]	[illegible][77.[illegible], 84.00]	[illegible][83.[illegible], 88.44]	[illegible][88.[illegible], 91.00]	[illegible][90.[illegible], 97.00]	100.00[97.[illegible], 100.00]	100.00[100.[illegible], 100.00]	100.00[100.00, 100.00]
students	both	27.00[19.[illegible], 33.00]	[illegible][33.[illegible], 41.00]	[illegible][45.[illegible], 52.03]	[illegible][56.[illegible], 63.00]	[illegible][64.[illegible], 71.00]	[illegible][72.[illegible], 77.00]	[illegible][78.[illegible], 83.52]	[illegible][84.[illegible], 88.00]	[illegible][89.[illegible], 94.00]	98.00[96.[illegible], 100.00]	100.00[100.00, 100.00]
students	female	25.95[18.[illegible], 31.00]	[illegible][28.[illegible], 40.00]	[illegible][43.[illegible], 51.00]	[illegible][51.[illegible], 63.00]	[illegible][62.[illegible], 71.00]	[illegible][71.[illegible], 78.00]	[illegible][77.[illegible], 83.00]	[illegible][82.[illegible], 87.00]	[illegible][87.[illegible], 93.00]	97.00[95.[illegible], 100.00]	100.00[100.00, 100.00]
students	male	33.50[16.[illegible], 38.00]	[illegible][34.[illegible], 48.59]	[illegible][47.[illegible], 60.18]	[illegible][58.[illegible], 69.00]	[illegible][65.[illegible], 73.50]	[illegible][71.[illegible], 80.00]	[illegible][77.[illegible], 88.00]	[illegible][84.[illegible], 92.00]	[illegible][92.[illegible], 97.00]	100.00[96.[illegible], 100.00]	100.00[100.00, 100.00]

Centiles for WGO negative

Should be different again. Very limited score range will make centiles meaningless really.

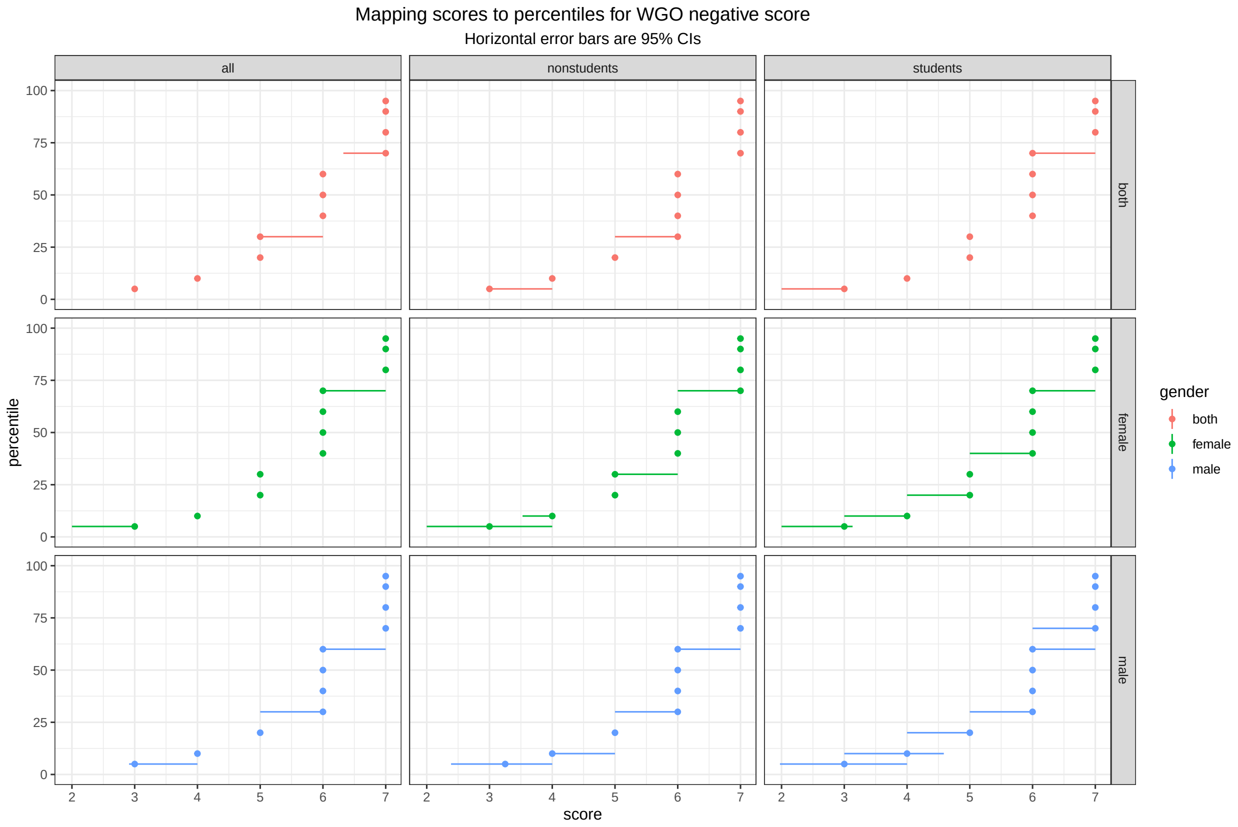
Mapping scores to percentiles for WGO negative score
Horizontal error bars are 95% CIs
all
nonstudents
students
both
female
male
percentile
score
gender
both
female
male

As expected!

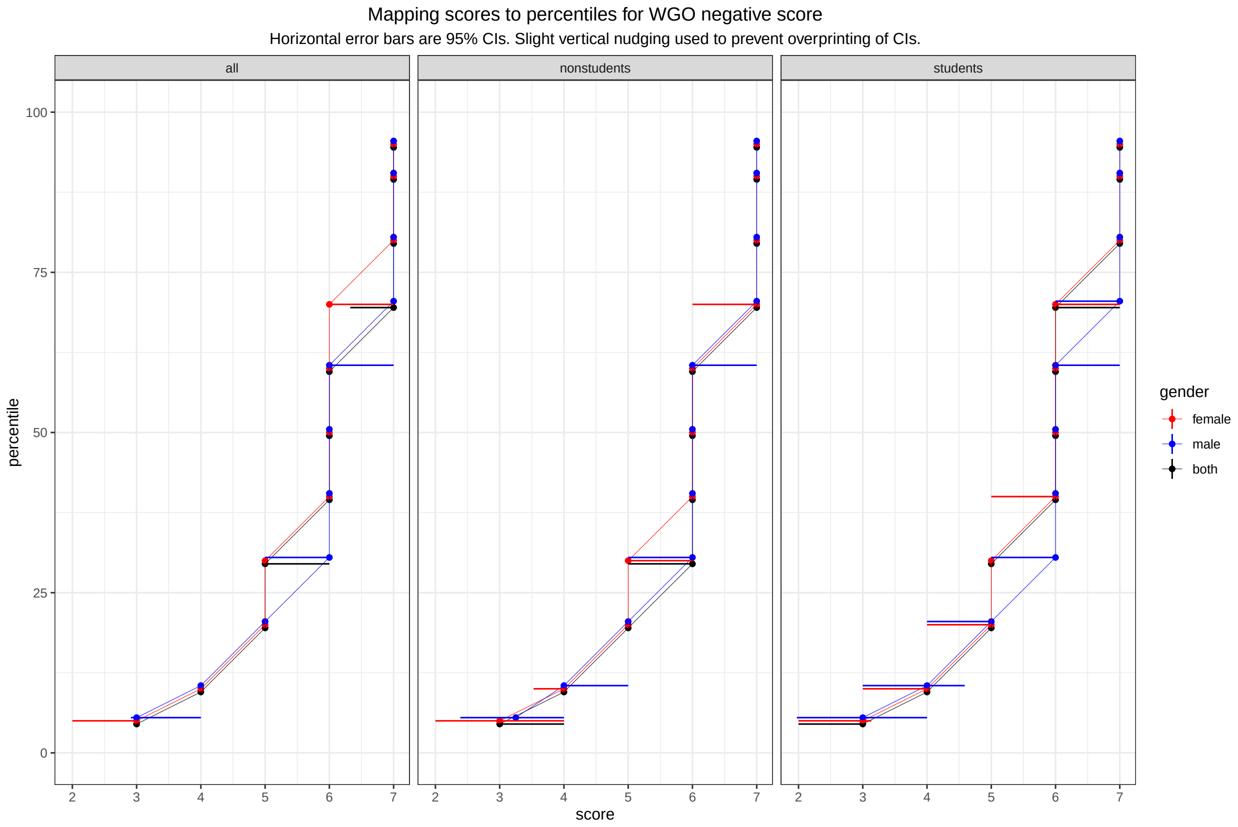

No clear gender differences but again this is partly because there is no fine discrimination with such a limited number of possible scores.

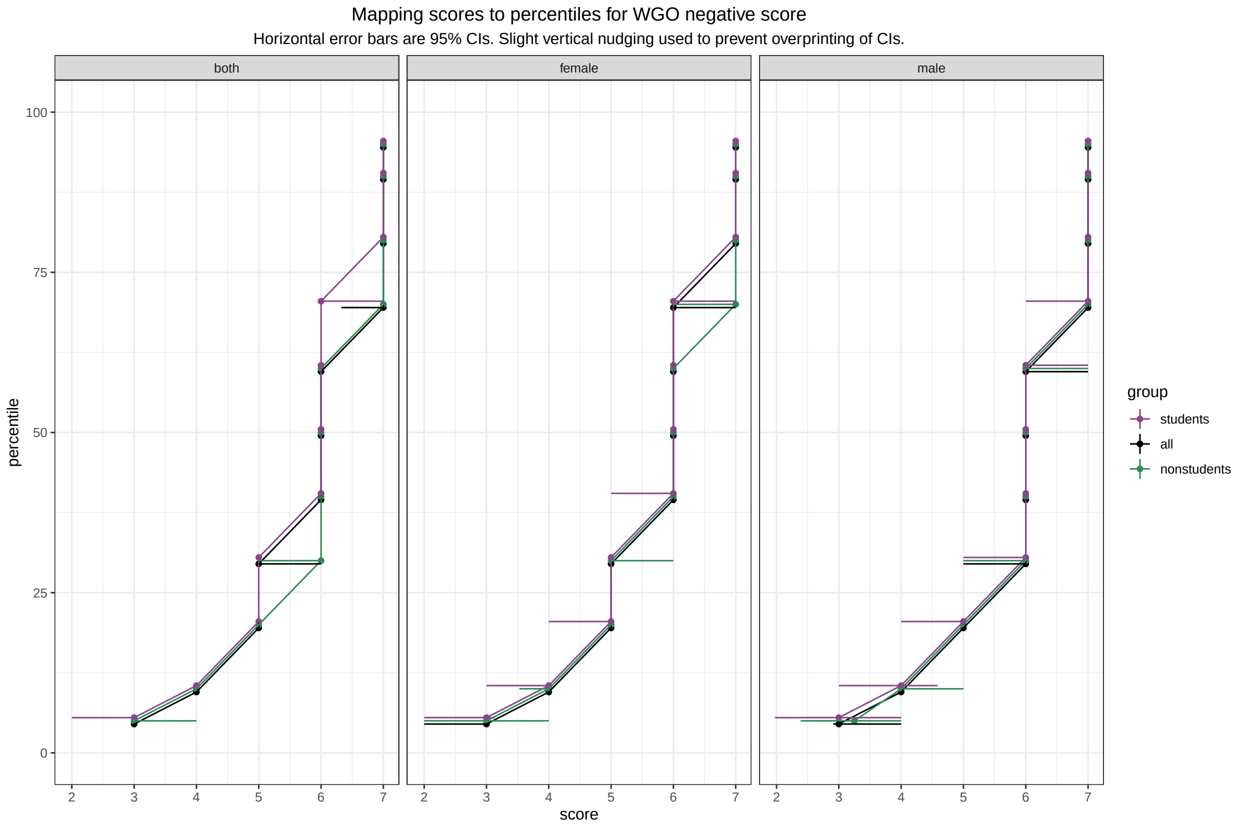

Same issue. Here is the table for these (WGO negative) centiles but that's really only to confirm that these aren't really useful.

group	gender	5th	10th	20th	30th	40th	50th	60th	70th	80th	90th	95th
all	both	3.00[3.00, 3.00]	4.00[4.00, 4.00]	5.00[5.00, 5.00]	5.00[5.00, 6.00]	6.00[6.00, 6.00]	6.00[6.00, 6.00]	6.00[6.00, 6.00]	7.00[6.32, 7.00]	7.00[7.00, 7.00]	7.00[7.00, 7.00]	7.00[7.00, 7.00]
all	female	3.00[2.00, 3.00]	4.00[4.00, 4.00]	5.00[5.00, 5.00]	5.00[5.00, 5.00]	6.00[6.00, 6.00]	6.00[6.00, 6.00]	6.00[6.00, 6.00]	6.00[6.00, 7.00]	7.00[7.00, 7.00]	7.00[7.00, 7.00]	7.00[7.00, 7.00]
all	male	3.00[2.91, 4.00]	4.00[4.00, 4.00]	5.00[5.00, 5.00]	6.00[5.00, 6.00]	6.00[6.00, 6.00]	6.00[6.00, 6.00]	6.00[6.00, 7.00]	7.00[7.00, 7.00]	7.00[7.00, 7.00]	7.00[7.00, 7.00]	7.00[7.00, 7.00]
nonstudents	both	3.00[2.97, 4.00]	4.00[4.00, 4.00]	5.00[5.00, 5.00]	6.00[5.00, 6.00]	6.00[6.00, 6.00]	6.00[6.00, 6.00]	6.00[6.00, 6.00]	7.00[7.00, 7.00]	7.00[7.00, 7.00]	7.00[7.00, 7.00]	7.00[7.00, 7.00]
nonstudents	female	3.00[2.00, 4.00]	4.00[3.53, 4.00]	5.00[5.00, 5.00]	5.00[5.00, 6.00]	6.00[6.00, 6.00]	6.00[6.00, 6.00]	6.00[6.00, 6.00]	7.00[6.00, 7.00]	7.00[7.00, 7.00]	7.00[7.00, 7.00]	7.00[7.00, 7.00]
nonstudents	male	3.25[2.39, 4.00]	4.00[4.00, 5.00]	5.00[5.00, 5.00]	6.00[5.00, 6.00]	6.00[6.00, 6.00]	6.00[6.00, 6.00]	6.00[6.00, 7.00]	7.00[7.00, 7.00]	7.00[7.00, 7.00]	7.00[7.00, 7.00]	7.00[7.00, 7.00]
students	both	3.00[2.00, 3.00]	4.00[4.00, 4.00]	5.00[5.00, 5.00]	5.00[5.00, 5.00]	6.00[6.00, 6.00]	6.00[6.00, 6.00]	6.00[6.00, 6.00]	6.00[6.00, 7.00]	7.00[7.00, 7.00]	7.00[7.00, 7.00]	7.00[7.00, 7.00]
students	female	3.00[2.00, 3.13]	4.00[3.00, 4.00]	5.00[4.00, 5.00]	5.00[5.00, 5.00]	6.00[5.00, 6.00]	6.00[6.00, 6.00]	6.00[6.00, 6.00]	6.00[6.00, 7.00]	7.00[7.00, 7.00]	7.00[7.00, 7.00]	7.00[7.00, 7.00]
students	male	3.00[1.97, 4.00]	4.00[3.00, 4.59]	5.00[4.00, 5.00]	6.00[5.00, 6.00]	6.00[6.00, 6.00]	6.00[6.00, 6.00]	6.00[6.00, 7.00]	7.00[6.00, 7.00]	7.00[7.00, 7.00]	7.00[7.00, 7.00]	7.00[7.00, 7.00]

Centiles for WGO positive

Again the very limited score range will make centiles meaningless.

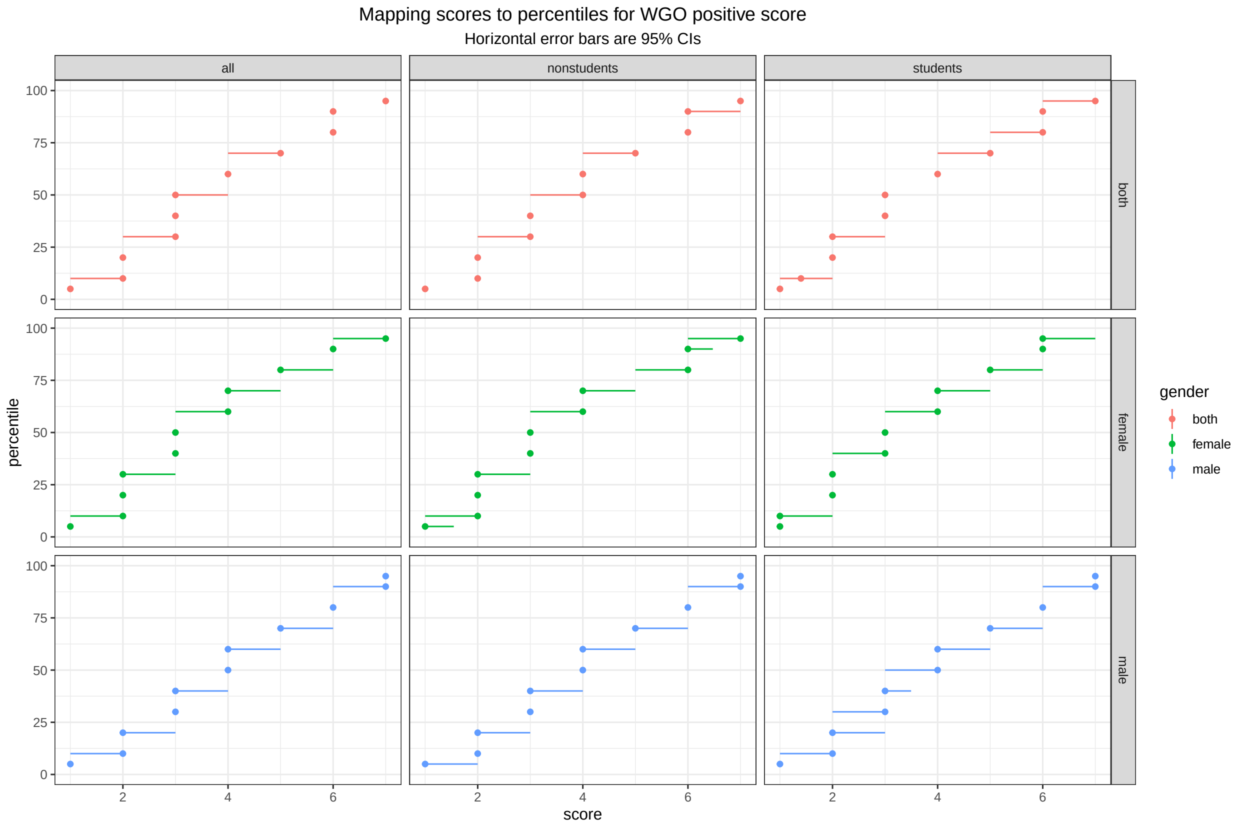

A bit more spread but essentially as we knew: this can't produce very useful centiles.

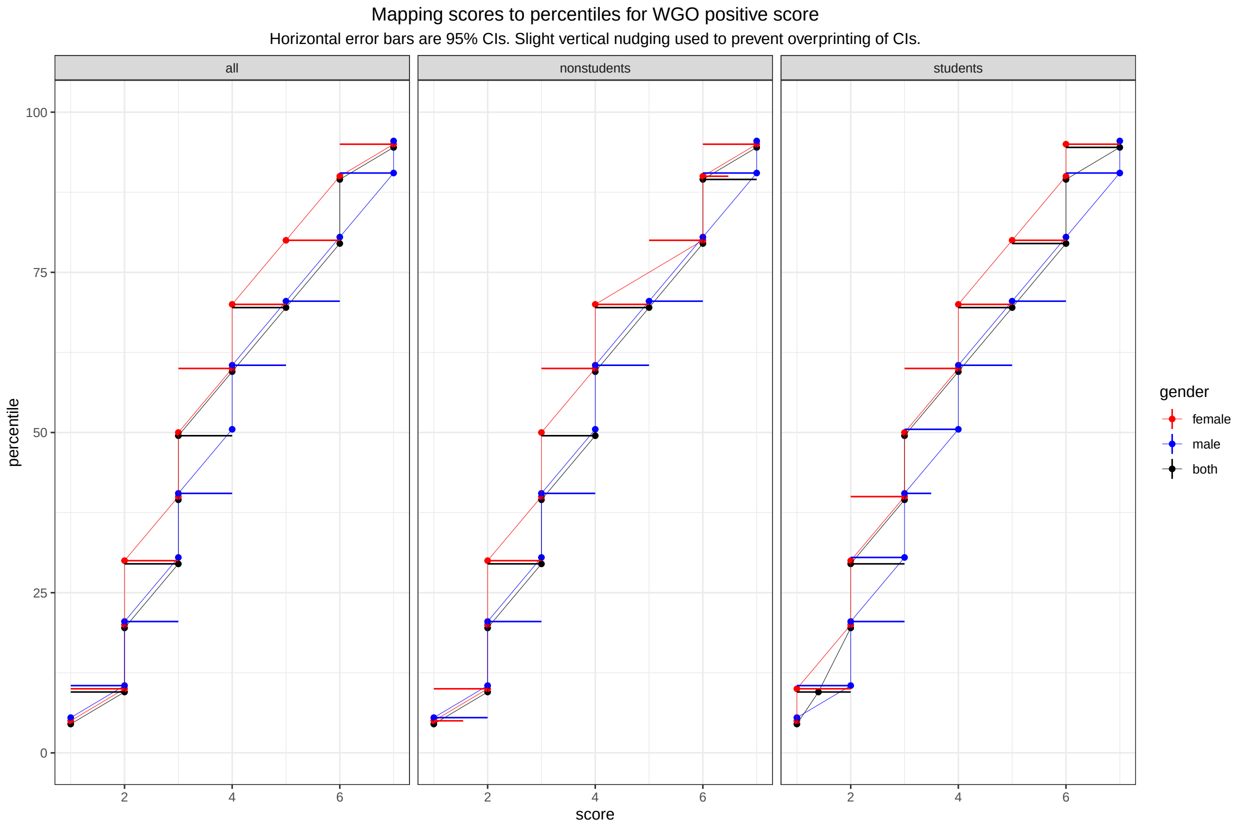

A hint of a gender difference but not outside the imprecision of sampling vagaries given the limited score range.

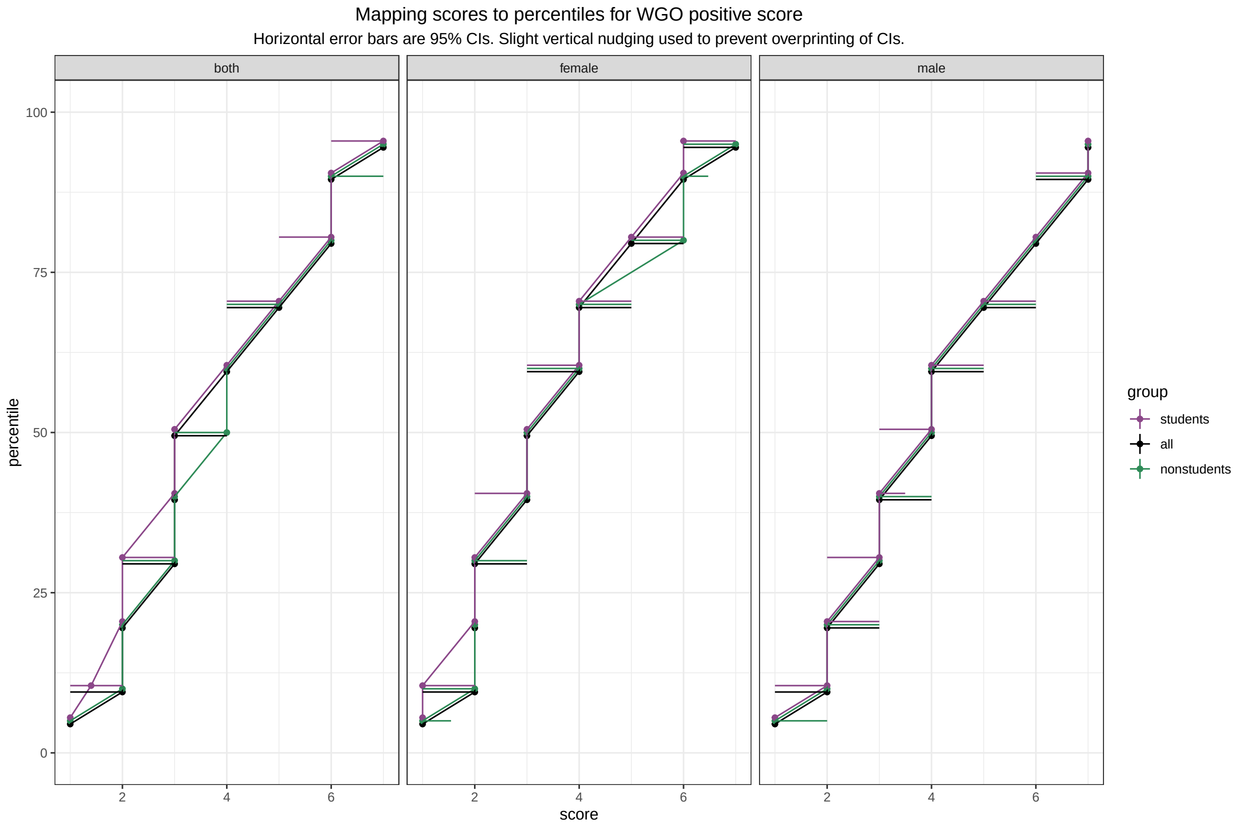

Same issue. Again, here are the WGO positive centiles but again, only to confirm that these aren't really useful.

group	gender	5th	10th	20th	30th	40th	50th	60th	70th	80th	90th	95th
all	both	1.00[1.00, 1.00]	2.00[1.00, 2.00]	2.00[2.00, 2.00]	3.00[2.00, 3.00]	3.00[3.00, 3.00]	3.00[3.00, 4.00]	4.00[4.00, 4.00]	5.00[4.00, 5.00]	6.00[6.00, 6.00]	6.00[6.00, 6.00]	7.00[7.00, 7.00]
all	female	1.00[1.00, 1.00]	2.00[1.00, 2.00]	2.00[2.00, 2.00]	2.00[2.00, 3.00]	3.00[3.00, 3.00]	3.00[3.00, 3.00]	4.00[3.00, 4.00]	4.00[4.00, 5.00]	5.00[5.00, 6.00]	6.00[6.00, 6.00]	7.00[6.00, 7.00]
all	male	1.00[1.00, 1.00]	2.00[1.00, 2.00]	2.00[2.00, 3.00]	3.00[3.00, 3.00]	3.00[3.00, 4.00]	4.00[4.00, 4.00]	4.00[4.00, 5.00]	5.00[5.00, 6.00]	6.00[6.00, 6.00]	7.00[6.00, 7.00]	7.00[7.00, 7.00]
nonstudents	both	1.00[1.00, 1.00]	2.00[2.00, 2.00]	2.00[2.00, 2.00]	3.00[2.00, 3.00]	3.00[3.00, 3.00]	4.00[3.00, 4.00]	4.00[4.00, 4.00]	5.00[4.00, 5.00]	6.00[6.00, 6.00]	6.00[6.00, 7.00]	7.00[7.00, 7.00]
nonstudents	female	1.00[1.00, 1.55]	2.00[1.00, 2.00]	2.00[2.00, 2.00]	2.00[2.00, 3.00]	3.00[3.00, 3.00]	3.00[3.00, 3.00]	4.00[3.00, 4.00]	4.00[4.00, 5.00]	6.00[5.00, 6.00]	6.00[6.00, 6.47]	7.00[6.00, 7.00]
nonstudents	male	1.00[1.00, 2.00]	2.00[1.94, 2.00]	2.00[2.00, 3.00]	3.00[3.00, 3.00]	3.00[3.00, 4.00]	4.00[4.00, 4.00]	4.00[4.00, 5.00]	5.00[5.00, 6.00]	6.00[6.00, 6.00]	7.00[6.00, 7.00]	7.00[7.00, 7.00]
students	both	1.00[1.00, 1.00]	1.40[1.00, 2.00]	2.00[2.00, 2.00]	2.00[2.00, 3.00]	3.00[3.00, 3.00]	3.00[3.00, 3.00]	4.00[4.00, 4.00]	5.00[4.00, 5.00]	6.00[5.00, 6.00]	6.00[6.00, 6.00]	7.00[6.00, 7.00]
students	female	1.00[1.00, 1.00]	1.00[1.00, 2.00]	2.00[2.00, 2.00]	2.00[2.00, 2.00]	3.00[2.00, 3.00]	3.00[3.00, 3.00]	4.00[3.00, 4.00]	4.00[4.00, 5.00]	5.00[5.00, 6.00]	6.00[6.00, 6.00]	6.00[6.00, 7.00]
students	male	1.00[1.00, 1.00]	2.00[1.00, 2.00]	2.00[2.00, 3.00]	3.00[2.00, 3.00]	3.00[3.00, 3.50]	4.00[3.00, 4.00]	4.00[4.00, 5.00]	5.00[5.00, 6.00]	6.00[6.00, 6.00]	7.00[6.00, 7.00]	7.00[7.00, 7.00]
